# Associations between COVID-19 Risk Perceptions and Mental Health, Wellbeing, and Risk Behaviours

**DOI:** 10.1080/13669877.2022.2127849

**Published:** 2022-11-12

**Authors:** Maddy L. Dyer, Hannah M. Sallis, Jasmine N. Khouja, Sarah Dryhurst, Marcus R. Munafò

**Affiliations:** 1 School of Psychological Science, University of Bristol, Bristol, UK; 2 Medical Research Council Integrative Epidemiology Unit at the University of Bristol, Bristol, UK; 3 Centre for Academic Mental Health, Population Health Sciences, Bristol Medical School, University of Bristol, Bristol, UK; 4 Winton Centre for Risk and Evidence Communication, University of Cambridge, Cambridge, UK; 5 National Institute for Health Research Bristol Biomedical Research Centre, University Hospitals Bristol NHS Foundation Trust, Bristol, UK

**Keywords:** coronavirus, risk perception, risk behaviour, mental health, ALSPAC

## Abstract

**Background:**

Mental health has worsened, and substance use has increased for some people during the coronavirus (COVID-19) pandemic. Some cross-sectional studies suggest that higher COVID-19 risk perceptions are related to poorer mental health and greater risk behaviours (e.g., substance use). However, longitudinal and genetic data are needed to help to reduce the likelihood of reverse causality.

**Methods:**

We used cross-sectional, longitudinal, and polygenic risk score (PRS; for anxiety, depression, wellbeing) data from the Avon Longitudinal Study of Parents and Children (ALSPAC). We examined cross-sectional and prospective longitudinal associations between COVID-19 risk perceptions (i.e., cognitive, affective, self, other, and a combined ‘holistic’ measure) and mental health (i.e., anxiety, depression), wellbeing, and risk behaviours. Pandemic (April-July 2020) and pre-pandemic (2003-2017) data (ns = 233-5,115) were included.

**Results:**

Higher COVID-19 risk perceptions (holistic) were associated with anxiety (OR 2.78, 95% confidence interval [CI] 2.20 to 3.52), depression (OR 1.65, 95% CI 1.24 to 2.18), low wellbeing (OR 1.76, 95% CI 1.45 to 2.13), and increased alcohol use (OR 1.46, 95% CI 1.24 to 1.72). Higher COVID-19 risk perceptions were also associated with self-isolating given a suspected COVID-19 infection (OR 1.74, 95% CI 1.13 to 2.68), and less face-to-face contact (OR 0.83, 95% CI 0.70 to 0.98) and physical contact (OR 0.83, 95% CI 0.68 to 1.00). Pre-pandemic anxiety (OR 1.64, 95% CI 1.29 to 2.09) and low wellbeing (OR 1.41, 95% CI 1.15 to 1.74) were associated with higher COVID-19 risk perceptions. The depression PRS (*b* 0.21, 95% CI 0.02 to 0.40) and wellbeing PRS (*b* -0.29, 95% CI -0.48 to -0.09) were associated with higher and lower COVID-19 risk perceptions, respectively.

**Conclusions:**

Poorer mental health and wellbeing are associated with higher COVID-19 risk perceptions, and longitudinal and genetic data suggest that they may play a causal role in COVID-19 risk perceptions.

## Introduction

The coronavirus (COVID-19) pandemic was declared in March 2020 (World Health Organisation 2021a). As of November 2021, there have been over 250 million confirmed cases, including over 5 million deaths globally (World Health Organisation 2021b). The pandemic and mitigation measures have impacted mental health (Byrne, Barber, and Lim 2021); 60% of UK adults report that their mental health has deteriorated, and 36% report using alcohol or illegal drugs to cope (Mind 2020). Wellbeing has reduced, anxiety has almost doubled (from 13% to 24%) (Kwong et al. 2020), and approximately 25% of people report drinking alcohol and smoking more (Garnett et al. 2021, Tzu-Hsuan Chen 2020). Risk perceptions are subjective judgements about the characteristics, severity, and probability of a risk (Darker 2013). They can influence emotions and behaviours (Ferrer and Klein 2015, Paek and Hove 2017), and impact how governments and individuals respond to the pandemic (McCloskey and Heymann 2020). COVID-19 risk perceptions refer to the perceived likelihood of SARS-CoV-2 infection (cognitive COVID-19 risk perceptions) and worries about SARS-CoV-2 infection (affective COVID-19 risk perceptions) with holistic COVID-19 risk perceptions referring to these measures combined (Dryhurst et al. 2020, Schneider et al. 2021). COVID-19 risk perceptions may have contributed to the changes in mental health (e.g., anxiety, depression), wellbeing, and risk behaviours (e.g., alcohol use, smoking) observed during the pandemic.

In the opposite temporal direction, mental health, wellbeing, and risk behaviours could also influence COVID-19 risk perceptions. According to valence approaches, negative emotions lead to higher risk perceptions (Lerner and Keltner 2000). Therefore, pre-pandemic anxiety, depression, and low wellbeing may lead to increased risk perceptions about a new global pandemic. Furthermore, according to self-perception theory, behaviours affect thoughts and attitudes (Bem 1972). People may adjust their perception of risk to align with their behaviour if they cannot (or choose not to) adjust their behaviour, in order to reduce cognitive dissonance (Festinger 1957). For example, going to work rather than self-isolating following a COVID-19 diagnosis (e.g., for financial reasons) may lead to reduced risk perceptions. Understanding COVID-19 risk perceptions and their possible bidirectional associations with mental health, wellbeing, and risk behaviours is therefore crucial for informing pandemic preparedness and response efforts. This research has implications for risk communication and public health messaging during the current and future pandemics.

### Mental Health and Wellbeing

Mental health conditions, such as anxiety and depression, are disorders characterised by a combination of abnormal thoughts, emotions, and behaviours (World Health Organisation 2019). Cross-sectional studies have found associations between higher COVID-19 risk perceptions and poorer mental health. For example, Zhong and colleagues (2021) and Liu and colleagues (2020) found that COVID-19 risk perceptions (likelihood of infection) were associated with higher depressive states and anxiety levels, respectively. Similarly, COVID-19 risk perceptions (likelihood of infection or economic consequences from COVID-19, and COVID-19 threat) have been associated with feeling anxious, nervous, depressed, and stressed (Han et al. 2021, Li and Lyu 2020). However, the temporal direction of the relationship is unclear in these studies. Poorer mental health may precede risk perceptions (rather than vice versa). The authors of another cross-sectional study argued for this direction, reporting that anxiety and depression influence higher COVID-19 risk perceptions (Orte et al. 2020). However, longitudinal studies are required to better understand possible causal pathways.

Wellbeing is defined as the positive aspect of mental health; it is more than the absence of mental illness (Warwick Medical School 2020). To the best of our knowledge, previous studies have not examined associations between COVID-19 risk perceptions and wellbeing, specifically. Given its distinction from anxiety and depression, and the UK government’s recognition of wellbeing being critical to health policy (Department of Health and Social Care 2014), we think that there are insights to be gained by examining these constructs separately.

### Risk Behaviours

#### Smoking

Cross-sectional studies have found associations between COVID-19 risk perceptions and smoking behaviours, although the direction of the relationship is unclear. For example, Jackson and colleagues (2020) found that higher COVID-19 risk perceptions (stress about becoming seriously ill from COVID-19) were associated with smoking less than usual among smokers with post-16 qualifications. Higher COVID-19 risk perceptions (worries about catching COVID) were also associated with smoking *more* than usual, and these associations were stronger for smokers without post-16 qualifications than those with. Shepherd and colleagues (2021) found that COVID-19 worries (about contracting COVID-19, related symptoms, and associated health consequences) were positively associated with coping motives for smoking and perceived barriers for smoking cessation. Smokers also report lower adherence to COVID-19 prevention guidelines than never smokers, despite greater worries about infection (Jackson et al. 2020).

#### Electronic Cigarette Use

Electronic cigarettes (e-cigarettes), which can aid smoking cessation, are often used in conjunction with cigarettes (dual use) or as a replacement for cigarettes and are rarely used by people who have not smoked before (Hartmann-Boyce et al. 2021, Action on Smoking and Health 2020). Smoking and e-cigarette use should be considered separately because they may have different associations with COVID-19 risk perceptions. There is some research examining the associations between COVID-19 risk perceptions and e-cigarette use. For example, higher COVID-19 risk perceptions (beliefs that e-cigarette users are at greater risk from COVID-19 versus non-users) are associated with more frequent e-cigarette cessation considerations (Kelly, Pawson, and Vuolo 2020) and reductions in e-cigarette use (White et al. 2021). Furthermore, more frequent e-cigarette use was also associated with reduced beliefs that e-cigarette users are at greater risk from COVID-19 (Kelly, Pawson, and Vuolo 2020).

#### Alcohol Use

Cross-sectional studies suggest that there is a relationship between COVID-19 risk perceptions and alcohol use, and this relationship may depend on how COVID-19 risk perceptions are operationalised. For example, Panno and colleagues (2020) found an association between COVID-19 distress (an affective measure) and alcohol problems. Alpers and colleagues (2021) found that COVID-19 economic (not health) worries were associated with increased drinking. Furthermore, Garnett and colleagues (2021) found stress about catching COVID-19, becoming seriously ill, and financial stress were associated with drinking more than usual. However, the former was also associated with drinking less. Therefore, higher COVID-19 risk perceptions may motivate some people to reduce the amount they drink, smoke, or use e-cigarettes due to health concerns, and motivate others to drink, smoke, or use e-cigarettes more as a coping strategy (Yingst et al. 2021).

#### COVID-19 Transmission-Related Behaviours

Risk perceptions are central to protection motivation theory, which explains how protective behaviours are initiated and maintained (Rogers 1975, Floyd, Prentice-Dunn, and Rogers 2000). Higher COVID-19 risk perceptions (e.g., likelihood of infection) are associated with protective behaviours that reduce virus transmission, such as hand washing, social distancing, and wearing face coverings (Wise et al. 2020, Bruine de Bruin and Bennett 2020, Savadori and Lauriola 2020, Schneider et al. 2021, Dryhurst et al. 2020). Conversely, lower COVID-19 risk perceptions (perceived severity) are associated with riskier social behaviour during the pandemic (i.e., greater number of social contacts) (Wambua et al. 2022). It is therefore important to examine the associations between COVID-19 risk perceptions and social contact and self-isolating when infected, as these behaviours impact virus transmission (Atchison et al. 2021).

### Current Study

Previous research on this topic has predominantly been cross-sectional. Although some researchers have investigated longitudinal predictors of COVID-19 risk perceptions (Schneider et al. 2021), to the best of our knowledge no studies have examined the role of mental health (i.e., anxiety and depression), wellbeing, and substance use as predictors of COVID-19 risk perceptions. We were particularly interested in the question of whether poorer mental health and wellbeing may be causal risk factors for COVID-19 risk perceptions. Whilst observational data offer a relatively weak basis for causal inference, longitudinal (versus cross-sectional) data support somewhat stronger causal inference by providing clarity on the temporal relationship between exposures and outcomes (i.e., which comes first). In addition, polygenic risk scores (PRS) for anxiety, depression, and wellbeing (single scores that capture genetic liability to a trait or condition by combining multiple genetic variants) (Choi, Mak, and O’Reilly 2020) can also support stronger causal inference by reducing the potential for confounding variables. Because PRS are determined at conception and are stable over time, their association with an outcome should not be affected by confounders over the life course. By triangulating results from cross-sectional, longitudinal, and genetic studies, which have different limitations and sources of potential bias, we can build on insights from previous research (Lawlor, Tilling, and Davey Smith 2016). Consistency of findings from different approaches improves the reliability of the evidence (Lawlor, Tilling, and Davey Smith 2016, Hill 2015). Furthermore, stronger inferences regarding whether these associations reflect causal pathways would support risk communication.

We examined the bidirectional associations between COVID-19 risk perceptions and mental health, wellbeing, and risk behaviours using combined data from mothers and young people in the Avon Longitudinal Study of Parents and Children (ALSPAC), making our study one of the largest and most comprehensive studies on this topic. We included five risk perception variables, including those that were thought-related (‘cognitive’ e.g., likelihood of infection), feeling-related (‘affective’ e.g., worries about infection), self-related, other-related, and a holistic measure combining all items. These distinctions have not always been studied, but they matter as there are implications for pandemic risk communication. For example, if cognitive risk perceptions were most strongly related to negative outcomes, then public health messaging could focus on communicating more personalised risk information. If affective risk perceptions were most strongly related to negative outcomes, such risk communications could focus on reducing affective biases by providing appropriate context for the risk numbers being communicated, for example by making use of risk comparator information. This would help people to make meaning of the level of risk they are exposed to (Freeman et al. 2021).

First, we investigated cross-sectional associations between COVID-19 risk perceptions (exposures) and mental health (i.e., anxiety and depression), wellbeing, and risk behaviours (i.e., alcohol use, smoking, e-cigarette use, lack of self-isolating given a suspected COVID-19 infection, and face-to-face and physical contact outside the household) (outcomes). Cross-sectional data were used to answer this first question because longitudinal data were not available (i.e., risk perceptions were assessed in the most recent COVID-19 questionnaire, at the same time point as the outcomes).

Second, we investigated prospective longitudinal associations between pre-pandemic mental health (i.e., anxiety and depression), wellbeing, and risk behaviours (alcohol use, smoking, e-cigarette use) and early pandemic risk behaviours (lack of self-isolating, social contact) (exposures) and COVID-19 risk perceptions (outcomes). Third, we investigated whether genetic propensities for anxiety, depression, and wellbeing (exposures) are associated with COVID-19 risk perceptions (outcomes). As described above, we used longitudinal and genetic data here to expand on previous studies that have examined similar research questions with cross-sectional data, to triangulate findings.

We hypothesised that (1) COVID-19 risk perceptions would be positively associated with anxiety, depression, low wellbeing, alcohol use, and self-isolating, and negatively associated with social contact, (2) pre-pandemic anxiety, depression, low wellbeing and early pandemic self-isolating would be positively associated with COVID-19 risk perceptions, and pre-pandemic alcohol use and early pandemic social contact would be negatively associated with COVID-19 risk perceptions, and (3) anxiety and depression PRS and wellbeing PRS would be positively and negatively associated with COVID-19 risk perceptions, respectively. We had no directional hypotheses for smoking and e-cigarette use, given the mixed findings.

## Methods

### Design

We conducted cross-sectional and prospective longitudinal analyses of secondary data from ALSPAC, a UK population-based birth cohort study (Boyd et al. 2013, Fraser et al. 2013, Northstone et al. 2019). The sample was broadly representative of the region at the time (Boyd et al. 2013). Ethics approval was obtained from the ALSPAC Ethics and Law Committee and the Local Research Ethics Committees (http://www.bristol.ac.uk/alspac/researchers/research-ethics/). Informed consent for the use of data collected via questionnaires and clinics was obtained following recommendations of the ALSPAC Ethics and Law Committee. Consent for biological samples was collected in accordance with the Human Tissue Act (2004). Our study protocol was pre-registered on the Open Science Framework (https://osf.io/qan65/).

### Participants

ALSPAC recruited pregnant women living in Avon with expected delivery dates between April 1991-December 1992, and 14,541 pregnancies were initially enrolled. We used data from mothers (G0) and the original children (G1; ‘young people’) to maximise sample size. We could not include G0 partner data (mothers’ partners who were predominantly males), as identities cannot be linked across questionnaires. For example, a partner completing a prepandemic questionnaire may not be the same partner completing a pandemic questionnaire. Data from G1 participants at ≥22 years were collected and managed using REDCap (Harris et al. 2009). The ALSPAC study website contains the data dictionary and variable search tool (http://www.bristol.ac.uk/alspac/researchers/our-data).

### Polygenic Risk Scores

Summary statistics from genome-wide association studies (GWAS) for anxiety (Purves et al. 2020), depression (Howard et al. 2019), and wellbeing (Baselmans et al. 2019) were used to derive corresponding PRS among participants with genetic data. We calculated PRS using a threshold of *p* < .05 to increase the percentage of variance explained in each phenotype while trying to minimise pleiotropy. This increased our statistical power to detect an effect, given our sample size (relatively small for exploring genetic associations), but potentially at the expense of specificity. Genotype data were available for 8,196 mothers and 8,237 young people. Full details are available in the Supplementary Information.

### Self-Report Measures

The data dictionary describes all self-report measures (Supplementary Table S1). Variables were binary, except for the continuous COVID-19 risk perception variables that were used to test hypothesis 3. Time points of pre-pandemic measures (2003-2017) were selected based on the most recent and valid measures available (i.e., standardised scales preferred over single items). Therefore, follow-up periods varied from 3-17 (median 5) years (Supplementary Figure S1). Other studies using ALSPAC have used pre-pandemic measures from similar time points (Kwong et al. 2020). Separate variables were created for mothers, young people, and the whole sample combined, where possible.

#### Risk Perceptions

COVID-19 risk perceptions (five variables) were assessed in ALSPAC’s second COVID-19 questionnaire (26.05.2020 to 05.07.2020) (Northstone, Smith, et al. 2020). COVID-19 cognitive risk perceptions (i.e., thought-related risk perceptions) were measured by three summed items that assessed perceptions of COVID-19 impact, likelihood of infection, and severity of infection from 1 ‘strongly disagree’ to 5 ‘strongly agree’. COVID-19 affective risk perceptions (i.e., feeling-related risk perceptions) were measured by five summed items that assessed worries about COVID-19 infection (with respect to themselves [self] or other people [others]), transmission, and death (self/others) from 1 ‘not at all worried’ to 5 ‘very worried’. A holistic measure of COVID-19 risk perceptions was calculated by summing all eight items (mothers: Cronbach’s α = .82; young people: Cronbach’s α = .80). COVID-19 self-and other-risk perceptions combined items concerning oneself versus others, respectively. Binary variables were created by dichotomising continuous variables at the median. These binary variables were exposure variables for hypothesis 1, and outcome variables for hypothesis 2. The continuous variables were outcome variables for hypothesis 3.

#### Mental Health and Wellbeing

Outcomes: Current anxiety (generalised anxiety disorder; GAD) and depression (mental health variables), and wellbeing were assessed in the second COVID-19 questionnaire, using the Generalised Anxiety Disorder Assessment (GAD-7) (Spitzer et al. 2006), Short Mood and Feelings Questionnaire (SMFQ) (Angold et al. 1995), and Warwick-Edinburgh Mental Wellbeing Scale (WEMWBS) (Tennant et al. 2007), respectively. These measures have recommended binary cut-offs for examining the proportion of individuals with probable GAD (≥10) (Kroenke et al. 2007), likely depression (≥12) (Child Outcomes Research Consortium 2021, Jarbin et al. 2020), and low wellbeing (≤40) (Warwick Medical School 2021).

Exposures: Pre-pandemic anxiety, depression, and low wellbeing were assessed at different time points before the COVID-19 pandemic (2003-2017). For mothers, single items separately assessed pre-pandemic anxiety, depression, and low wellbeing (no/yes). For young people, pre-pandemic GAD and depression (mild episode) (no/yes) were derived from the Clinical Interview Schedule – Revised (CIS-R), and low wellbeing (no/yes) was derived from the WEMWBS.

#### Risk Behaviours

Outcomes: High-risk drinking (no/yes), increased alcohol use since lockdown (no/yes), increased smoking/e-cigarette use (no/yes), self-isolating given a suspected or confirmed COVID-19 infection (no/yes), and face-to-face and physical contact with individuals outside one’s household (none/at least one person) were assessed in the second COVID-19 questionnaire. The Alcohol Use Disorders Identification Test – Consumption (AUDIT-C) has a recommended cut-off for high-risk drinking (≥5) (Kelly et al. 2009).

Exposures: Pre-pandemic high-risk drinking, smoking (no/yes), and e-cigarette use (no/yes; young people only), were assessed at different time points (2012-2017). Early pandemic transmission-related behaviours (self-isolating, social contact) were assessed in the first COVID-19 questionnaire (09.04.2020 to 15.05.2020) (Northstone, Howarth, et al. 2020).

### Covariates

Age, sex, education, and keyworker status (partially adjusted models), and additionally, pre-pandemic anxiety, depression, high-risk drinking, smoking, and early pandemic suspected COVID-19 infection (fully adjusted models), were included as covariates. Covariates were selected based on their *a priori* relevance and/or their associations with risk perceptions, mental health, and/or risk behaviours in the literature (i.e., their potential to be a confounder). By using a categorical age variable (Supplementary Table S2), the age adjustment accounted for the bimodal age distribution.

### Statistical Analyses

Analyses were conducted in Stata SE (Version 15.0). We used logistic regression to examine cross-sectional and prospective longitudinal associations (hypotheses 1 and 2). We assessed the impact of potential confounding variables by comparing unadjusted and adjusted models. We planned to use multiple regression for hypothesis 2 and model all exposures simultaneously; however, to avoid reductions in sample size (due to pre-pandemic measures at different time points), we ran separate regressions for each exposure. We used linear regression for the PRS analyses (hypothesis 3) and adjusted for the top ten genetic principal components of ancestry (McVean 2009).

We analysed data from the whole sample (i.e., combining available data from mothers and young people), accounting for relatedness (i.e., by specifying that the standard errors allow for intragroup correlation, relaxing the independence of observations assumption). We also stratified analyses by generational cohort to explore differences. For example, older age is associated with higher risk perceptions of dying from COVID-19, but lower risk perceptions of being infected, and lower depression and anxiety (Bruine de Bruin and Bennett 2020). These stratified analyses were exploratory. We performed complete case analyses for hypotheses 1 and 2, to tease apart possible effects of confounding variables versus reductions in sample size between unadjusted and adjusted models. We report fully adjusted results for COVID-19 holistic risk perceptions unless stated otherwise. Results are interpreted in terms of the strength of evidence against the null hypothesis (e.g., *p* < .05 provides modest evidence whilst *p* < .001 provides strong evidence), direction of effect estimates, and consistency of evidence across sensitivity analyses (Sterne and Davey Smith 2001).

## Results

### Participant Characteristics

A total of 5,319 mothers and young people completed the second COVID-19 questionnaire, and 5,064 had complete data on COVID-19 risk perceptions. Sample sizes ranged from 413-5,115 for cross-sectional analyses, 233-4,243 for prospective longitudinal analyses, and 3,615-3,672 for PRS analyses. Age ranged from 27-29 years for young people *(M* = 27.7, *SD* = 0.6), and from 44-72 years for mothers (*M* = 58.1, *SD* = 4.4); 85% of the whole sample were female (71% of young people), and 98% were of a White ethnic group. Participant characteristics are summarised in Supplementary Tables S2-S7.

### Cross-Sectional Associations (Hypothesis 1)

#### Whole Sample

**Figure 1:**
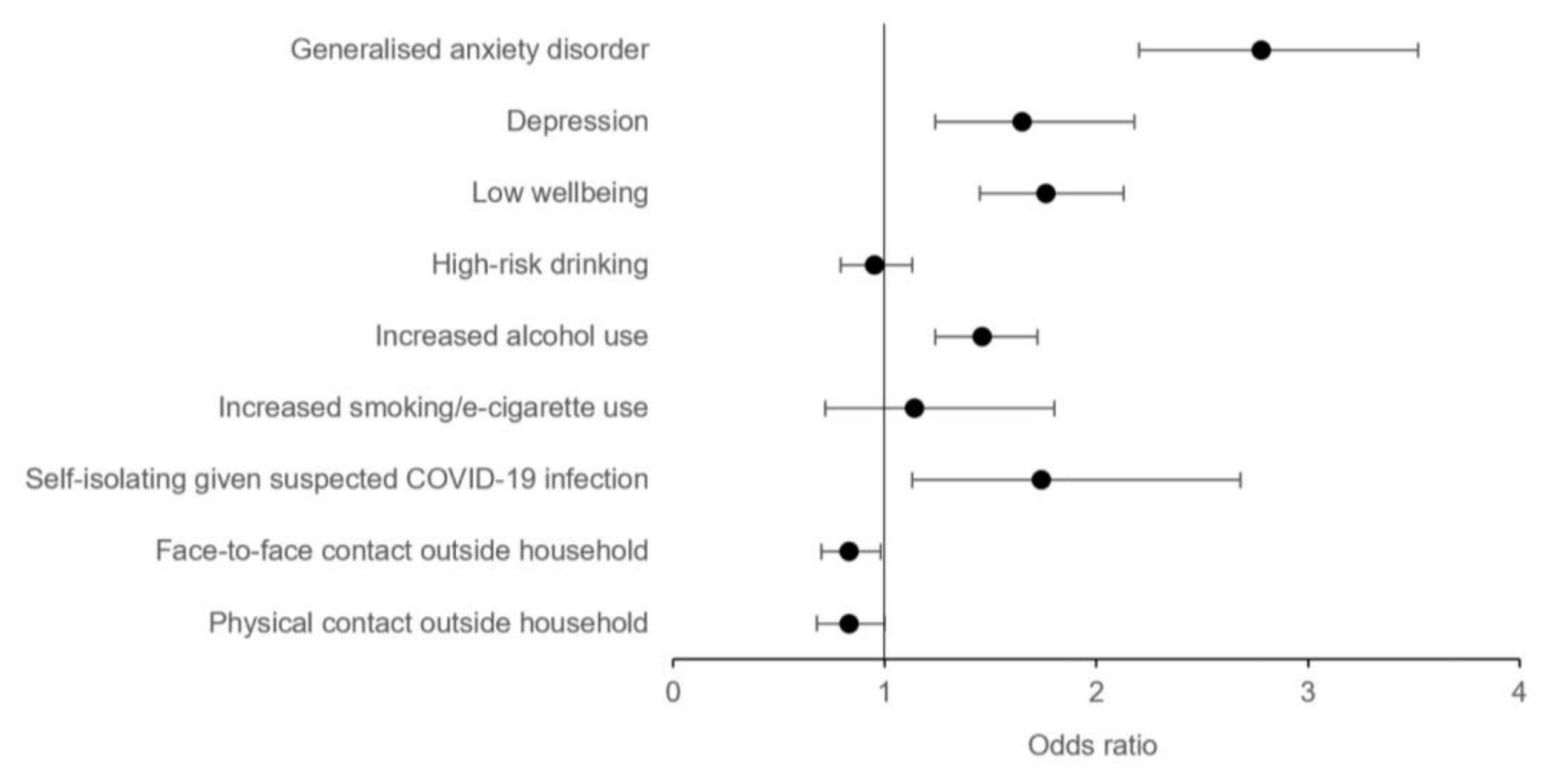
Cross-Sectional Associations between COVID-19 Holistic Risk Perceptions and Mental Health, Wellbeing, and Risk Behaviours *Note*. Whole sample. Forest plot shows the fully adjusted odds ratios (circles) and 95% confidence intervals (bars). Fully adjusted = adjusted for age, gender, education, keyworker status, pre-pandemic anxiety, depression, high-risk drinking, smoking, and early pandemic suspected COVID-19 infection.

Cross-sectional results are presented in Table 1 and Figure 1. There was strong evidence of a positive association between COVID-19 risk perceptions and GAD (OR 2.78, 95% confidence interval [CI] 2.20 to 3.52, *p* < .001), depression (OR 1.65, 95% CI 1.24 to 2.18, *p* < .001), and low wellbeing (OR 1.76, 95% CI 1.45 to 2.13, *p* < .001). Associations were consistent across risk perception dimensions, except cognitive, where associations with depression and low wellbeing were attenuated in fully adjusted models.

There was no clear evidence of an association between COVID-19 risk perceptions and high-risk drinking (OR 0.95, 95% CI 0.79 to 1.13, *p* = .54), or increased smoking/e-cigarette use (OR 1.14, 95% CI 0.72 to 1.80, *p* = .59). There was strong evidence that COVID-19 risk perceptions and increased alcohol use were positively associated (OR 1.46, 95% CI 1.24 to 1.72, *p* < .001), except for cognitive risk perceptions, which was not robust to adjustment for confounders. There were positive associations between some COVID-19 risk perceptions (holistic, cognitive) and self-isolating given a suspected COVID-19 infection (OR 1.74, 95% CI 1.13 to 2.68, *p* = .012). There were negative associations between some COVID-19 risk perceptions (holistic, affective, self) and face-to-face contact (OR 0.83, 95% CI 0.70 to 0.98, *p* = .027), and all COVID-19 risk perceptions and physical contact (OR 0.83, 95% CI 0.68 to 1.00, *p* = .049).

#### Sensitivity Analyses

Results stratified by cohort are presented in Supplementary Tables S8-S9. Results were largely similar across generations, except for increased alcohol use (positive associations for mothers only), and face-to-face contact (some negative associations for mothers only). Complete case results are presented in Supplementary Tables S10-S12. There were strong positive associations between COVID-19 risk perceptions (except cognitive) and GAD, depression, low wellbeing, and increased alcohol use (Table S10). Positive associations between some risk perceptions and self-isolating remained, as did negative associations between some risk perceptions and social contact.

### Prospective Longitudinal Associations (Hypothesis 2)

#### Whole Sample

**Figure 2:**
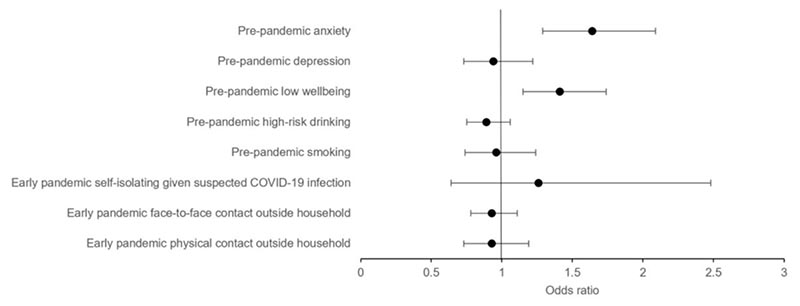
Longitudinal Associations between Pre-pandemic and Early Pandemic Variables and COVID-19 Holistic Risk Perceptions *Note.* Whole sample. Forest plot shows the fully adjusted odds ratios (circles) and 95% confidence intervals (bar). Fully adjusted = adjusted for age, gender, education, keyworker status, pre-pandemic anxiety, depression, high-risk drinking, smoking, and early pandemic suspected COVID-19 infection.

Results from prospective analyses with pre-pandemic measures are presented in Table 2 and Figure 2. There was strong evidence that pre-pandemic anxiety (OR 1.64, 95% CI 1.29 to 2.09, *p* < .001) and low wellbeing (OR 1.41, 95% CI 1.15 to 1.74, *p* = .001) were positively associated with COVID-19 risk perceptions, except cognitive. There was no clear evidence that pre-pandemic depression was associated with COVID-19 risk perceptions (OR 0.94, 95% CI 0.73 to 1.22, *p* = .65). Pre-pandemic high-risk drinking was negatively associated with COVID-19 self-risk perceptions only (OR 0.78, 95% CI 0.65 to 0.92, *p* = .004). There was no clear evidence that pre-pandemic smoking (OR 1.14, 95% CI 0.72 to 1.80, *p* = .59) or e-cigarette use (OR 1.49, 95% CI 0.72 to 3.09, *p* = .29; Supplementary Table S13) were associated with COVID-19 risk perceptions.

Results from prospective analyses with early pandemic measures are presented in Table 3 and Figure 2. There was no clear evidence that early pandemic self-isolating given a suspected COVID-19 infection (OR 1.26, 95% CI 0.64 to 2.48, *p* = .50), face-to-face contact (OR 0.93, 95% CI 0.78 to 1.11, *p* = .43), or physical contact (OR 0.93, 95% CI 0.73 to 1.19, *p* = .56) were associated with later COVID-19 risk perceptions.

#### Sensitivity Analyses

Results stratified by cohort are presented in Supplementary Tables S13-S16. Results were largely similar across generations, except for pre-pandemic high-risk drinking (negative associations with self-risk perceptions for young people only) and smoking (positive associations with self-risk perceptions for mothers only). Results from the complete case analyses are presented in Supplementary Tables S17-S22. Positive associations between pre-pandemic anxiety and low wellbeing and COVID-19 risk perceptions remained, and the negative association between pre-pandemic high-risk drinking and COVID-19 self-risk perceptions remained (Supplementary Table S17).

### Polygenic Risk Score Associations (Hypothesis 3)

There was no clear evidence that the anxiety PRS was associated with COVID-19 risk perceptions (*b* 0.12, 95% CI -0.08 to 0.31, *p* = .24). The depression PRS was positively associated with COVID-19 holistic, affective, and other-risk perceptions (*b* 0.21, 95% CI 0.02 to 0.40, *p* = .029), whilst the wellbeing PRS was negatively associated with COVID-19 risk perceptions (except cognitive) (*b* -0.29, 95% CI -0.48 to -0.09, *p* = .004). PRS results are shown in Table 4.

### Attrition

*Post hoc* analyses to explore differential attrition revealed that the anxiety and depression PRS were negatively associated with completion of the first COVID-19 questionnaire (OR 0.92, 95% CI 0.89 to 0.96, *p* < .001; OR 0.93, 95% CI 0.90 to 0.97, *p* < .001, respectively) and the second COVID-19 questionnaire (OR 0.95, 95% CI 0.92 to 0.99, *p* = .02; OR 0.95, 95% CI 0.91 to 0.98, *p* = .006, respectively). The wellbeing PRS was positively associated with completion of the first (OR 1.12, 95% CI 1.07 to 1.16, *p* < .001) and second (OR 1.10, 95% CI 1.05 to 1.14, *p* < .001) COVID-19 questionnaires.

## Discussion

In support of hypothesis 1, higher COVID-19 risk perceptions (except cognitive) were cross-sectionally associated with higher anxiety, depression, lower wellbeing, and increased alcohol use. For some risk perception measures, higher COVID-19 risk perceptions were associated with self-isolating given a suspected COVID-19 infection, and less social contact. Our findings support studies that have found associations between higher COVID-19 risk perceptions and worse mental health (Han et al. 2021, Li and Lyu 2020, Yin et al. 2021, Zhong et al. 2021), drinking more than usual (Garnett et al. 2021), and increased COVID-19 prevention behaviours (Dryhurst et al. 2020, Schneider et al. 2021). COVID-19 risk perceptions were not associated with high-risk drinking or increased smoking/e-cigarette use.

In support of hypothesis 2, pre-pandemic anxiety and low wellbeing were associated with higher COVID-19 risk perceptions (except cognitive), indicating a temporal relationship consistent with a causal effect of anxiety and wellbeing on later risk perceptions. However, pre-pandemic depression was only associated with higher COVID-19 risk perceptions in the unadjusted analyses, and there was no clear evidence of an association in the adjusted analyses (which included pre-pandemic anxiety as a covariate). Anxiety and depression are frequently comorbid (Lamers et al. 2011), therefore, comorbid anxiety may have been driving the unadjusted associations for pre-pandemic depression. Pre-pandemic high-risk drinking was associated with lower COVID-19 self-risk perceptions. Pre-pandemic smoking and e-cigarette use, and early pandemic self-isolating and social contact were not associated with COVID-19 risk perceptions. These analyses with longitudinal data extend previous findings with cross-sectional data, by helping to determine the temporal direction of associations.

There were differences between COVID-19 risk perception dimensions. Mental health and wellbeing were associated with affective (not cognitive) dimensions, perhaps unsurprisingly as worries are a common feature across anxiety disorders and depression (Rabner et al. 2017). Pre-pandemic anxiety was also more strongly associated with COVID-19 worries than pre-pandemic depression, a distinction supported elsewhere (Wright, Steptoe, and Fancourt 2021). Cognitive models of anxiety and depression suggest that anxiety is future oriented and predictive of threat, whereas depression is past oriented (Dobson 1985), which may explain these differences. Odds of increased alcohol use (measure excluded nondrinkers) were higher among individuals with higher risk perceptions, suggesting a possible drinking to cope mechanism.

In support of hypothesis 3, the wellbeing PRS was negatively associated with COVID-19 risk perceptions (except cognitive), and the depression PRS was positively associated with COVID-19 risk perceptions (except cognitive and self). However, there was no clear evidence of an association for the anxiety PRS. This could be due to limited statistical power; the anxiety PRS was the weakest genetic instrument and explained less variance in the phenotype compared to the depression and wellbeing PRS. Stronger instruments could be created as larger GWAS of more precisely measured phenotypes become available. Furthermore, cohorts with larger samples than ALSPAC would have more power to detect genetic associations. The lack of clear statistical evidence for self-reported pre-pandemic depression (versus depression PRS) may be due to measurement differences. The self-report measure represented participants who reported a mild depressive episode, whereas the genome-wide meta-analysis of depression included individuals reporting clinical diagnoses of, and meeting standard criteria for, major depressive disorder. Furthermore, given that anxiety and depression are frequently comorbid (Lamers et al. 2011), there may have been statistical overadjustment in models where the other was included as a covariate. Despite some limitations, this is the first study to have used PRS data to understand the relationship between pre-pandemic mental health and wellbeing and COVID-19 risk perceptions. Again, these analyses extend previous findings by helping to support stronger causal inference by reducing the potential for confounding variables.

Results were largely similar across generational cohorts, although exploratory analyses suggested some differences across age groups. First, among mothers, COVID-19 risk perceptions and increased alcohol use were cross-sectionally positively associated, but we did not see evidence of this among young people. This is consistent with evidence of increased alcohol consumption among older (versus younger) individuals during the pandemic (Sallie et al. 2020), and drinking to cope is common among older adults (Gilson, Bryant, and Judd 2017). However, differences may have been driven by biological sex, because older participants were mothers (i.e., categorised as females). For example, women are more likely than men to drink to cope (Peltier et al. 2019). Second, some negative associations between COVID-19 risk perceptions and face-to-face contact only held in mothers, which may be explained by age/employment differences; 20% of mothers were retired, potentially making reduction of social contact easier. Third, pre-pandemic high-risk drinking was negatively associated with COVID-19 self-risk perceptions in young people only. It is plausible that people who engage in any risky behaviours perceive lower risks to themselves generally. But this association may not have held in older adults, who may be aware of the disproportionate negative effects of COVID-19 on their health (Mueller, McNamara, and Sinclair 2020). Finally, pre-pandemic smoking was positively associated with COVID-19 self-risk perceptions in mothers only, again possibly due to age-related risk. Stratified analyses were exploratory; future studies could test the robustness of these findings, which should be considered hypothesis-generating, in other samples.

Our study has limitations. First, the sample was predominantly female and of a White ethnic group, which may impact the generalisability of results. Males report lower COVID-19 risk perceptions (Rodriguez-Besteiro et al. 2021, Dryhurst et al. 2020). However, we did adjust for biological sex, and we also presented results separately for mothers and young people, with the latter cohort having a greater proportion of males than in the combined cohort. Furthermore, people from Black, Asian, and Minority Ethnic communities are nearly twice as likely to die from COVID-19 than people of a White ethnic group (White and Ayoubkhani 2020). Therefore, ethnicity may influence COVID-19 risk perceptions. Second, we combined two generational cohorts, which resulted in a bimodal age distribution. However, we adjusted for age and additionally we conducted analyses stratified by generational cohort. Third, we used pandemic data from one time point, which cannot capture changes as a pandemic evolves (Zhong et al. 2021, Brown, Coventry, and Pepper 2021). Changes in policies, vaccine development, knowledge, and personal experiences may influence risk perceptions and behaviours. Longitudinal studies with repeated assessments during and after pandemics are required to examine bidirectionality. Fourth, we adjusted for suspected COVID-19 infection because this is associated with lower risk perceptions and higher risk behaviours (Smith et al. 2020), however we could not include COVID-19 severity (hospital admission), which likely influences risk perceptions, due to participant disclosure risk. Therefore, there may be unmeasured confounding variables. Fifth, risk perception is a heterogeneous construct, and there is no standardised measure (Lanciano et al. 2020). Future studies should also include work/economic and social/relationship risk perceptions to reflect the pervasive impact of a pandemic. For example, work/economic COVID-19 risk perceptions are reportedly higher than those concerning health (Lanciano et al. 2020), and increased drinking is more frequent among people reporting economic (versus health) COVID-19 worries (Alpers et al. 2021). Sixth, there was evidence of differential attrition; people at a higher risk of anxiety and depression were less likely to have completed the COVID-19 questionnaires. The properties of these missing individuals remain unknown, and hence the bias is difficult to predict. However, this pattern of attrition may have attenuated our associations (e.g., for the anxiety PRS) towards the null (i.e., the true associations may be stronger than reported). Finally, smoking and e-cigarette use were conflated in the COVID-19 questionnaires but should be examined separately. Smokers with higher COVID-19 risk perceptions could have switched to using e-cigarettes, but this would not have been captured in the data.

Our study also has strengths. First, longitudinal data helped to determine the temporal direction of associations, extending findings from previous cross-sectional studies, although cause and effect cannot be established in observational studies. Second, we adjusted analyses for various potential covariates, to reduce the chance of reverse causation and confounding bias. Third, the large sample (albeit relatively small for exploring genetic associations) increased the power to detect associations in the observational analyses. Fourth, we conducted extensive complete case analyses to help tease apart the influence of sample size reductions and potential confounding variables. Fifth, we explored differences between thought-related, feeling-related, self-related, and other-related COVID-19 risk perceptions, which has not been examined previously and has implications for risk communication. Finally, genetic analyses were consistent with the possibility that low wellbeing and depression may play a causal role in COVID-19 risk perceptions, building on insights from previous research that only used self-report data. Although this research question was causal, and we used the best data and methods available to us to answer this, inferences must be cautious. Mendelian Randomization (MR) analyses in larger samples are needed to test the causality question fully. Genetic variants can be used in MR analyses to provide (under certain assumptions) unconfounded causal estimates (Davey Smith and Ebrahim 2003). MR typically uses single-nucleotide polymorphisms (SNPs) that reach genome-wide significance (i.e., *p*<5×10^-8^) (Richardson et al. 2019). PRS can be derived using more liberal *p*-value thresholds, which capture more genetic variance but can reduce the specificity of the PRS to the exposure of interest (e.g., by including more variants with pleiotropic effects).

COVID-19 risk perceptions were associated with poorer mental health, lower wellbeing, and increased alcohol use, and pre-pandemic anxiety and low wellbeing increased COVID-19 risk perceptions. This is concerning, given the increase in alcohol-related deaths in 2020 (Holmes and Angus 2021), and because worries about adversities can be as detrimental for mental health as actually experiencing adversities (Wright, Steptoe, and Fancourt 2021). However, some risk perceptions were also associated with COVID-19 transmission-related behaviours. A balanced approach to risk communication and public health messaging, in the context of the current pandemic and during future pandemics, is therefore required. As well as promoting public awareness of pandemic-related physical health risks to maintain rational risk perceptions and adherence to government guidelines, political and public health officials must also promote mental health and wellbeing for example by providing reassurance, adaptive coping strategies, and remote interventions to help people manage their worries (Zhong et al. 2021, Orte et al. 2020, Han et al. 2021, Bruine de Bruin and Bennett 2020). COVID-19 will be prevalent for years to come, with many scientists predicting that the virus that causes COVID-19 (SARS-CoV-2) will become endemic (Phillips 2021, Li et al. 2020). Furthermore, these findings about the interplay between COVID-19 risk perceptions, mental health, wellbeing, and risk behaviours will be valuable for future pandemics, informing broader pandemic preparedness efforts.

### Conclusions

Higher COVID-19 risk perceptions were associated with anxiety, depression, low wellbeing, increased alcohol use, and COVID-19 transmission-related behaviours. Prepandemic anxiety and low wellbeing were associated with higher COVID-19 risk perceptions, and pre-pandemic high-risk drinking was associated with lower COVID-19 risk perceptions regarding oneself. Associations were most robust for anxiety and low wellbeing given the consistency across risk perception dimensions (except cognitive), cross-sectional and prospective analyses, and complete case analyses. Genetic analyses were consistent with the possibility that low wellbeing and depression may play a causal role in COVID-19 risk perceptions, but formal MR analyses in larger samples are warranted. This study offers a novel contribution to the field because of its use of longitudinal and genetic data, inclusion of different components of COVID-19 risk perceptions, and relatively large sample size. These findings have implications for the understanding and management of COVID-19 in the long-term, and of future pandemics.

## Supplementary Material

Supplementary Information

## Figures and Tables

**Table 1:** Cross-Sectional Associations between COVID-19 Risk Perceptions and Mental Health, Wellbeing, and Risk Behaviours (Whole Sample)

COVID-19 Risk Perceptions (Exposures)															
	Holistic			Cognitive			Affective			Self			Other		
Outcome and Model	OR (95% CI)	P	N	OR (95% CI)	P	N	OR (95% CI)	P	N	OR (95% CI)	P	N	OR (95% CI)	P	N
**Generalised Anxiety Disorder**															
Unadjusted	2.75 (2.35, 3.21)	<.001	4982	1.60 (1.38, 1.86)	<.001	5065	2.74 (2.34, 3.22)	<.001	4994	2.16 (1.86, 2.52)	<.001	5038	3.00 (2.55, 3.53)	<.001	5011
Partially Adjusted	3.05 (2.55, 3.66)	<.001	4244	1.52 (1.28, 1.80)	<.001	4314	3.12 (2.58, 3.77)	<.001	4255	2.78 (2.32, 3.33)	<.001	4289	2.97 (2.46, 3.58)	<.001	4270
Fully Adjusted	2.78 (2.20, 3.52)	<.001	2780	1.50 (1.19, 1.89)	.001	2820	2.70 (2.12, 3.44)	<.001	2787	2.63 (2.08, 3.32)	<.001	2806	2.71 (2.12, 3.45)	<.001	2796
**Depression**															
Unadjusted	1.81 (1.52, 2.16)	<.001	4922	1.42 (1.20, 1.69)	<.001	5006	1.81 (1.51, 2.16)	<.001	4933	1.45 (1.22, 1.72)	<.001	4977	2.03 (1.70, 2.43)	<.001	4951
Partially Adjusted	1.90 (1.55, 2.34)	<.001	4198	1.23 (1.00, 1.50)	.048	4268	1.94 (1.57, 2.41)	<.001	4208	1.83 (1.49, 2.26)	<.001	4242	2.02 (1.63, 2.49)	<.001	4224
Fully Adjusted	1.65 (1.24, 2.18)	<.001	2753	1.12 (0.85, 1.47)	.430	2793	1.77 (1.33, 2.36)	<.001	2760	1.57 (1.19, 2.09)	.002	2778	1.92 (1.44, 2.56)	<.001	2770
**Low Wellbeing**															
Unadjusted	1.84 (1.62, 2.09)	<.001	4942	1.32 (1.16, 1.50)	<.001	5025	1.84 (1.62, 2.10)	<.001	4954	1.57 (1.38, 1.78)	<.001	4997	1.77 (1.56, 2.01)	<.001	4972
Partially Adjusted	1.91 (1.65, 2.21)	<.001	4213	1.29 (1.11, 1.49)	.001	4283	1.94 (1.67, 2.26)	<.001	4224	1.82 (1.57, 2.12)	<.001	4257	1.74 (1.50, 2.01)	<.001	4240
Fully Adjusted	1.76 (1.45, 2.13)	<.001	2766	1.18 (0.97, 1.43)	.090	2806	1.83 (1.50, 2.22)	<.001	2773	1.79 (1.48, 2.17)	<.001	2791	1.56 (1.29, 1.89)	<.001	2783
**High-Risk Drinking**															
Unadjusted	0.81 (0.72, 0.91)	<.001	5022	1.14 (1.01, 1.27)	.027	5107	0.76 (0.67, 0.85)	<.001	5034	0.75 (0.67, 0.84)	<.001	5077	0.91 (0.81, 1.02)	.095	5051
Partially Adjusted	0.88 (0.78, 1.01)	.061	4287	1.09 (0.97, 1.24)	.154	4358	0.84 (0.74, 0.96)	.009	4297	0.87 (0.77, 0.99)	.039	4331	0.95 (0.84, 1.08)	.458	4313
Fully Adjusted	0.95 (0.79, 1.13)	.537	2809	1.18 (0.99, 1.40)	.059	2849	0.89 (0.74, 1.06)	.188	2816	0.98 (0.82, 1.18)	.860	2834	0.97 (0.82, 1.15)	.726	2826
**Increased Alcohol Use**															
Unadjusted	1.31 (1.16, 1.48)	<.001	4334	1.11 (0.98, 1.25)	.092	4405	1.31 (1.16, 1.47)	<.001	4343	1.22 (1.09, 1.38)	.001	4379	1.22 (1.09, 1.38)	.001	4357
Partially Adjusted	1.40 (1.23, 1.60)	<.001	3698	1.13 (0.99, 1.29)	.069	3758	1.39 (1.22, 1.59)	<.001	3704	1.31 (1.15, 1.50)	<.001	3734	1.28 (1.12, 1.46)	<.001	3719
Fully Adjusted	1.46 (1.24, 1.72)	<.001	2541	1.13 (0.96, 1.32)	.140	2578	1.52 (1.29, 1.79)	<.001	2544	1.39 (1.18, 1.64)	<.001	2561	1.29 (1.10, 1.52)	.001	2556
**Increased Smoking/E-Cigarette Use**															
Unadjusted	1.41 (1.08, 1.82)	.010	941	1.13 (0.87, 1.46)	.369	963	1.30 (1.00, 1.68)	.050	942	1.14 (0.88, 1.48)	.307	957	1.47 (1.13, 1.90)	.004	946
Partially Adjusted	1.45 (1.06, 1.98)	.019	726	1.21 (0.89, 1.63)	.225	741	1.26 (0.92, 1.73)	.146	726	1.24 (0.90, 1.69)	.188	736	1.45 (1.07, 1.97)	.017	730
Fully Adjusted	1.14 (0.72, 1.80)	.586	420	0.97 (0.63, 1.49)	.888	426	0.98 (0.63, 1.54)	.942	420	1.00 (0.64, 1.57)	.989	422	1.31 (0.85, 2.03)	.222	423
**Self-Isolating Given Suspected COVID-19 Infection**															
Unadjusted	1.40 (1.04, 1.90)	.028	758	2.24 (1.65, 3.05)	<.001	777	1.09 (0.80, 1.47)	.589	761	0.83 (0.61, 1.12)	.218	765	1.35 (1.00, 1.81)	.047	769
Partially Adjusted	1.60 (1.14, 2.26)	.007	638	2.40 (1.70, 3.37)	<.001	655	1.27 (0.90, 1.78)	.177	640	0.92 (0.65, 1.30)	.634	643	1.40 (1.01, 1.95)	.044	649
Fully Adjusted	1.74 (1.13, 2.68)	.012	413	2.27 (1.48, 3.48)	<.001	422	1.30 (0.85, 2.01)	.231	415	0.98 (0.63, 1.54)	.943	416	1.42 (0.94, 2.16)	.096	421
**Face-To-Face Contact Outside Household**															
Unadjusted	0.86 (0.77, 0.97)	.011	5029	0.86 (0.77, 0.97)	.010	5115	0.86 (0.77, 0.97)	.012	5042	1.00 (0.90, 1.13)	.946	5085	0.86 (0.77, 0.96)	.010	5059
Partially Adjusted	0.77 (0.67, 0.88)	<.001	4293	0.91 (0.80, 1.04)	.160	4365	0.74 (0.65, 0.85)	<.001	4304	0.83 (0.72, 0.94)	.005	4338	0.83 (0.73, 0.95)	.006	4320
Fully Adjusted	0.83 (0.70, 0.98)	.027	2816	0.91 (0.77, 1.08)	.280	2857	0.78 (0.66, 0.92)	.004	2823	0.82 (0.69, 0.97)	.019	2842	0.88 (0.75, 1.05)	.149	2833
**Physical Contact Outside Household**															
Unadjusted	0.84 (0.74, 0.97)	.016	4733	0.79 (0.69, 0.91)	.001	4812	0.84 (0.74, 0.96)	.013	4744	0.84 (0.73, 0.96)	.011	4785	0.82 (0.71, 0.94)	.004	4761
Partially Adjusted	0.85 (0.73, 0.98)	.030	4046	0.82 (0.71, 0.95)	.008	4112	0.83 (0.72, 0.97)	.017	4056	0.81 (0.70, 0.94)	.007	4087	0.83 (0.71, 0.96)	.012	4072
Fully Adjusted	0.83 (0.68, 1.00)	.049	2662	0.78 (0.65, 0.94)	.008	2700	0.79 (0.65, 0.95)	.013	2668	0.71 (0.60, 0.86)	<.001	2685	0.84 (0.69, 1.01)	.057	2678

*Note.* Logistic regressions. OR = odds ratio. CI = confidence interval. Partially adjusted = adjusted for sociodemographic variables (age, gender, education, and keyworker status). Fully adjusted = additionally adjusted for prior mental health and risk behaviour variables (anxiety, depression, high-risk drinking, smoking, and suspected COVID-19 infection). All variables in the models are binary. All risk perception variables were dichotomised at the median.

**Table 2:** Longitudinal Associations between Pre-pandemic Mental Health, Wellbeing, and Risk Behaviours and COVID-19 Risk Perceptions (Whole Sample)

COVID-19 Risk Perceptions (Outcomes)															
	Holistic			Cognitive			Affective			Self			Other		
Exposure and Model	OR (95% CI)	P	N	OR (95% CI)	P	N	OR (95% CI)	P	N	OR (95% CI)	P	N	OR (95% CI)	P	N
**Pre-pandemic Anxiety**															
Unadjusted	1.52 (1.30, 1.78)	<.001	4165	1.09 (0.93, 1.28)	.276	4235	1.65 (1.41, 1.93)	<.001	4175	1.52 (1.30, 1.78)	<.001	4207	1.41 (1.20, 1.65)	<.001	4192
Partially Adjusted	1.47 (1.23, 1.75)	<.001	3673	1.24 (1.04, 1.47)	.014	3733	1.54 (1.29, 1.84)	<.001	3682	1.25 (1.05, 1.49)	.012	3709	1.50 (1.27, 1.79)	<.001	3697
Fully Adjusted	1.64 (1.29, 2.09)	<.001	2533	1.34 (1.06, 1.71)	.016	2570	1.74 (1.37, 2.22)	<.001	2539	1.44 (1.13, 1.84)	.003	2557	1.75 (1.37, 2.22)	<.001	2547
**Pre-pandemic Depression**															
Unadjusted	1.30 (1.11, 1.53)	.002	4174	1.02 (0.87, 1.20)	.815	4243	1.34 (1.14, 1.58)	<.001	4184	1.21 (1.03, 1.42)	.022	4216	1.22 (1.04, 1.44)	.016	4201
Partially Adjusted	1.17 (0.98, 1.41)	.084	3680	1.10 (0.92, 1.31)	.294	3740	1.23 (1.02, 1.47)	.026	3689	1.02 (0.85, 1.23)	.808	3716	1.18 (0.99, 1.41)	.069	3705
Fully Adjusted	0.94 (0.73, 1.22)	.648	2533	0.98 (0.77, 1.26)	.903	2570	0.95 (0.74, 1.23)	.716	2539	0.86 (0.66, 1.12)	.263	2557	0.88 (0.68, 1.14)	.341	2547
**Pre-pandemic Low Wellbeing**															
Unadjusted	1.51 (1.30, 1.75)	<.001	4056	1.19 (1.03, 1.38)	.019	4125	1.58 (1.36, 1.84)	<.001	4067	1.55 (1.34, 1.80)	<.001	4102	1.45 (1.25, 1.69)	<.001	4080
Partially Adjusted	1.47 (1.25, 1.74)	<.001	3597	1.24 (1.06, 1.45)	.008	3657	1.52 (1.29, 1.80)	<.001	3606	1.40 (1.19, 1.65)	<.001	3636	1.46 (1.24, 1.72)	<.001	3619
Fully Adjusted	1.41 (1.15, 1.74)	.001	2465	1.19 (0.97, 1.46)	.101	2502	1.52 (1.24, 1.87)	<.001	2471	1.53 (1.24, 1.89)	<.001	2489	1.34 (1.09, 1.65)	.005	2479
**Pre-pandemic High-Risk Drinking**															
Unadjusted	0.79 (0.69, 0.90)	<.001	3738	1.06 (0.93, 1.20)	.388	3796	0.73 (0.64, 0.84)	<.001	3748	0.63 (0.56, 0.72)	<.001	3777	0.93 (0.82, 1.06)	.290	3760
Partially Adjusted	0.90 (0.76, 1.04)	.144	3332	0.94 (0.81, 1.08)	.371	3382	0.88 (0.76, 1.02)	.079	3341	0.80 (0.69, 0.93)	.003	3365	0.98 (0.85, 1.13)	.757	3352
Fully Adjusted	0.89 (0.75, 1.06)	.192	2533	0.97 (0.82, 1.14)	.702	2570	0.89 (0.74, 1.05)	.169	2539	0.78 (0.65, 0.92)	.004	2557	1.02 (0.86, 1.21)	.807	2547
**Pre-pandemic Smoking**															
Unadjusted	1.05 (0.89, 1.24)	.573	4135	1.06 (0.90, 1.26)	.485	4198	1.04 (0.87, 1.23)	.687	4145	0.93 (0.78, 1.10)	.384	4175	1.28 (1.08, 1.52)	.005	4159
Partially Adjusted	1.11 (0.91, 1.35)	.308	3670	1.01 (0.83, 1.22)	.957	3726	1.10 (0.91, 1.34)	.332	3679	1.14 (0.94, 1.38)	.186	3705	1.22 (1.00, 1.48)	.048	3692
Fully Adjusted	0.96 (0.74, 1.24)	.755	2533	1.01 (0.78, 1.29)	.966	2570	0.94 (0.73, 1.22)	.667	2539	1.08 (0.84, 1.40)	.529	2557	1.12 (0.86, 1.45)	.394	2547

*Note.* Logistic regressions. OR = odds ratio. CI = confidence interval. Partially adjusted = adjusted for sociodemographic variables (age, gender, education, and keyworker status). Fully adjusted = additionally adjusted for prior mental health and risk behaviour variables (pre-pandemic anxiety, depression, high-risk drinking, smoking, and early pandemic suspected COVID-19 infection). The same sociodemographic variables are included in all partially adjusted models. However, the variables in the fully adjusted models differ based on the exposure in each model (e.g., pre-pandemic anxiety is removed as a confounder when pre-pandemic anxiety is the exposure). All variables in the models are binary. All risk perception variables were dichotomised at the median.

**Table 3:** Longitudinal Associations between Early Pandemic Risk Behaviours and COVID-19 Risk Perceptions (Whole Sample)

COVID-19 Risk Perceptions (Outcomes)															
	Holistic			Cognitive			Affective			Self			Other		
Exposure and Model	OR (95% CI)	P	N	OR (95% CI)	P	N	OR (95% CI)	P	N	OR (95% CI)	P	N	OR (95% CI)	P	N
**Early Pandemic Self-Isolating Given Suspected COVID-19 Infection**															
Unadjusted	1.45 (0.88, 2.37)	.142	394	1.27 (0.79, 2.05)	.323	410	1.57 (0.96, 2.57)	.073	395	1.29 (0.79, 2.11)	.304	400	1.33 (0.82, 2.15)	.245	401
Partially Adjusted	1.11 (0.63, 1.94)	.724	352	1.41 (0.82, 2.44)	.212	366	1.20 (0.68, 2.10)	.534	353	0.92 (0.52, 1.62)	.766	357	1.05 (0.60, 1.82)	.868	359
Fully Adjusted	1.26 (0.64, 2.48)	.500	245	1.53 (0.81, 2.87)	.188	253	1.44 (0.73, 2.86)	.295	233	0.83 (0.42, 1.64)	.587	248	1.31 (0.69, 2.47)	.413	251
**Early Pandemic Face-To-Face Contact Outside Household**															
Unadjusted	0.95 (0.84, 1.08)	.430	4056	0.80 (0.71, 0.91)	.001	4123	0.97 (0.86, 1.10)	.629	4067	1.01 (0.89, 1.14)	.890	4097	0.91 (0.80, 1.03)	.133	4081
Partially Adjusted	0.89 (0.77, 1.02)	.096	3545	0.83 (0.72, 0.95)	.008	3604	0.92 (0.80, 1.06)	.270	3553	0.90 (0.78, 1.04)	.151	3581	0.93 (0.81, 1.07)	.289	3567
Fully Adjusted	0.93 (0.78, 1.11)	.434	2411	0.88 (0.75, 1.05)	.155	2446	0.98 (0.82, 1.16)	.805	2415	0.92 (0.77, 1.10)	.355	2432	1.05 (0.88, 1.24)	.609	2425
**Early Pandemic Physical Contact Outside Household**															
Unadjusted	0.86 (0.72, 1.03)	.103	3619	0.82 (0.68, 0.98)	.026	3674	0.93 (0.78, 1.11)	.411	3628	0.93 (0.78, 1.11)	.439	3653	0.89 (0.75, 1.07)	.220	3641
Partially Adjusted	0.89 (0.73, 1.08)	.243	3166	0.81 (0.66, 0.98)	.030	3213	0.97 (0.80, 1.18)	.775	3174	0.98 (0.81, 1.19)	.835	3195	0.92 (0.76, 1.12)	.401	3185
Fully Adjusted	0.93 (0.73, 1.19)	.563	2159	0.91 (0.71, 1.15)	.418	2189	0.94 (0.74, 1.19)	.596	2163	1.01 (0.79, 1.28)	.965	2177	1.08 (0.85, 1.37)	.539	2172

*Note.* Logistic regressions. OR = odds ratio. CI = confidence interval. Partially adjusted = adjusted for sociodemographic variables (age, gender, education, and keyworker status). Fully adjusted = additionally adjusted for prior mental health and risk behaviour variables (pre-pandemic anxiety, depression, high-risk drinking, smoking, and early pandemic suspected COVID-19 infection). All variables in the models are binary. All risk perception variables were dichotomised at the median.

**Table 4:** Prospective Longitudinal Associations between Mental Health and Wellbeing Polygenic Risk Scores and COVID-19 Risk Perceptions

COVID-19 Risk Perceptions (Outcomes)															
	Holistic			Cognitive			Affective			Self			Other		
Exposure and Model	*b* (95% CI)	P	N	*b* (95% CI)	P	N	*b* (95% CI)	P	N	*b* (95% CI)	P	N	*b* (95% CI)	P	N
**Whole Sample**															
**Anxiety**															
Unadjusted	0.12 (-0.07, 0.32)	.206	3615	0.03 (-0.03, 0.09)	.407	3672	0.09 (-0.08, 0.25)	.308	3623	0.04 (-0.06, 0.13)	.430	3652	0.08 (-0.03, 0.20)	.166	3633
Fully Adjusted	0.12 (-0.08, 0.31)	.236	3615	0.02 (-0.04, 0.08)	.465	3672	0.08 (-0.08, 0.25)	.331	3623	0.03 (-0.06, 0.13)	.471	3652	0.08 (-0.04, 0.20)	.193	3633
**Depression**															
Unadjusted	0.22 (0.04, 0.41)	.018	3615	0.02 (-0.04, 0.08)	.578	3672	0.20 (0.04, 0.36)	.015	3623	0.08 (-0.01, 0.17)	.077	3652	0.14 (0.03, 0.26)	.014	3633
Fully Adjusted	0.21 (0.02, 0.40)	.029	3615	0.01 (-0.05, 0.07)	.663	3672	0.19 (0.03, 0.35)	.023	3623	0.06 (-0.03, 0.15)	.171	3652	0.15 (0.03, 0.26)	.013	3633
**Wellbeing**															
Unadjusted	-0.29 (-0.49, -0.10)	.003	3615	-0.02 (-0.08, 0.04)	.501	3672	-0.27 (-0.44, -0.10)	.001	3623	-0.12 (-0.21, -0.03)	.007	3652	-0.17 (-0.29, -0.05)	.006	3633
Fully Adjusted	-0.29 (-0.48, -0.09)	.004	3615	-0.01 (-0.08, 0.05)	.630	3672	-0.27 (-0.44, -0.10)	.002	3623	-0.11 (-0.21, -0.02)	.013	3652	-0.17 (-0.29, -0.05)	.006	3633
**Mother Sample**															
**Anxiety**															
Unadjusted	0.11 (-0.16, 0.38)	.426	1792	-0.01 (-0.09, 0.07)	.838	1824	0.12 (-0.12, 0.35)	.334	1796	0.05 (-0.07, 0.18)	.400	1806	0.06 (-0.11, 0.22)	.513	1807
Fully Adjusted	0.11 (-0.17, 0.38)	.444	1792	-0.01 (-0.09, 0.07)	.810	1824	0.12 (-0.12, 0.35)	.338	1796	0.05 (-0.08, 0.18)	.444	1806	0.06 (-0.11, 0.22)	.507	1807
**Depression**															
Unadjusted	0.26 (-0.01, 0.53)	.057	1792	0.04 (-0.04, 0.12)	.366	1824	0.23 (-0.00, 0.46)	.054	1796	0.10 (-0.03, 0.22)	.134	1806	0.17 (0.01, 0.34)	.037	1807
Fully Adjusted	0.26 (-0.02, 0.53)	.067	1792	0.03 (-0.05, 0.11)	.495	1824	0.24 (-0.00, 0.47)	.052	1796	0.08 (-0.05, 0.21)	.209	1806	0.19 (0.02, 0.36)	.027	1807
**Wellbeing**															
Unadjusted	-0.33 (-0.60, -0.05)	.020	1792	-0.03 (-0.11, 0.05)	.470	1824	-0.31 (-0.54, -0.07)	.011	1796	-0.14 (-0.27, -0.01)	.032	1806	-0.19 (-0.36, -0.02)	.026	1807
Fully Adjusted	-0.32 (-0.60, -0.03)	.030	1792	-0.02 (-0.11, 0.07)	.675	1824	-0.31 (-0.56, -0.06)	.014	1796	-0.13 (-0.26, 0.01)	.065	1806	-0.20 (-0.37, -0.02)	.027	1807
**Young Person Sample**															
**Anxiety**															
Unadjusted	0.18 (-0.07, 0.42)	.158	1823	0.05 (-0.04, 0.13)	.274	1848	0.11 (-0.10, 0.32)	.305	1827	0.06 (-0.05, 0.18)	.293	1846	0.11 (-0.05, 0.26)	.178	1826
Fully Adjusted	0.15 (-0.10, 0.40)	.252	1823	0.04 (-0.04, 0.13)	.318	1848	0.08 (-0.13, 0.29)	.455	1827	0.05 (-0.07, 0.17)	.386	1846	0.09 (-0.07, 0.25)	.283	1826
**Depression**															
Unadjusted	0.20 (-0.05, 0.46)	.113	1823	-0.01 (-0.09, 0.07)	.812	1848	0.19 (-0.02, 0.41)	.079	1827	0.08 (-0.04, 0.19)	.213	1846	0.11 (-0.05, 0.27)	.173	1826
Fully Adjusted	0.16 (-0.10, 0.42)	.218	1823	-0.02 (-0.10, 0.07)	.706	1848	0.16 (-0.06, 0.38)	.162	1827	0.06 (-0.07, 0.18)	.364	1846	0.09 (-0.07, 0.25)	.285	1826
**Wellbeing**															
Unadjusted	-0.29 (-0.54, -0.04)	.025	1823	-0.00 (-0.08, 0.08)	.979	1848	-0.27 (-0.48, -0.06)	.013	1827	-0.13 (-0.25, -0.02)	.025	1846	-0.14 (-0.30, 0.02)	.077	1826
Fully Adjusted	-0.26 (-0.53, -0.00)	.048	1823	0.00 (-0.09, 0.09)	.993	1848	-0.24 (-0.47, -0.02)	.031	1827	-0.12 (-0.25, -0.00)	.049	1846	-0.13 (-0.30, 0.03)	.121	1826

*Note.* Linear regressions. *b* = unstandardised beta coefficient. CI = confidence interval. Polygenic risk scores were created using the p-value threshold of 0.05. Fully adjusted models = adjusted for genetic principal components of ancestry (PC1-PC10).

## Data Availability

The analysis code is available from the University of Bristol’s Research Data Repository (http://data.bris.ac.uk/data/), DOI: (https://data.bris.ac.uk/data/dataset/34bmhh800n6pb25aeva7qucjre). GWAS summary statistics used to create the PRS are available from the original publications. The informed consent obtained from ALSPAC participants does not allow the data to be made freely available through any third party maintained public repository. However, data used for this submission can be made available on request to the ALSPAC Executive. The ALSPAC data management plan describes in detail the policy regarding data sharing, which is through a system of managed open access. Full instructions for applying for data access can be found here: http://www.bristol.ac.uk/alspac/researchers/access/. The ALSPAC study website contains details of all the data that are available (http://www.bristol.ac.uk/alspac/researchers/our-data/).
