# Supplementary material for: Associations between COVID-19 Risk Perceptions and Mental Health, Wellbeing, and Risk Behaviours
Source: J Risk Res. Author manuscript; Available in PMC 2023 Oct 23. (PMC7615237; doi:10.1080/13669877.2022.2127849)
Supplement: Supplementary Information [file EMS189628-supplement-Supplementary_Information.docx]

**Supplementary Information**

***Genotyping and Imputation***

The Avon Longitudinal Study of Parents and Children (ALSPAC) children were genotyped using the Illumina HumanHap550 quad chip genotyping platforms by 23andme subcontracting the Wellcome Trust Sanger Institute, Cambridge, UK and the Laboratory Corporation of America, Burlington, NC, US. The resulting raw genome-wide data were subjected to standard quality control methods. Individuals were excluded on the basis of gender mismatches, minimal or excessive heterozygosity, disproportionate levels of individual missingness (>3%) and insufficient sample replication (identity by descent [IBD] <0.8). Population stratification was assessed by multidimensional scaling analysis and compared with Hapmap II (release 22) European descent (CEU), Han Chinese, Japanese and Yoruba reference populations; all individuals with non-European ancestry were removed. Single nucleotide polymorphisms (SNPs) with a minor allele frequency of <1%, a call rate of <95% or evidence for violations of Hardy-Weinberg equilibrium (*p* <5E-7) were removed. Cryptic relatedness was measured as proportion of identity by descent (IBD >0.1). Related subjects that passed all other quality control thresholds were retained during subsequent phasing and imputation. 9,115 subjects and 500,527 SNPs passed these quality control filters.

ALSPAC mothers were genotyped using the Illumina human660W-quad array at Centre National de Génotypage (CNG) and genotypes were called with Illumina GenomeStudio. PLINK (v1.07) was used to carry out quality control measures on an initial set of 10,015 subjects and 557,124 directly genotyped SNPs. SNPs were removed if they displayed more than 5% missingness or a Hardy-Weinberg equilibrium (P <1.0e-06). Additionally, SNPs with a minor allele frequency of <1% were removed. Samples were excluded if they displayed more than 5% missingness, had indeterminate X chromosome heterozygosity or extreme autosomal heterozygosity. Samples showing evidence of population stratification were identified by multidimensional scaling of genome-wide identity by state pairwise distances using the four HapMap populations as a reference, and then excluded. Cryptic relatedness was assessed using a IBD estimate of more than 0.125 which is expected to correspond to roughly 12.5% alleles shared IBD or a relatedness at the first cousin level. Related subjects that passed all other quality control thresholds were retained during subsequent phasing and imputation. 9,048 subjects and 526,688 SNPs passed these quality control filters.

We combined 477,482 SNP genotypes in common between the sample of mothers and sample of children. We removed SNPs with genotype missingness above 1% due to poor quality (11,396 SNPs removed) and removed a further 321 subjects due to potential ID mismatches. This resulted in a dataset of 17,842 subjects containing 6,305 duos and 465,740 SNPs (112 were removed during liftover and 234 were out of HWE after combination). We estimated haplotypes using ShapeIT (v2.r644) which utilises relatedness during phasing. The phased haplotypes were then imputed to the Haplotype Reference Consortium (HRCr1.1, 2016) panel of approximately 31,000 phased whole genomes. The HRC panel was phased using ShapeIt v2, and the imputation was performed using the Michigan imputation server. This gave 8,237 eligible children and 8,196 eligible mothers with available genotype data after exclusion of related subjects using cryptic relatedness measures described previously.

***Polygenic Risk Scores***

Polygenic risk scores (PRS) were calculated with PRSice 2⁠ using the default option of clumping correlated SNPs within a 250kb window at an R^2^ threshold of 0.1. The PRS were standardised prior to analysis (*M* = 0; *SD* = 1). All independent SNPs associated at *p* < .05 were included in the PRS. SNPs with a minor allele frequency below 1% or imputation quality (R^2^) below 0.80 were excluded. Weighted PRS were calculated based on the number of copies of each risk allele carried by an individual (this ranged from 0 to 2 for each SNP) and multiplied by the effect estimate identified in the discovery GWAS; these were then summed to create a continuous PRS for each phenotype.

**Supplementary Table S1**

*Data Dictionary for the Self-Report Variables*

| **Variable** | **Variable Description** |
| --- | --- |
| **COVID-19 Risk Perception Variables** | |
| COVID-19 Cognitive  Risk Perceptions | 3 items scored on a 5-point scale from 1 ‘strongly disagree’ to 5 ’strongly agree’.  Item 1: ‘The coronavirus/COVID-19 will not affect very many people in the country I’m currently living in’ (reverse scored); item 2: ‘I will probably get sick with the coronavirus/COVID-19’; item 3: ‘Getting sick with the coronavirus/COVID-19 can be serious’.  Continuous variable: total ranging from 3-15.  Binary variable: 0 = ‘low’ 1 = ‘high’ based on the median of the continuous variable.  Timepoint: 26/May/2020 – 5/Jul/2020 (second COVID-19 questionnaire).  Same for G0, G1, and Whole Sample cohorts. |
| COVID-19 Affective  Risk Perceptions | 5 items scored on a 5-point scale from 1 ‘not at all worried’ to 5 ‘very worried’.  Item 4: ‘Getting COVID-19’; item 5: ‘Someone close to me getting COVID-19’; item 6: ‘Passing on COVID-19 to others (even if I don’t know I have it);’ item 7: ‘Dying as a result of becoming infected with COVID-19’; item 8: ‘Someone close to me dying as a result of becoming infected with COVID-19’.  Continuous variable: total ranging from 5-25.  Binary variable: 0 = ‘low’ 1 = ‘high’ based on the median of the continuous variable.  Timepoint: 26/May/2020 – 5/Jul/2020 (second COVID-19 questionnaire).  Same for G0, G1, and Whole Sample cohorts. |
| COVID-19 Self  Risk Perceptions | Continuous variable: sum of item 2 (‘I will probably get sick with the coronavirus/COVID-19’), item 4 (‘worried about getting COVID-19’), and item 7 (‘worried about dying as a result of becoming infected with COVID-19’), and total ranging from 3-15.  Binary variable: 0 = ‘low’ 1 = ‘high’ based on the median of the continuous variable.  Timepoint: 26/May/2020 – 5/Jul/2020 (second COVID-19 questionnaire).  Same for G0, G1, and Whole Sample cohorts. |
| COVID-19 Other  Risk Perceptions | Continuous variable: sum of item 1 (‘the coronavirus/COVID-19 will not affect very many people in the country I’m currently living in’ [reverse scored]), item 3 (‘getting sick with the coronavirus/COVID-19 can be serious’), item 5 (‘worried about someone close to me getting COVID-19’), item 6 (‘worried about passing on COVID-19 to others (even if I don’t know I have it))’, and item 8 (‘worried about someone close to me dying as a result of becoming infected with COVID-19’), and total ranging from 5-25.  Binary variable: 0 = ‘low’ 1 = ‘high’ based on the median of the continuous variable.  Timepoint: 26/May/2020 – 5/Jul/2020 (second COVID-19 questionnaire).  Same for G0, G1, and Whole Sample cohorts. |
| COVID-19 Holistic  Risk Perceptions | Continuous variable: sum of items 1 to 8 (i.e., the three cognitive items and the five affective items), and total ranging from 8-40.  Binary variable: 0 = ‘low’ 1 = ‘high’ based on the median of the continuous variable.  Timepoint: 26/May/2020 – 5/Jul/2020 (second COVID-19 questionnaire).  Same for G0, G1, and Whole Sample cohorts. |
| **Sociodemographic Variables** | |
| Age | G0: Binary variable: 0 = ‘<60 years old’ 1 = ‘≥60 years old’. Dichotomised at 60 due to COVID risk.  G1: Continuous variable: ranging from 27-29 years old.  Whole Sample: Categorical variable: 0 = ‘all young people’ 1 = ‘mums <60 years old’ 2 = ‘mums ≥60 years old’.  Timepoint: 26/May/2020 – 5/Jul/2020 |
| Gender | Binary variable: 0 = ‘female’ 1 = ‘male’.  Same for G1 and Whole Sample cohorts. Not applicable for G0. |
| Ethnic Group | Binary variable: 0 = ‘White’ 1 = ‘non-White’. The ethnic origins were obtained using the format asked in the 1991 United Kingdom Census. This categorises the person as White, Black/Caribbean, Black/African, Black/other, Indian, Pakistani, Bangladeshi, Chinese, Other Specified. The variable was dichotomised in this way as ‘White’ was the largest subgroup.  Same for G0, G1, and Whole Sample cohorts. |
| Education | 0 = ‘did not study at university’ 1 = ‘studied at university’.  Same for G0, G1, and Whole Sample cohorts.  G0 Timepoint: 4/Mar/1991 - 4/Jan/1993 (at 32 weeks gestation).  G1 Timepoint: Nov 2018 - May 2019 (when young person was 26 years old). |
| Key Worker | 0 = ‘no’ 1 = ‘yes’.  Same for G0, G1, and Whole Sample cohorts.  Timepoint: 26/May/2020 – 5/Jul/2020 (second COVID-19 questionnaire). |
| Health/Social Care Worker | 0 = ‘no’ 1 = ‘yes’.  Same for G0, G1, and Whole Sample cohorts.  Timepoint: 26/May/2020 – 5/Jul/2020 (second COVID-19 questionnaire). |
| **Current Health Variables (During Pandemic)** | |
| Generalised Anxiety Disorder (GAD) | Binary variable for probable generalised anxiety disorder based on scores on the Generalised Anxiety Disorder Assessment (GAD-7).  Scores 0-9 = no/mild anxiety, 10-21 = moderate/severe anxiety.  Generalised Anxiety: 0 = ‘no’ 1 = ‘yes’.  Same for G0, G1, and Whole Sample cohorts.  Timepoint: 26/May/2020 – 5/Jul/2020 (second COVID-19 questionnaire). |
| Depression | Binary variable for likely depression based on scores on the Short Mood and Feelings Questionnaire (SMFQ).  Scores 0-11 = not likely depression, 12-26 = likely depression.  Depression: 0 = ‘no’ 1 = ‘yes’.  Same for G0, G1, and Whole Sample cohorts.  Timepoint: 26/May/2020 – 5/Jul/2020 (second COVID-19 questionnaire). |
| Low Wellbeing | Binary variable for low mental wellbeing based on scores on the Warwick-Edinburgh Mental Wellbeing Scale (WEMWBS).  Scores 14-40 = low mental wellbeing, 41-70 = high mental wellbeing.  Low Wellbeing: 0 = ‘no’ 1 = ‘yes’.  Same for G0, G1, and Whole Sample cohorts.  Timepoint: 26/May/2020 – 5/Jul/2020 (second COVID-19 questionnaire). |
| Medical Condition | Binary variable for presence of a medical condition based on 11 conditions reported: Diabetes, heart disease/heart problems, hypertension, overweight, kidney disease, liver disease, anaemia, asthma, other lung condition, cancer, and condition affecting brain/nerves.  Medical Condition: 0 = ‘no’ 1 = ‘yes’.  Same for G0, G1, and Whole Sample cohorts, except cancer removed for G1 and whole sample, due to low cell counts (disclosure risk).  Timepoint: 19/Apr/2020 – 15/May/2020 (first COVID-19 questionnaire). |
| Self-Isolating as Vulnerable | Binary variable: 0 ‘not self-isolating or not self-isolating for being in a vulnerable group’ 1 ‘self-isolating because in a vulnerable group’.  Same for G0, G1, and Whole Sample cohorts.  Timepoint: 26/May/2020 – 5/Jul/2020 (second COVID-19 questionnaire). |
| Living with a Vulnerable Person | Binary variable: 0 ‘no, does live with someone elderly or shielding’ 1 ‘yes, does not live with someone elderly or shielding’.  Same for G0, G1, and Whole Sample cohorts.  Timepoint: 26/May/2020 – 5/Jul/2020 (second COVID-19 questionnaire). |
| Suspected COVID-19 (Self-Report) | Binary variable for confirmed (test)/suspected (by doctor/self) COVID.  Suspected COVID-19 (Self-Report): 0 = ‘no’ 1 = ‘yes’.  Same for G0, G1, and Whole Sample cohorts.  Timepoint: 26/May/2020 – 5/Jul/2020 (second COVID-19 questionnaire; when confounder in the cross-sectional analyses), and 19/Apr/2020 – 15/May/2020 (first COVID-19 questionnaire; when confounder in the prospective longitudinal analyses). |
| Suspected COVID-19 (Algorithm) | Binary variable for predicted case of COVID-19 based on Menni algorithm of reported symptoms.  Suspected COVID-19 (Algorithm): 0 = ‘no’ 1 = ‘yes’.  Same for G0, G1, and Whole Sample cohorts.  Timepoint: 26/May/2020 – 5/Jul/2020 (second COVID-19 questionnaire). |
| **Pre-pandemic Health** | |
| Anxiety | G0: Binary variable for whether mother had anxiety or nerves in the last 2 years (regardless of if they consulted a doctor).  Timepoint: 14/May/2003 - 5/May/2005 (when young person was 12 years old).  G1: Binary variable for whether young person had generalised anxiety disorder, derived from the Clinical Interview Schedule – Revised (CIS-R).  Timepoint: June 2015 - Oct 2017 (when young person was 24 years old).  Whole Sample: combination of measures.  Anxiety: 0 = ‘no’ 1 = ‘yes’. |
| Depression | G0: Binary variable for whether mother had depression in the last 2 years (regardless of if they consulted a doctor).  Timepoint: 14/May/2003 - 5/May/2005 (when young person was 12 years old).  G1: Binary variable for whether young person had a mild depressive episode, derived from the CIS-R.  Timepoint: June 2015 - Oct 2017 (when young person was 24 years old).  Whole Sample: combination of measures.  Depression: 0 = ‘no’ 1 = ‘yes’. |
| Low Wellbeing | G0: Binary variable for whether mother has been a happy person in the past 2 weeks.  Low Wellbeing: 0 = ‘no’ 1 = ‘yes’.  Timepoint: Sep/2012 - Dec/2014 (when young person was 22 years old).  G1: Binary variable for low mental wellbeing based on scores on the WEMWBS.  Scores 14-40 = low mental wellbeing, 41-70 = high mental wellbeing.  Low Wellbeing: 0 = ‘no’ 1 = ‘yes’.  Timepoint: Dec/2015 - Sep/2016 (when young person was 23 years old).  Whole Sample: combination of measures.  Low Wellbeing: 0 = ‘no’ 1 = ‘yes’. |
| **Current Risk Behaviours** | |
| High-Risk Drinking | Binary variable for high-risk drinking based on scores on the Alcohol Use Disorders Identification Test – Consumption (AUDIT-C).  3 items measure frequency of use, frequency of binge drinking, and amount of alcohol.  Scores 0-4 = lower risk drinking, 5-12 = higher risk drinking.  High-Risk Drinking: 0 = ‘no’ 1 = ‘yes’.  Same for G0, G1, and Whole Sample cohorts.  Timepoint: 26/May/2020 – 5/Jul/2020 (second COVID-19 questionnaire). |
| Increased Alcohol Use | Binary variable for whether amount of alcohol participant drinks has changed since lockdown.  Increased Alcohol Use: 0 ‘decreased or stayed the same’ 1 ‘Increased’.  Same for G0, G1, and Whole Sample cohorts.  Timepoint: 26/May/2020 – 5/Jul/2020 (second COVID-19 questionnaire). |
| Increased Smoking/E-Cigarette Use | Binary variable for whether amount participant smokes/vapes has changed since lockdown.  Increased Smoking/Vaping: 0 = ‘decreased or stayed the same’ 1 = ‘increased’.  Same for G0, G1, and Whole Sample cohorts.  Timepoint: 26/May/2020 – 5/Jul/2020 (second COVID-19 questionnaire). |
| Self-isolating given a suspected COVID-19 infection | Binary variable based on whether people self-isolated given a confirmed or suspected COVID-19 infection.  Self-Isolation Given COVID-19: 0 = ‘no’ 1 = ‘yes’.  Same for G0, G1, and Whole Sample cohorts.  Timepoint: 26/May/2020 – 5/Jul/2020 (second COVID-19 questionnaire). |
| Face-to-face contact with people outside of household | Binary variable based on how many people participant spoke to yesterday (face-to-face) who were outside their household.  Face-To-Face Contact: 0 = ‘no-one’ 1 = ‘at least one person’.  Same for G0, G1, and Whole Sample cohorts.  Timepoint: 26/May/2020 – 5/Jul/2020 (second COVID-19 questionnaire). |
| Physical contact with people outside of household | Binary variable based on how many people participant spoke to yesterday (with physical contact) who were outside their household.  Physical Contact: 0 = ‘no-one’ 1 = ‘at least one person’.  Same for G0, G1, and Whole Sample cohorts.  Timepoint: 26/May/2020 – 5/Jul/2020 (second COVID-19 questionnaire). |
| **Pre-pandemic Risk Behaviours** | |
| High-Risk Drinking | Binary variable for high-risk drinking based on scores on the (AUDIT-C).  Scores 0-4 = lower risk drinking, 5-12 = higher risk drinking.  High-Risk Drinking: 0 = ‘no’ 1 = ‘yes’.  Same for G0, G1, and Whole Sample cohorts.  G0 Timepoint: Sep/2012 - Dec/2014 (when young person was 22 years old).  G1 Timepoint: Nov/2014 - Oct/2015 (when young person was 22 years old). |
| Smoking | Binary variable based on whether participant is currently a smoker (cigarettes or tobacco).  Smoking: 0 = ‘no’ 1 = ‘yes’.  Same for G0, G1, and Whole Sample cohorts.  G0 Timepoint: Sep/2012 - Dec/2014 (when young person was 22 years old).  G1 Timepoint: Nov/2016 - Aug/2017 (when young person was 24 years old). |
| E-cigarette use | Binary variable based on young person currently uses/vapes e-cigarettes/other vaping devices.  E-cigarette use: 0 = ‘no’ 1 = ‘yes’.  No e-cigarette use variable for G0 and thus no variable for whole sample.  G1 Timepoint: Nov/2016 - Aug/2017 (when young person was 24 years old). |
| **Early Pandemic Risk Behaviours** | |
| Self-isolating given a suspected COVID-19 infection | Binary variable based on whether people self-isolated given a confirmed or suspected COVID-19 infection.  Self-Isolation Given COVID-19: 0 = ‘no’ 1 = ‘yes’.  Same for G0, G1, and Whole Sample cohorts.  Timepoint: 19/Apr/2020 – 15/May/2020 (first COVID-19 questionnaire). |
| Face-to-face contact with people outside of household | Binary variable based on how many people participant spoke to yesterday (face-to-face) who were outside their household.  Face-To-Face Contact: 0 = ‘no-one’ 1 = ‘at least one person’.  Same for G0, G1, and Whole Sample cohorts.  Timepoint: 19/Apr/2020 – 15/May/2020 (first COVID-19 questionnaire). |
| Physical contact with people outside of household | Binary variable based on how many people participant spoke to yesterday (with physical contact) who were outside their household.  Physical Contact: 0 = ‘no-one’ 1 = ‘at least one person’.  Same for G0, G1, and Whole Sample cohorts.  Timepoint: 19/Apr/2020 – 15/May/2020 (first COVID-19 questionnaire). |

*Note.* G0 = mothers, G1 = young people, whole sample = G0 and G1 cohorts combined.

**Supplementary Figure S1**

*Timeline of Pre-Pandemic (Light Grey) and Pandemic (Dark Grey) Self-Reported Variables for Mothers (G0) and Young People (G1)*


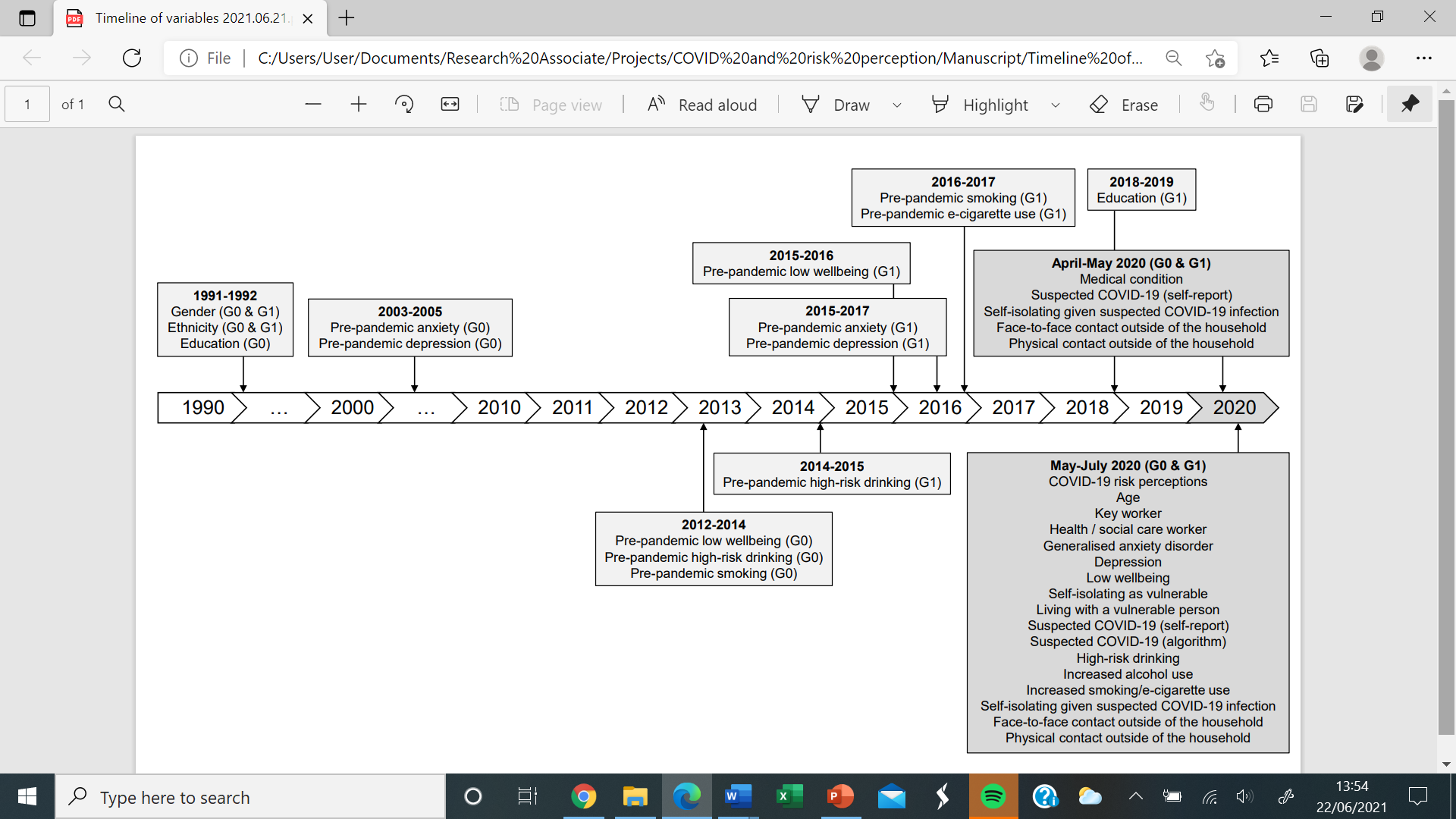


**Supplementary Table S2**

*Frequencies and Percentages of COVID-19 Risk Perceptions by Sociodemographic, Health, and Risk Behaviour Variables (Whole Sample)*

|  | **COVID-19 Risk Perceptions (High Only)** | | | | | | | | | | | |
| --- | --- | --- | --- | --- | --- | --- | --- | --- | --- | --- | --- | --- |
|  | **Complete Sample** | | **Holistic** | | **Cognitive** | | **Affective** | | **Self** | | **Other** | |
| **Participant Characteristic** | **N** | **%** | **N** | **%** | **N** | **%** | **N** | **%** | **N** | **%** | **N** | **%** |
| **Complete Sample** | - | - | 2332 | 46.1 | 2467 | 47.9 | 2588 | 51.0 | 2493 | 48.7 | 2588 | 50.8 |
| **Sociodemographic** | | | | | | | | | | | | |
| 27-30 Years of Age (Young People) | 2710 | 51.0 | 1081 | 42.3 | 1350 | 52.0 | 1178 | 45.9 | 1016 | 39.2 | 1333 | 52.0 |
| <60 Years of Age (Mothers) | 1649 | 31.0 | 813 | 51.1 | 703 | 43.5 | 918 | 57.6 | 939 | 58.7 | 822 | 51.2 |
| ≥60 Years of Age (Mothers) | 960 | 18.1 | 438 | 47.8 | 414 | 44.2 | 492 | 53.5 | 538 | 58.0 | 433 | 46.9 |
| Female | 4521 | 85.1 | 2129 | 49.4 | 2114 | 48.1 | 2360 | 54.6 | 2293 | 52.6 | 2302 | 53.0 |
| Male | 795 | 15.0 | 202 | 27.0 | 352 | 46.6 | 227 | 30.2 | 199 | 26.3 | 285 | 38.1 |
| White Ethnicity | 4787 | 97.5 | 2090 | 45.8 | 2238 | 48.2 | 2312 | 50.6 | 2241 | 48.6 | 2315 | 50.5 |
| Non-White Ethnicity | 122 | 2.5 | 57 | 47.9 | 49 | 41.2 | 65 | 54.6 | 52 | 43.7 | 69 | 58.0 |
| No University Education | 2497 | 53.2 | 1228 | 51.7 | 1025 | 42.2 | 1389 | 58.3 | 1382 | 57.5 | 1240 | 51.8 |
| University Education | 2201 | 46.9 | 830 | 39.1 | 1147 | 53.5 | 901 | 42.4 | 837 | 39.1 | 1033 | 48.5 |
| Not a Key Worker | 3144 | 63.3 | 1383 | 44.8 | 1485 | 47.4 | 1537 | 49.7 | 1490 | 47.8 | 1540 | 49.7 |
| Key Worker | 1820 | 36.7 | 855 | 48.0 | 891 | 49.0 | 947 | 53.0 | 905 | 50.2 | 939 | 52.3 |
| Not a Health/Social Care Worker | 4314 | 84.6 | 1922 | 45.5 | 2047 | 47.6 | 2130 | 50.2 | 2062 | 48.3 | 2127 | 50.0 |
| Health/Social Care Worker | 788 | 15.4 | 375 | 48.5 | 394 | 50.1 | 421 | 54.3 | 396 | 50.6 | 423 | 54.4 |
| **Current Health** | | | | | | | | | | | | |
| No Probable Generalised Anxiety | 4223 | 83.1 | 1736 | 42.0 | 1938 | 46.1 | 1948 | 47.0 | 1906 | 45.6 | 1938 | 46.6 |
| Probable Generalised Anxiety | 858 | 16.9 | 563 | 66.6 | 495 | 57.8 | 600 | 70.8 | 550 | 64.4 | 614 | 72.3 |
| No Likely Depression | 4407 | 87.8 | 1912 | 44.2 | 2062 | 46.9 | 2127 | 49.1 | 2078 | 47.6 | 2118 | 48.7 |
| Likely Depression | 613 | 12.2 | 354 | 59.0 | 340 | 55.7 | 382 | 63.6 | 345 | 56.8 | 397 | 65.8 |
| No Low Mental Wellbeing | 3759 | 74.6 | 1555 | 42.1 | 1739 | 46.4 | 1740 | 47.0 | 1704 | 45.7 | 1756 | 47.3 |
| Low Mental Wellbeing | 1281 | 25.4 | 717 | 57.3 | 680 | 53.3 | 779 | 62.1 | 722 | 56.9 | 774 | 61.4 |
| No Medical Conditions | 2426 | 58.3 | 937 | 40.2 | 1130 | 47.8 | 1039 | 44.4 | 964 | 41.0 | 1116 | 47.7 |
| ≥1 Medical Conditions | 1738 | 41.7 | 868 | 52.1 | 827 | 48.8 | 970 | 58.1 | 948 | 56.3 | 914 | 54.4 |
| Not Self-Isolating as Not Vulnerable | 4902 | 95.0 | 2120 | 44.6 | 2291 | 47.4 | 2362 | 49.5 | 2261 | 47.0 | 2384 | 49.8 |
| Self-Isolating as Vulnerable | 256 | 5.0 | 176 | 71.0 | 145 | 57.5 | 184 | 73.9 | 193 | 76.6 | 165 | 66.3 |
| Not Live with Vulnerable Person | 4564 | 90.0 | 2022 | 45.2 | 2199 | 48.3 | 2246 | 50.1 | 2166 | 47.9 | 2255 | 50.1 |
| Live with Vulnerable Person | 506 | 10.0 | 259 | 52.2 | 226 | 44.8 | 288 | 57.8 | 275 | 54.8 | 280 | 56.1 |
| No Suspected COVID-19 (Self-Report) | 4504 | 84.8 | 1987 | 46.4 | 2027 | 46.6 | 2218 | 51.6 | 2167 | 50.0 | 2169 | 50.4 |
| Suspected COVID-19 (Self-Report) | 806 | 15.2 | 342 | 44.5 | 438 | 55.6 | 367 | 47.6 | 324 | 41.8 | 416 | 53.4 |
| No Suspected COVID-19 (Algorithm) | 5159 | 97.0 | 2253 | 45.9 | 2382 | 47.7 | 2499 | 50.7 | 2415 | 48.6 | 2499 | 50.6 |
| Suspected COVID-19 (Algorithm) | 159 | 3.0 | 78 | 52.4 | 85 | 55.9 | 88 | 58.7 | 78 | 51.7 | 88 | 58.3 |
| **Pre-pandemic Health** | | | | | | | | | | | | |
| No Anxiety | 3556 | 81.8 | 1490 | 43.7 | 1642 | 47.4 | 1637 | 48.0 | 1606 | 46.6 | 1677 | 48.9 |
| Anxiety | 792 | 18.2 | 409 | 54.1 | 381 | 49.5 | 459 | 60.3 | 434 | 57.0 | 439 | 57.4 |
| No Depression | 3640 | 83.6 | 1548 | 44.4 | 1693 | 47.7 | 1706 | 48.9 | 1682 | 47.7 | 1739 | 49.6 |
| Depression | 716 | 16.4 | 352 | 51.0 | 335 | 48.2 | 389 | 56.2 | 363 | 52.5 | 379 | 54.6 |
| No Low Wellbeing | 3313 | 78.2 | 1353 | 42.8 | 1515 | 47.1 | 1499 | 47.2 | 1457 | 45.5 | 1528 | 48.0 |
| Low Wellbeing | 924 | 21.8 | 472 | 53.0 | 466 | 51.4 | 523 | 58.6 | 508 | 56.4 | 514 | 57.3 |
| **Current Risk Behaviours** | | | | | | | | | | | | |
| Low-Risk Drinking | 3236 | 62.5 | 1499 | 47.9 | 1489 | 46.7 | 1678 | 53.4 | 1624 | 51.2 | 1625 | 51.6 |
| High-Risk Drinking | 1944 | 37.5 | 805 | 42.6 | 957 | 49.9 | 879 | 46.4 | 841 | 44.1 | 934 | 49.2 |
| No Increased Alcohol Use | 2493 | 55.9 | 1012 | 42.0 | 1157 | 47.2 | 1132 | 46.8 | 1099 | 45.1 | 1163 | 47.9 |
| Increased Alcohol Use | 1971 | 44.2 | 934 | 48.6 | 970 | 49.7 | 1030 | 53.5 | 973 | 50.1 | 1023 | 53.0 |
| No Increased Smoking/E-Cigarette Use | 587 | 59.5 | 240 | 43.1 | 257 | 45.0 | 272 | 48.8 | 264 | 46.4 | 275 | 49.3 |
| Increased Smoking/E-Cigarette Use | 399 | 40.5 | 198 | 51.6 | 188 | 48.0 | 213 | 55.3 | 193 | 49.7 | 228 | 58.8 |
| No Self-Isolating Given COVID-19 | 497 | 63.1 | 199 | 41.3 | 240 | 48.8 | 225 | 46.7 | 210 | 43.1 | 246 | 50.7 |
| Self-Isolating Given COVID-19 | 291 | 36.9 | 137 | 49.6 | 194 | 68.1 | 136 | 48.8 | 107 | 38.5 | 165 | 58.1 |
| No Face-To-Face Contact | 1760 | 33.9 | 830 | 48.5 | 878 | 50.5 | 915 | 53.3 | 841 | 48.5 | 918 | 53.3 |
| ≥1 Face-To-Face Contact | 3427 | 66.1 | 1485 | 44.8 | 1576 | 46.7 | 1651 | 49.6 | 1630 | 48.6 | 1654 | 49.6 |
| No Physical Contact | 3752 | 77.0 | 1703 | 46.7 | 1836 | 49.5 | 1887 | 51.7 | 1812 | 49.2 | 1902 | 51.9 |
| ≥1 Physical Contact | 1124 | 23.1 | 463 | 42.6 | 483 | 43.8 | 517 | 47.4 | 492 | 44.8 | 513 | 46.9 |
| **Pre-pandemic Risk Behaviours** | | | | | | | | | | | | |
| Low-Risk Drinking | 2230 | 57.3 | 1013 | 47.2 | 1042 | 47.8 | 1130 | 52.5 | 1132 | 52.3 | 1091 | 50.6 |
| High-Risk Drinking | 1659 | 42.7 | 658 | 41.3 | 797 | 49.3 | 716 | 44.8 | 659 | 40.9 | 782 | 48.8 |
| No Smoking | 3645 | 84.7 | 1574 | 44.9 | 1713 | 48.1 | 1746 | 49.7 | 1708 | 48.2 | 1745 | 49.5 |
| Smoking | 660 | 15.3 | 290 | 46.1 | 316 | 49.6 | 319 | 50.6 | 294 | 46.4 | 351 | 55.6 |
| **Early Pandemic Risk Behaviours** | | | | | | | | | | | | |
| No Self-Isolating Given COVID-19 | 328 | 79.0 | 136 | 43.3 | 150 | 46.3 | 147 | 46.8 | 145 | 45.5 | 156 | 49.4 |
| Self-Isolating Given COVID-19 | 87 | 21.0 | 42 | 52.5 | 45 | 52.3 | 47 | 58.0 | 42 | 51.9 | 48 | 56.5 |
| No Face-To-Face Contact | 1630 | 38.6 | 724 | 46.1 | 823 | 51.8 | 794 | 50.4 | 748 | 47.2 | 817 | 51.8 |
| ≥1 Face-To-Face Contact | 2590 | 61.4 | 1115 | 44.9 | 1172 | 46.3 | 1237 | 49.6 | 1191 | 47.4 | 1237 | 49.4 |
| No Physical Contact | 3155 | 84.0 | 1399 | 46.0 | 1535 | 49.7 | 1530 | 50.1 | 1465 | 47.7 | 1557 | 50.8 |
| ≥1 Physical Contact | 602 | 16.0 | 243 | 42.3 | 261 | 44.6 | 278 | 48.3 | 268 | 46.0 | 277 | 48.0 |

*Note.* Complete sample refers to participants who completed ALSPAC’s second COVID-19 questionnaire.

**Supplementary Table S3**

*Frequencies and Percentages of COVID-19 Risk Perceptions by Sociodemographic, Health, and Risk Behaviour Variables (Mothers)*

|  | **COVID-19 Risk Perceptions (High Only)** | | | | | | | | | | | |
| --- | --- | --- | --- | --- | --- | --- | --- | --- | --- | --- | --- | --- |
|  | **Complete Sample** | | **Holistic** | | **Cognitive** | | **Affective** | | **Self** | | **Other** | |
| **Participant Characteristic** | **N** | **%** | **N** | **%** | **N** | **%** | **N** | **%** | **N** | **%** | **N** | **%** |
| **Complete Sample** | - | - | 1251 | 49.9 | 1117 | 43.8 | 1237 | 49.2 | 1091 | 43.2 | 1255 | 49.6 |
| **Sociodemographic** | | | | | | | | | | | | |
| <60 Years of Age | 1649 | 63.2 | 813 | 51.1 | 703 | 43.5 | 805 | 50.5 | 701 | 43.8 | 822 | 51.2 |
| ≥60 Years of Age | 960 | 36.8 | 438 | 47.8 | 414 | 44.2 | 432 | 47.0 | 390 | 42.0 | 433 | 46.9 |
| White Ethnicity | 2449 | 98.4 | 1169 | 49.7 | 1048 | 43.7 | 1159 | 49.1 | 1013 | 42.7 | 1172 | 49.4 |
| Non-White Ethnicity | 40 | 1.6 | 20 | 51.3 | 12 | 30.8 | 20 | 51.3 | 18 | 46.2 | 20 | 51.3 |
| No University Education | 1899 | 76.1 | 957 | 52.6 | 783 | 42.2 | 961 | 52.7 | 844 | 46.0 | 946 | 51.6 |
| University Education | 595 | 23.9 | 233 | 40.3 | 281 | 48.0 | 219 | 37.7 | 190 | 32.6 | 248 | 42.5 |
| Not a Key Worker | 1618 | 65.6 | 768 | 48.5 | 696 | 43.2 | 760 | 47.8 | 680 | 42.6 | 773 | 48.4 |
| Key Worker | 849 | 34.4 | 423 | 51.0 | 376 | 44.4 | 417 | 50.2 | 356 | 42.5 | 423 | 50.5 |
| Not a Health/Social Care Worker | 2111 | 83.5 | 1027 | 49.7 | 908 | 43.2 | 1017 | 49.1 | 906 | 43.5 | 1027 | 49.3 |
| Health/Social Care Worker | 418 | 16.5 | 199 | 48.7 | 192 | 46.0 | 194 | 47.3 | 160 | 38.7 | 203 | 49.4 |
| **Current Health** | | | | | | | | | | | | |
| No Probable Generalised Anxiety | 2263 | 90.2 | 1047 | 47.3 | 957 | 42.5 | 1030 | 46.4 | 900 | 40.3 | 1051 | 47.1 |
| Probable Generalised Anxiety | 247 | 9.8 | 184 | 75.7 | 138 | 55.9 | 187 | 77.0 | 174 | 71.3 | 185 | 75.5 |
| No Likely Depression | 2320 | 94.6 | 1117 | 49.2 | 1011 | 43.7 | 1101 | 48.3 | 973 | 42.5 | 1117 | 48.8 |
| Likely Depression | 133 | 5.4 | 87 | 66.9 | 65 | 49.2 | 89 | 68.5 | 77 | 59.2 | 88 | 66.7 |
| No Low Mental Wellbeing | 2014 | 81.7 | 910 | 46.1 | 855 | 42.6 | 894 | 45.2 | 800 | 40.2 | 921 | 46.3 |
| Low Mental Wellbeing | 452 | 18.3 | 295 | 67.1 | 229 | 50.9 | 298 | 67.4 | 250 | 56.3 | 291 | 65.0 |
| No Medical Conditions | 1043 | 50.3 | 436 | 43.3 | 427 | 41.7 | 417 | 41.3 | 355 | 35.1 | 461 | 45.5 |
| ≥1 Medical Conditions | 1032 | 49.7 | 543 | 54.6 | 462 | 45.5 | 541 | 54.3 | 500 | 49.7 | 530 | 52.7 |
| Not Self-Isolating as Not Vulnerable | 2398 | 94.1 | 1127 | 48.4 | 1016 | 42.9 | 1110 | 47.6 | 974 | 41.5 | 1136 | 48.4 |
| Self-Isolating as Vulnerable | 151 | 5.9 | 104 | 70.8 | 81 | 54.4 | 107 | 72.3 | 98 | 65.8 | 97 | 65.5 |
| Not Live with Vulnerable Person | 2209 | 88.1 | 1062 | 49.1 | 969 | 44.0 | 1052 | 48.5 | 922 | 42.3 | 1067 | 48.9 |
| Live with Vulnerable Person | 298 | 11.9 | 151 | 51.9 | 123 | 41.6 | 147 | 50.2 | 132 | 44.8 | 155 | 52.9 |
| No Suspected COVID-19 (Self-Report) | 2275 | 87.4 | 1093 | 49.8 | 958 | 43.1 | 1083 | 49.3 | 960 | 43.4 | 1079 | 48.9 |
| Suspected COVID-19 (Self-Report) | 328 | 12.6 | 156 | 50.8 | 157 | 48.8 | 153 | 49.5 | 129 | 41.5 | 174 | 55.1 |
| No Suspected COVID-19 (Algorithm) | 2562 | 98.2 | 1229 | 49.9 | 1093 | 43.6 | 1213 | 49.1 | 1070 | 43.1 | 1231 | 49.5 |
| Suspected COVID-19 (Algorithm) | 47 | 1.8 | 22 | 51.2 | 24 | 54.6 | 24 | 54.6 | 21 | 47.7 | 24 | 54.6 |
| **Pre-pandemic Health** | | | | | | | | | | | | |
| No Anxiety | 1749 | 74.7 | 811 | 48.1 | 750 | 43.7 | 792 | 46.9 | 702 | 41.3 | 809 | 47.6 |
| Anxiety | 593 | 25.3 | 306 | 53.9 | 264 | 45.8 | 308 | 53.9 | 268 | 47.0 | 320 | 55.6 |
| No Depression | 1855 | 79.1 | 876 | 49.1 | 807 | 44.3 | 865 | 48.3 | 769 | 42.7 | 881 | 48.9 |
| Depression | 489 | 20.9 | 241 | 51.2 | 209 | 44.0 | 235 | 49.7 | 201 | 42.6 | 249 | 52.4 |
| No Low Wellbeing | 1600 | 74.6 | 719 | 46.5 | 666 | 42.3 | 697 | 45.0 | 609 | 39.1 | 722 | 46.3 |
| Low Wellbeing | 544 | 25.4 | 291 | 55.4 | 259 | 48.6 | 296 | 56.2 | 252 | 47.7 | 302 | 56.9 |
| **Current Risk Behaviours** | | | | | | | | | | | | |
| Low-Risk Drinking | 1691 | 66.4 | 823 | 50.2 | 713 | 42.7 | 819 | 49.8 | 720 | 43.6 | 824 | 49.9 |
| High-Risk Drinking | 854 | 33.6 | 405 | 48.7 | 387 | 45.8 | 394 | 47.4 | 349 | 41.8 | 407 | 48.5 |
| No Increased Alcohol Use | 1232 | 56.5 | 536 | 45.0 | 522 | 43.0 | 532 | 44.6 | 473 | 39.4 | 549 | 45.7 |
| Increased Alcohol Use | 950 | 43.5 | 510 | 54.7 | 427 | 45.2 | 499 | 53.4 | 434 | 46.2 | 499 | 53.3 |
| No Increased Smoking/E-Cigarette Use | 207 | 64.7 | 99 | 49.8 | 90 | 44.6 | 95 | 47.7 | 96 | 47.8 | 95 | 47.5 |
| Increased Smoking/E-Cigarette Use | 113 | 35.3 | 71 | 65.1 | 54 | 48.2 | 65 | 59.6 | 63 | 57.8 | 66 | 58.9 |
| No Self-Isolating Given COVID-19 | 224 | 70.0 | 102 | 47.9 | 102 | 46.2 | 100 | 47.0 | 88 | 40.7 | 115 | 53.2 |
| Self-Isolating Given COVID-19 | 96 | 30.0 | 49 | 55.7 | 53 | 56.4 | 49 | 54.4 | 37 | 41.6 | 55 | 58.5 |
| No Face-To-Face Contact | 659 | 25.7 | 347 | 54.6 | 294 | 45.3 | 344 | 53.9 | 310 | 48.2 | 334 | 52.0 |
| ≥1 Face-To-Face Contact | 1902 | 74.3 | 890 | 48.2 | 814 | 43.3 | 879 | 47.4 | 767 | 41.2 | 909 | 48.8 |
| No Physical Contact | 1770 | 75.3 | 876 | 50.9 | 791 | 45.2 | 869 | 50.3 | 768 | 44.3 | 883 | 50.8 |
| ≥1 Physical Contact | 582 | 24.7 | 261 | 46.3 | 233 | 40.7 | 259 | 45.8 | 224 | 39.4 | 262 | 46.1 |
| **Pre-pandemic Risk Behaviours** | | | | | | | | | | | | |
| Low-Risk Drinking | 1353 | 70.5 | 632 | 48.4 | 586 | 44.1 | 623 | 47.5 | 529 | 40.1 | 644 | 48.9 |
| High-Risk Drinking | 566 | 29.5 | 262 | 47.9 | 248 | 44.6 | 254 | 46.4 | 233 | 42.4 | 267 | 48.1 |
| No Smoking | 1975 | 92.5 | 909 | 47.6 | 841 | 43.3 | 897 | 46.8 | 773 | 40.1 | 927 | 48.1 |
| Smoking | 160 | 7.5 | 86 | 57.0 | 77 | 50.0 | 83 | 54.6 | 81 | 53.3 | 83 | 54.3 |
| **Early Pandemic Risk Behaviours** | | | | | | | | | | | | |
| No Self-Isolating Given COVID-19 | 186 | 68.1 | 88 | 50.0 | 80 | 43.2 | 85 | 48.3 | 76 | 42.2 | 95 | 53.4 |
| Self-Isolating Given COVID-19 | 87 | 31.9 | 42 | 52.5 | 45 | 52.3 | 44 | 54.3 | 36 | 44.4 | 48 | 56.5 |
| No Face-To-Face Contact | 696 | 32.6 | 336 | 50.1 | 309 | 45.2 | 325 | 48.2 | 291 | 43.0 | 338 | 49.9 |
| ≥1 Face-To-Face Contact | 1442 | 67.5 | 674 | 48.5 | 617 | 43.5 | 662 | 47.5 | 580 | 41.3 | 685 | 48.8 |
| No Physical Contact | 1589 | 84.8 | 759 | 49.3 | 696 | 44.4 | 741 | 48.0 | 660 | 42.6 | 766 | 49.2 |
| ≥1 Physical Contact | 285 | 15.2 | 122 | 45.0 | 113 | 40.8 | 124 | 45.6 | 105 | 38.0 | 127 | 46.7 |

*Note.* Complete sample refers to participants who completed ALSPAC’s second COVID-19 questionnaire.

**Supplementary Table S4**

*Frequencies and Percentages of COVID-19 Risk Perceptions by Sociodemographic, Health, and Risk Behaviour Variables (Young People)*

|  | **COVID-19 Risk Perceptions (High Only)** | | | | | | | | | | | |
| --- | --- | --- | --- | --- | --- | --- | --- | --- | --- | --- | --- | --- |
|  | **Complete Sample** | | **Holistic** | | **Cognitive** | | **Affective** | | **Self** | | **Other** | |
| **Participant Characteristic** | **N** | **%** | **N** | **%** | **N** | **%** | **N** | **%** | **N** | **%** | **N** | **%** |
| **Complete Sample** | - | - | 1248 | 48.8 | 606 | 23.3 | 1178 | 45.9 | 1016 | 39.2 | 1022 | 39.8 |
| **Sociodemographic** | | | | | | | | | | | | |
| Female | 1912 | 70.6 | 996 | 55.1 | 455 | 24.7 | 950 | 52.5 | 816 | 44.5 | 818 | 45.1 |
| Male | 795 | 29.4 | 251 | 33.6 | 151 | 20.0 | 227 | 30.2 | 199 | 26.3 | 203 | 27.1 |
| White Ethnicity | 2338 | 96.6 | 1060 | 48.0 | 530 | 23.6 | 996 | 45.0 | 865 | 38.6 | 867 | 39.2 |
| Non-White Ethnicity | 82 | 3.4 | 46 | 57.5 | 22 | 27.5 | 40 | 50.0 | 29 | 36.3 | 37 | 46.3 |
| No University Education | 598 | 27.1 | 304 | 54.5 | 89 | 15.5 | 309 | 55.4 | 271 | 47.7 | 236 | 42.0 |
| University Education | 1606 | 72.9 | 707 | 45.8 | 406 | 26.0 | 638 | 41.3 | 546 | 35.0 | 589 | 38.1 |
| Not a Key Worker | 1526 | 61.1 | 711 | 47.4 | 340 | 22.4 | 668 | 44.4 | 570 | 37.5 | 589 | 39.2 |
| Key Worker | 971 | 38.9 | 495 | 51.9 | 245 | 25.3 | 468 | 49.0 | 411 | 42.5 | 393 | 41.0 |
| Not a Health/Social Care Worker | 2203 | 85.6 | 1032 | 47.7 | 499 | 22.7 | 975 | 45.0 | 846 | 38.6 | 839 | 38.7 |
| Health/Social Care Worker | 370 | 14.4 | 206 | 56.4 | 105 | 28.4 | 192 | 52.6 | 162 | 43.9 | 173 | 47.3 |
| **Current Health** | | | | | | | | | | | | |
| No Probable Generalised Anxiety | 1960 | 76.2 | 820 | 42.6 | 421 | 21.5 | 757 | 39.3 | 655 | 33.6 | 657 | 34.1 |
| Probable Generalised Anxiety | 611 | 23.8 | 412 | 68.3 | 179 | 29.3 | 403 | 66.7 | 349 | 57.2 | 352 | 58.3 |
| No Likely Depression | 2087 | 81.3 | 930 | 45.3 | 456 | 21.9 | 868 | 42.3 | 750 | 36.1 | 752 | 36.6 |
| Likely Depression | 480 | 18.7 | 296 | 63.0 | 135 | 28.2 | 287 | 60.9 | 247 | 51.8 | 252 | 53.5 |
| No Low Mental Wellbeing | 1745 | 67.8 | 764 | 44.5 | 387 | 22.2 | 706 | 41.1 | 609 | 35.0 | 633 | 36.8 |
| Low Mental Wellbeing | 829 | 32.2 | 468 | 57.6 | 213 | 25.8 | 456 | 56.1 | 396 | 48.0 | 374 | 46.0 |
| No Medical Conditions | 1383 | 66.2 | 590 | 44.5 | 305 | 22.7 | 541 | 40.7 | 447 | 33.3 | 495 | 37.3 |
| ≥1 Medical Conditions | 706 | 33.8 | 376 | 56.0 | 170 | 25.0 | 365 | 54.2 | 313 | 46.2 | 297 | 44.1 |
| Not Self-Isolating as Not Vulnerable | 2504 | 96.0 | 1156 | 47.6 | 571 | 23.2 | 1084 | 44.5 | 924 | 37.5 | 952 | 39.1 |
| Self-Isolating as Vulnerable | 105 | 4.0 | 75 | 74.3 | 31 | 30.1 | 74 | 73.3 | 75 | 72.8 | 54 | 53.5 |
| Not Live with Vulnerable Person | 2355 | 91.9 | 1114 | 48.1 | 560 | 23.8 | 1048 | 45.2 | 910 | 38.8 | 909 | 39.2 |
| Live with Vulnerable Person | 208 | 8.1 | 120 | 58.5 | 41 | 19.7 | 116 | 56.6 | 96 | 46.4 | 98 | 47.6 |
| No Suspected COVID-19 (Self-Report) | 2229 | 82.3 | 1027 | 49.0 | 471 | 22.1 | 988 | 47.1 | 862 | 40.6 | 833 | 39.7 |
| Suspected COVID-19 (Self-Report) | 478 | 17.7 | 220 | 47.7 | 135 | 29.0 | 189 | 40.9 | 154 | 33.2 | 188 | 40.6 |
| No Suspected COVID-19 (Algorithm) | 2597 | 95.9 | 1186 | 48.4 | 580 | 23.3 | 1115 | 45.4 | 967 | 38.9 | 967 | 39.4 |
| Suspected COVID-19 (Algorithm) | 112 | 4.1 | 61 | 57.6 | 26 | 24.1 | 62 | 58.5 | 49 | 45.8 | 54 | 50.5 |
| **Pre-pandemic Health** | | | | | | | | | | | | |
| No Anxiety | 1785 | 88.7 | 797 | 46.3 | 406 | 23.2 | 730 | 42.3 | 632 | 36.2 | 646 | 37.4 |
| Anxiety | 227 | 11.3 | 115 | 61.2 | 60 | 31.3 | 109 | 57.7 | 90 | 47.1 | 100 | 52.9 |
| No Depression | 1785 | 88.7 | 787 | 46.4 | 405 | 23.4 | 720 | 42.3 | 621 | 36.1 | 643 | 37.7 |
| Depression | 227 | 11.3 | 126 | 57.5 | 62 | 28.2 | 119 | 54.3 | 101 | 45.9 | 104 | 47.5 |
| No Low Wellbeing | 1713 | 81.8 | 745 | 46.0 | 372 | 22.6 | 693 | 42.7 | 604 | 36.8 | 615 | 37.9 |
| Low Wellbeing | 380 | 18.2 | 207 | 56.6 | 102 | 27.4 | 194 | 53.0 | 172 | 46.2 | 166 | 45.4 |
| **Current Risk Behaviours** | | | | | | | | | | | | |
| Low-Risk Drinking | 1545 | 58.6 | 771 | 51.6 | 344 | 22.6 | 745 | 49.8 | 648 | 42.7 | 625 | 41.7 |
| High-Risk Drinking | 1090 | 41.4 | 472 | 44.6 | 259 | 24.1 | 428 | 40.3 | 367 | 34.3 | 392 | 37.0 |
| No Increased Alcohol Use | 1261 | 55.3 | 566 | 46.3 | 279 | 22.5 | 522 | 42.7 | 441 | 35.6 | 472 | 38.6 |
| Increased Alcohol Use | 1021 | 44.7 | 484 | 48.9 | 247 | 24.5 | 460 | 46.4 | 392 | 39.1 | 395 | 39.7 |
| No Increased Smoking/E-Cigarette Use | 380 | 57.1 | 166 | 46.4 | 70 | 19.0 | 164 | 45.8 | 142 | 38.6 | 143 | 39.9 |
| Increased Smoking/E-Cigarette Use | 286 | 42.9 | 144 | 52.4 | 64 | 22.9 | 138 | 50.0 | 120 | 43.0 | 125 | 45.3 |
| No Self-Isolating Given COVID-19 | 273 | 58.3 | 118 | 43.9 | 67 | 24.7 | 107 | 39.8 | 92 | 34.0 | 100 | 37.2 |
| Self-Isolating Given COVID-19 | 195 | 41.7 | 100 | 53.2 | 68 | 35.6 | 81 | 42.9 | 60 | 31.8 | 87 | 45.8 |
| No Face-To-Face Contact | 1101 | 41.9 | 545 | 50.7 | 258 | 23.7 | 527 | 48.9 | 444 | 40.7 | 452 | 41.9 |
| ≥1 Face-To-Face Contact | 1525 | 58.1 | 698 | 47.5 | 345 | 23.1 | 645 | 43.8 | 567 | 38.0 | 568 | 38.5 |
| No Physical Contact | 1982 | 78.5 | 957 | 49.8 | 465 | 23.8 | 900 | 46.8 | 785 | 40.2 | 777 | 40.3 |
| ≥1 Physical Contact | 542 | 21.5 | 232 | 44.3 | 114 | 21.5 | 220 | 41.9 | 185 | 34.9 | 188 | 35.7 |
| **Pre-pandemic Risk Behaviours** | | | | | | | | | | | | |
| Low-Risk Drinking | 877 | 44.5 | 433 | 51.7 | 191 | 22.5 | 416 | 49.6 | 373 | 44.1 | 345 | 41.0 |
| High-Risk Drinking | 1093 | 55.5 | 466 | 44.6 | 259 | 24.4 | 426 | 40.6 | 360 | 33.9 | 387 | 37.0 |
| No Smoking | 1670 | 77.0 | 775 | 48.6 | 387 | 23.9 | 714 | 44.7 | 630 | 39.0 | 631 | 39.4 |
| Smoking | 500 | 23.0 | 239 | 50.0 | 112 | 23.2 | 231 | 48.2 | 192 | 39.8 | 198 | 41.4 |
| No E-Cigarette Use | 2076 | 95.7 | 968 | 48.7 | 480 | 23.9 | 897 | 45.1 | 787 | 39.2 | 793 | 39.8 |
| E-Cigarette Use | 94 | 4.3 | 46 | 52.9 | 19 | 21.6 | 48 | 54.6 | 35 | 39.8 | 36 | 41.4 |
| **Early Pandemic Risk Behaviours** | | | | | | | | | | | | |
| No Self-Isolating Given COVID-19 | 142 | 100.0 | 57 | 41.3 | 33 | 23.7 | 50 | 36.2 | 43 | 30.9 | 48 | 34.8 |
| Self-Isolating Given COVID-19 | 0 | 0 | 0 | 0 | 0 | 0 | 0 | 0 | 0 | 0 | 0 | 0 |
| No Face-To-Face Contact | 934 | 44.9 | 450 | 50.1 | 225 | 24.8 | 419 | 46.5 | 364 | 40.1 | 375 | 41.7 |
| ≥1 Face-To-Face Contact | 1148 | 55.1 | 515 | 47.0 | 248 | 22.3 | 483 | 44.0 | 394 | 35.5 | 413 | 37.6 |
| No Physical Contact | 1566 | 83.2 | 740 | 49.2 | 373 | 24.5 | 685 | 45.4 | 585 | 38.4 | 671 | 40.9 |
| ≥1 Physical Contact | 317 | 16.8 | 144 | 47.4 | 67 | 21.8 | 138 | 45.4 | 116 | 37.8 | 109 | 35.7 |

*Note.* Complete sample refers to participants who completed ALSPAC’s second COVID-19 questionnaire.

**Supplementary Table S5**

*Complete Case* *Frequencies and Percentages of COVID-19 Risk Perceptions by Sociodemographic, Health, and Risk Behaviour Variables (Whole Sample)*

|  | **COVID-19 Risk Perceptions (High Only)** | | | | | | | | | | | |
| --- | --- | --- | --- | --- | --- | --- | --- | --- | --- | --- | --- | --- |
|  | **Complete Sample** | | **Holistic** | | **Cognitive** | | **Affective** | | **Self** | | **Other** | |
| **Participant Characteristic** | **N** | **%** | **N** | **%** | **N** | **%** | **N** | **%** | **N** | **%** | **N** | **%** |
| **Complete Sample** | - | - | 1969 | 45.6 | 2083 | 47.4 | 2191 | 50.6 | 2127 | 48.7 | 2173 | 50.0 |
| **Sociodemographic** | | | | | | | | | | | | |
| 27-30 Years of Age (Young People) | 2049 | 46.5 | 838 | 41.6 | 1064 | 52.0 | 909 | 45.0 | 787 | 38.6 | 1037 | 51.3 |
| <60 Years of Age (Mothers) | 1496 | 33.9 | 740 | 50.4 | 640 | 42.9 | 839 | 57.0 | 857 | 58.0 | 749 | 50.6 |
| ≥60 Years of Age (Mothers) | 863 | 19.6 | 391 | 46.6 | 379 | 44.2 | 443 | 52.6 | 483 | 56.8 | 387 | 45.7 |
| Female | 3829 | 86.9 | 1815 | 48.4 | 1810 | 47.4 | 2023 | 53.8 | 1975 | 52.1 | 1963 | 52.0 |
| Male | 579 | 13.1 | 154 | 27.0 | 273 | 47.3 | 168 | 29.4 | 152 | 26.3 | 210 | 36.7 |
| White Ethnicity | 4014 | 97.7 | 1832 | 45.6 | 1949 | 47.7 | 2030 | 50.4 | 1991 | 49.0 | 2009 | 49.6 |
| Non-White Ethnicity | 98 | 2.33 | 48 | 49.0 | 41 | 41.8 | 55 | 56.1 | 45 | 45.9 | 57 | 58.2 |
| No University Education | 2331 | 52.9 | 1171 | 51.5 | 978 | 42.1 | 1328 | 58.3 | 1323 | 57.5 | 1183 | 51.6 |
| University Education | 2077 | 47.1 | 798 | 39.0 | 1105 | 53.4 | 863 | 42.0 | 804 | 38.9 | 990 | 48.2 |
| Not a Key Worker | 2800 | 63.5 | 1227 | 44.7 | 1306 | 46.8 | 1366 | 49.6 | 1335 | 48.1 | 1359 | 49.2 |
| Key Worker | 1608 | 36.5 | 742 | 47.1 | 777 | 48.4 | 825 | 52.3 | 792 | 49.7 | 814 | 51.2 |
| Not a Healthcare Worker | 3698 | 83.9 | 1635 | 45.1 | 1730 | 47.0 | 1817 | 50.0 | 1772 | 48.4 | 1800 | 49.3 |
| Healthcare Worker | 710 | 16.1 | 334 | 47.9 | 535 | 49.8 | 374 | 53.5 | 355 | 50.3 | 373 | 53.3 |
| **Current Health** | | | | | | | | | | | | |
| No Probable Generalised Anxiety | 3655 | 84.5 | 1491 | 41.2 | 1667 | 45.8 | 1678 | 46.7 | 1654 | 45.7 | 1655 | 46.0 |
| Probable Generalised Anxiety | 671 | 15.5 | 448 | 67.4 | 384 | 57.2 | 476 | 71.6 | 438 | 65.7 | 485 | 72.6 |
| No Likely Depression | 3815 | 89.2 | 1645 | 44.0 | 1784 | 46.9 | 1834 | 48.9 | 1802 | 47.6 | 1808 | 48.0 |
| Likely Depression | 464 | 10.8 | 269 | 59.0 | 247 | 53.4 | 290 | 63.6 | 265 | 57.9 | 305 | 66.5 |
| No Low Mental Wellbeing | 3275 | 76.3 | 1348 | 42.0 | 1505 | 46.1 | 1512 | 46.9 | 1489 | 45.9 | 1509 | 46.7 |
| Low Mental Wellbeing | 1020 | 23.8 | 570 | 57.0 | 537 | 52.8 | 620 | 61.9 | 580 | 57.4 | 614 | 61.0 |
| No Medical Conditions | 2077 | 58.4 | 809 | 39.7 | 969 | 46.8 | 898 | 44.0 | 838 | 40.7 | 953 | 46.6 |
| ≥1 Medical Conditions | 1482 | 41.6 | 745 | 51.2 | 715 | 48.4 | 835 | 57.3 | 822 | 56.0 | 784 | 53.5 |
| Not Self-Isolating as Not Vulnerable | 4144 | 95.0 | 1791 | 44.1 | 1930 | 46.7 | 2002 | 49.2 | 1930 | 47.0 | 2003 | 49.0 |
| Self-Isolating as Vulnerable | 217 | 5.0 | 151 | 70.9 | 127 | 58.8 | 158 | 73.8 | 166 | 76.9 | 141 | 65.9 |
| Not Live with Vulnerable Person | 3931 | 90.2 | 1732 | 44.9 | 1870 | 47.7 | 1926 | 49.8 | 1868 | 48.0 | 1918 | 49.5 |
| Live with Vulnerable Person | 427 | 9.8 | 211 | 50.4 | 190 | 44.7 | 238 | 56.5 | 231 | 54.5 | 230 | 54.5 |
| No Suspected COVID-19 (Self-Report) | 3736 | 84.9 | 1677 | 45.7 | 1717 | 46.1 | 1874 | 51.0 | 1842 | 50.0 | 1819 | 49.4 |
| Suspected COVID-19 (Self-Report) | 666 | 15.1 | 290 | 44.8 | 364 | 54.8 | 315 | 48.5 | 283 | 43.4 | 352 | 53.5 |
| No Suspected COVID-19 (Algorithm) | 4288 | 97.3 | 1906 | 45.3 | 2016 | 47.2 | 2120 | 50.3 | 2060 | 48.5 | 2104 | 49.7 |
| Suspected COVID-19 (Algorithm) | 120 | 2.7 | 63 | 53.9 | 67 | 56.3 | 71 | 60.2 | 65 | 55.1 | 69 | 58.0 |
| **Pre-pandemic Health** | | | | | | | | | | | | |
| No Anxiety | 3057 | 81.7 | 1293 | 43.1 | 1427 | 46.8 | 1428 | 47.5 | 1414 | 46.6 | 1454 | 48.2 |
| Anxiety | 687 | 18.4 | 367 | 54.5 | 341 | 49.9 | 411 | 60.7 | 387 | 57.2 | 392 | 57.7 |
| No Depression | 3144 | 83.8 | 1358 | 44.1 | 1483 | 47.3 | 1497 | 48.5 | 1487 | 47.8 | 1525 | 49.2 |
| Depression | 607 | 16.2 | 303 | 50.4 | 290 | 47.9 | 341 | 56.6 | 319 | 53.0 | 324 | 53.6 |
| No Low Wellbeing | 2878 | 78.5 | 1192 | 42.3 | 1341 | 46.7 | 1324 | 46.8 | 1032 | 45.6 | 1347 | 47.5 |
| Low Wellbeing | 790 | 21.5 | 413 | 53.2 | 400 | 50.8 | 458 | 58.8 | 441 | 56.3 | 445 | 56.9 |
| **Current Risk Behaviours** | | | | | | | | | | | | |
| Low-Risk Drinking | 2773 | 63.4 | 1280 | 47.2 | 1281 | 46.4 | 1436 | 52.7 | 1390 | 50.6 | 1382 | 50.6 |
| High-Risk Drinking | 1598 | 36.6 | 667 | 42.4 | 786 | 49.3 | 731 | 46.5 | 712 | 45.0 | 768 | 48.6 |
| No Increased Alcohol Use | 2117 | 56.2 | 848 | 40.8 | 978 | 46.3 | 955 | 45.9 | 935 | 44.6 | 976 | 46.7 |
| Increased Alcohol Use | 1648 | 43.8 | 794 | 49.0 | 811 | 49.3 | 875 | 53.9 | 833 | 50.9 | 862 | 52.9 |
| No Increased Smoking/E-Cigarette Use | 443 | 59.8 | 183 | 42.3 | 196 | 44.2 | 209 | 48.3 | 206 | 46.7 | 202 | 46.5 |
| Increased Smoking/E-Cigarette Use | 298 | 40.2 | 152 | 51.9 | 146 | 49.0 | 162 | 55.3 | 151 | 51.2 | 171 | 57.8 |
| No Self-Isolating Given COVID-19 | 418 | 63.6 | 169 | 41.3 | 197 | 47.1 | 194 | 47.4 | 185 | 44.8 | 209 | 50.7 |
| Self-Isolating Given COVID-19 | 239 | 36.4 | 115 | 50.2 | 163 | 68.8 | 115 | 49.8 | 91 | 39.6 | 138 | 58.2 |
| No Face-To-Face Contact | 1478 | 33.8 | 696 | 48.0 | 735 | 49.9 | 770 | 53.0 | 710 | 48.4 | 769 | 52.7 |
| ≥1 Face-To-Face Contact | 2901 | 66.3 | 1260 | 44.3 | 1337 | 46.2 | 1404 | 49.3 | 1398 | 48.7 | 1392 | 48.6 |
| No Physical Contact | 3175 | 77.0 | 1427 | 45.9 | 1546 | 48.9 | 1588 | 50.9 | 1538 | 48.9 | 1589 | 50.8 |
| ≥1 Physical Contact | 950 | 23.0 | 403 | 43.1 | 415 | 43.8 | 450 | 48.0 | 430 | 45.7 | 440 | 46.8 |
| **Pre-pandemic Risk Behaviours** | | | | | | | | | | | | |
| Low-Risk Drinking | 1969 | 58.0 | 895 | 46.3 | 930 | 47.4 | 1001 | 51.6 | 1006 | 51.6 | 963 | 49.5 |
| High-Risk Drinking | 1424 | 42.0 | 574 | 41.0 | 688 | 48.4 | 632 | 45.1 | 580 | 41.0 | 686 | 48.8 |
| No Smoking | 3217 | 86.1 | 1400 | 44.3 | 1520 | 47.4 | 1559 | 49.2 | 1526 | 47.9 | 1553 | 48.9 |
| Smoking | 520 | 13.9 | 240 | 47.0 | 256 | 49.3 | 263 | 51.4 | 246 | 47.7 | 288 | 56.1 |
| **Early Pandemic Risk Behaviours** | | | | | | | | | | | | |
| No Self-Isolating Given COVID-19 | 288 | 78.5 | 126 | 45.0 | 136 | 47.2 | 137 | 48.9 | 133 | 46.8 | 144 | 51.1 |
| Self-Isolating Given COVID-19 | 79 | 21.5 | 37 | 51.4 | 40 | 51.3 | 42 | 57.5 | 37 | 50.7 | 43 | 55.8 |
| No Face-To-Face Contact | 1384 | 38.3 | 622 | 45.7 | 703 | 51.0 | 676 | 49.5 | 644 | 46.8 | 693 | 50.7 |
| ≥1 Face-To-Face Contact | 2231 | 61.7 | 966 | 44.2 | 1014 | 45.6 | 1080 | 49.4 | 1044 | 47.3 | 1070 | 48.7 |
| No Physical Contact | 2702 | 83.8 | 1204 | 45.4 | 1316 | 48.9 | 1315 | 49.4 | 1268 | 47.4 | 1335 | 50.0 |
| ≥1 Physical Contact | 521 | 16.2 | 219 | 42.9 | 230 | 44.2 | 251 | 49.0 | 243 | 46.9 | 248 | 48.3 |

*Note.* Complete sample = participants who completed ALSPAC’s second COVID-19 questionnaire. Complete case = participants who had complete data on the covariates included in the partially adjusted regression models (i.e., age, gender, education, and keyworker status).

**Supplementary Table S6**

*Complete Case* *Frequencies and Percentages of COVID-19 Risk Perceptions by Sociodemographic, Health, and Risk Behaviour Variables (Mothers)*

|  | **COVID-19 Risk Perceptions (High Only)** | | | | | | | | | | | |
| --- | --- | --- | --- | --- | --- | --- | --- | --- | --- | --- | --- | --- |
|  | **Complete Sample** | | **Holistic** | | **Cognitive** | | **Affective** | | **Self** | | **Other** | |
| **Participant Characteristic** | **N** | **%** | **N** | **%** | **N** | **%** | **N** | **%** | **N** | **%** | **N** | **%** |
| **Complete Sample** | - | - | 1131 | 49.0 | 1019 | 43.4 | 1121 | 48.4 | 980 | 42.1 | 1136 | 48.8 |
| **Sociodemographic** | | | | | | | | | | | | |
| <60 Years of Age | 1496 | 63.4 | 740 | 50.4 | 640 | 42.9 | 735 | 49.9 | 633 | 42.9 | 749 | 50.6 |
| ≥60 Years of Age | 863 | 36.6 | 391 | 46.6 | 379 | 44.2 | 386 | 45.8 | 347 | 40.8 | 387 | 45.7 |
| White Ethnicity | 2314 | 98.4 | 1109 | 49.0 | 1003 | 43.5 | 1099 | 48.4 | 959 | 42.0 | 1113 | 48.8 |
| Non-White Ethnicity | 37 | 1.6 | 19 | 51.4 | 11 | 29.7 | 19 | 51.4 | 17 | 46.0 | 19 | 51.4 |
| No University Education | 1788 | 75.8 | 911 | 52.2 | 748 | 42.0 | 915 | 52.2 | 800 | 45.4 | 902 | 51.2 |
| University Education | 579 | 24.2 | 220 | 39.3 | 271 | 47.7 | 206 | 36.6 | 180 | 31.9 | 234 | 41.3 |
| Not a Key Worker | 1550 | 65.7 | 733 | 48.3 | 666 | 34.2 | 726 | 47.7 | 647 | 42.3 | 736 | 48.1 |
| Key Worker | 809 | 34.3 | 398 | 50.4 | 353 | 43.7 | 395 | 49.9 | 333 | 41.7 | 400 | 50.1 |
| Not a Healthcare Worker | 1965 | 82.9 | 938 | 49.1 | 834 | 42.8 | 934 | 48.7 | 827 | 42.9 | 940 | 48.7 |
| Healthcare Worker | 403 | 17.1 | 193 | 48.9 | 185 | 46.0 | 187 | 47.2 | 153 | 38.3 | 196 | 49.5 |
| **Current Health** | | | | | | | | | | | | |
| No Probable Generalised Anxiety | 2085 | 90.3 | 950 | 46.6 | 874 | 42.1 | 937 | 45.8 | 812 | 39.4 | 955 | 46.4 |
| Probable Generalised Anxiety | 223 | 9.7 | 162 | 74.0 | 124 | 55.6 | 165 | 75.3 | 152 | 69.1 | 163 | 73.8 |
| No Likely Depression | 2135 | 94.6 | 1012 | 48.4 | 925 | 43.5 | 999 | 47.6 | 876 | 41.5 | 1013 | 48.1 |
| Likely Depression | 121 | 5.4 | 77 | 65.3 | 58 | 48.3 | 79 | 67.0 | 68 | 57.6 | 78 | 65.0 |
| No Low Mental Wellbeing | 1871 | 82.4 | 833 | 45.5 | 788 | 42.3 | 822 | 44.7 | 729 | 39.5 | 846 | 45.8 |
| Low Mental Wellbeing | 400 | 17.6 | 258 | 66.2 | 202 | 50.8 | 260 | 66.3 | 216 | 54.8 | 252 | 63.6 |
| No Medical Conditions | 960 | 50.8 | 402 | 42.7 | 395 | 41.3 | 385 | 40.8 | 323 | 34.1 | 425 | 44.8 |
| ≥1 Medical Conditions | 929 | 49.2 | 484 | 53.5 | 415 | 44.9 | 484 | 53.3 | 444 | 48.4 | 473 | 51.8 |
| Not Self-Isolating as Not Vulnerable | 2193 | 94.1 | 1019 | 47.5 | 927 | 42.5 | 1006 | 46.8 | 875 | 40.4 | 1030 | 47.6 |
| Self-Isolating as Vulnerable | 137 | 5.9 | 95 | 70.9 | 75 | 55.2 | 98 | 72.6 | 89 | 65.4 | 88 | 65.2 |
| Not Live with Vulnerable Person | 2053 | 88.5 | 977 | 48.6 | 891 | 43.6 | 970 | 48.1 | 843 | 41.6 | 980 | 48.4 |
| Live with Vulnerable Person | 268 | 11.6 | 132 | 50.4 | 112 | 42.1 | 129 | 48.9 | 115 | 43.2 | 138 | 52.3 |
| No Suspected COVID-19 (Self-Report) | 2052 | 87.2 | 982 | 48.7 | 873 | 42.7 | 974 | 48.2 | 856 | 42.1 | 969 | 47.8 |
| Suspected COVID-19 (Self-Report) | 302 | 12.8 | 147 | 51.4 | 144 | 48.0 | 146 | 50.7 | 122 | 42.1 | 165 | 55.9 |
| No Suspected COVID-19 (Algorithm) | 2315 | 98.1 | 1109 | 49.0 | 995 | 43.2 | 1097 | 48.3 | 959 | 42.0 | 1112 | 48.7 |
| Suspected COVID-19 (Algorithm) | 44 | 1.9 | 22 | 52.4 | 24 | 55.8 | 24 | 55.8 | 21 | 48.8 | 24 | 55.8 |
| **Pre-pandemic Health** | | | | | | | | | | | | |
| No Anxiety | 1598 | 75.0 | 740 | 47.3 | 686 | 43.1 | 725 | 46.2 | 635 | 40.2 | 739 | 46.9 |
| Anxiety | 533 | 25.0 | 280 | 53.6 | 245 | 46.3 | 281 | 53.4 | 242 | 46.2 | 294 | 55.7 |
| No Depression | 1691 | 79.3 | 802 | 48.5 | 742 | 44.1 | 792 | 47.8 | 698 | 41.8 | 806 | 48.4 |
| Depression | 442 | 20.7 | 218 | 50.0 | 191 | 43.4 | 214 | 48.9 | 179 | 41.0 | 229 | 52.1 |
| No Low Wellbeing | 1466 | 75.0 | 655 | 45.7 | 610 | 41.8 | 640 | 44.5 | 553 | 38.2 | 660 | 45.6 |
| Low Wellbeing | 490 | 25.0 | 261 | 54.3 | 236 | 48.4 | 265 | 54.9 | 220 | 45.5 | 272 | 56.0 |
| **Current Risk Behaviours** | | | | | | | | | | | | |
| Low-Risk Drinking | 1542 | 66.4 | 746 | 49.5 | 653 | 42.5 | 746 | 49.3 | 647 | 42.5 | 745 | 49.1 |
| High-Risk Drinking | 781 | 33.6 | 364 | 47.5 | 351 | 45.1 | 353 | 46.0 | 313 | 40.6 | 369 | 47.7 |
| No Increased Alcohol Use | 1130 | 56.6 | 480 | 43.5 | 475 | 42.2 | 480 | 43.4 | 422 | 37.9 | 494 | 44.4 |
| Increased Alcohol Use | 867 | 43.4 | 461 | 54.0 | 388 | 44.9 | 449 | 52.5 | 388 | 45.1 | 453 | 52.7 |
| No Increased Smoking/E-Cigarette Use | 182 | 65.0 | 88 | 49.2 | 78 | 42.9 | 86 | 48.0 | 86 | 47.5 | 83 | 46.1 |
| Increased Smoking/E-Cigarette Use | 98 | 35.0 | 61 | 63.5 | 47 | 48.0 | 55 | 57.3 | 54 | 56.3 | 55 | 56.1 |
| No Self-Isolating Given COVID-19 | 208 | 70.3 | 97 | 48.5 | 93 | 44.7 | 97 | 48.5 | 83 | 40.9 | 110 | 54.2 |
| Self-Isolating Given COVID-19 | 88 | 29.7 | 45 | 56.3 | 49 | 57.0 | 45 | 54.9 | 35 | 43.2 | 51 | 59.3 |
| No Face-To-Face Contact | 604 | 25.8 | 316 | 53.7 | 268 | 44.6 | 315 | 53.3 | 277 | 46.5 | 305 | 51.4 |
| ≥1 Face-To-Face Contact | 1735 | 74.2 | 804 | 47.4 | 743 | 43.0 | 795 | 46.7 | 692 | 40.4 | 822 | 48.0 |
| No Physical Contact | 1623 | 75.1 | 792 | 49.9 | 723 | 44.7 | 790 | 49.6 | 690 | 43.1 | 800 | 49.9 |
| ≥1 Physical Contact | 537 | 24.9 | 241 | 45.8 | 215 | 40.3 | 239 | 45.3 | 204 | 38.4 | 242 | 45.7 |
| **Pre-pandemic Risk Behaviours** | | | | | | | | | | | | |
| Low-Risk Drinking | 1239 | 70.5 | 577 | 47.6 | 539 | 43.7 | 571 | 46.9 | 473 | 38.6 | 587 | 48.1 |
| High-Risk Drinking | 518 | 29.5 | 237 | 46.6 | 226 | 43.7 | 231 | 45.3 | 212 | 41.5 | 245 | 47.5 |
| No Smoking | 1811 | 93.0 | 827 | 46.6 | 774 | 42.9 | 821 | 46.1 | 695 | 38.8 | 845 | 47.2 |
| Smoking | 137 | 7.0 | 76 | 57.1 | 66 | 48.5 | 74 | 55.2 | 72 | 53.7 | 74 | 54.8 |
| **Early Pandemic Risk Behaviours** | | | | | | | | | | | | |
| No Self-Isolating Given COVID-19 | 175 | 68.9 | 85 | 50.9 | 77 | 44.0 | 84 | 50.3 | 73 | 42.7 | 94 | 55.6 |
| Self-Isolating Given COVID-19 | 79 | 31.3 | 37 | 51.4 | 40 | 51.3 | 39 | 53.4 | 32 | 43.8 | 43 | 55.8 |
| No Face-To-Face Contact | 632 | 32.5 | 306 | 49.6 | 280 | 44.6 | 297 | 47.9 | 263 | 42.2 | 307 | 49.3 |
| ≥1 Face-To-Face Contact | 1311 | 67.5 | 608 | 47.5 | 561 | 42.9 | 598 | 46.6 | 519 | 40.2 | 617 | 47.9 |
| No Physical Contact | 1441 | 84.4 | 688 | 48.8 | 631 | 44.0 | 675 | 47.7 | 594 | 41.9 | 695 | 48.8 |
| ≥1 Physical Contact | 266 | 15.6 | 116 | 44.8 | 107 | 40.4 | 118 | 45.4 | 100 | 37.9 | 121 | 46.5 |

*Note.* Complete sample = participants who completed ALSPAC’s second COVID-19 questionnaire. Complete case = participants who had complete data on the covariates included in the partially adjusted regression models (i.e., age, education, and keyworker status).

**Supplementary Table S7**

*Complete Case Frequencies and Percentages of COVID-19 Risk Perceptions by Sociodemographic, Health, and Risk Behaviour Variables (Young People)*

|  | **COVID-19 Risk Perceptions (High Only)** | | | | | | | | | | | |
| --- | --- | --- | --- | --- | --- | --- | --- | --- | --- | --- | --- | --- |
|  | **Complete Sample** | | **Holistic** | | **Cognitive** | | **Affective** | | **Self** | | **Other** | |
| **Participant Characteristic** | **N** | **%** | **N** | **%** | **N** | **%** | **N** | **%** | **N** | **%** | **N** | **%** |
| **Complete Sample** | - | - | 973 | 48.3 | 474 | 23.2 | 909 | 45.0 | 787 | 38.6 | 791 | 39.1 |
| **Sociodemographic** | | | | | | | | | | | | |
| Female | 1470 | 71.7 | 786 | 54.4 | 358 | 24.4 | 741 | 51.2 | 635 | 43.4 | 638 | 44.0 |
| Male | 579 | 28.3 | 188 | 32.9 | 116 | 20.1 | 168 | 29.4 | 152 | 26.3 | 153 | 26.8 |
| White Ethnicity | 1790 | 96.7 | 834 | 47.4 | 419 | 23.5 | 776 | 44.0 | 678 | 38.1 | 677 | 38.4 |
| Non-White Ethnicity | 61 | 3.3 | 36 | 59.0 | 16 | 26.2 | 31 | 50.8 | 23 | 37.7 | 29 | 47.5 |
| No University Education | 543 | 26.5 | 292 | 55.3 | 84 | 15.5 | 296 | 56.1 | 260 | 48.4 | 223 | 41.9 |
| University Education | 1506 | 73.5 | 681 | 45.8 | 390 | 26.0 | 613 | 41.1 | 527 | 35.1 | 568 | 38.1 |
| Not a Key Worker | 1250 | 61.0 | 576 | 46.9 | 273 | 21.9 | 537 | 43.6 | 460 | 37.0 | 479 | 38.9 |
| Key Worker | 799 | 39.0 | 397 | 50.4 | 201 | 25.2 | 372 | 47.3 | 327 | 41.1 | 312 | 39.4 |
| Not a Healthcare Worker | 1742 | 85.0 | 806 | 47.1 | 391 | 22.5 | 757 | 44.1 | 656 | 37.9 | 656 | 38.2 |
| Healthcare Worker | 307 | 15.0 | 167 | 55.1 | 83 | 27.0 | 152 | 50.2 | 131 | 42.8 | 135 | 44.4 |
| **Current Health** | | | | | | | | | | | | |
| No Probable Generalised Anxiety | 1570 | 77.8 | 648 | 42.1 | 342 | 21.8 | 591 | 38.3 | 516 | 33.0 | 513 | 33.2 |
| Probable Generalised Anxiety | 448 | 22.2 | 311 | 69.7 | 127 | 28.4 | 302 | 67.7 | 260 | 58.2 | 267 | 59.7 |
| No Likely Depression | 1680 | 83.0 | 742 | 44.9 | 375 | 22.4 | 687 | 41.5 | 595 | 35.5 | 595 | 35.9 |
| Likely Depression | 343 | 17.0 | 215 | 63.6 | 88 | 25.7 | 206 | 61.0 | 178 | 52.4 | 184 | 54.3 |
| No Low Mental Wellbeing | 1040 | 69.4 | 611 | 44.2 | 316 | 22.6 | 560 | 40.5 | 484 | 34.6 | 498 | 35.9 |
| Low Mental Wellbeing | 620 | 30.6 | 350 | 57.4 | 153 | 24.7 | 337 | 55.3 | 294 | 47.7 | 281 | 46.0 |
| No Medical Conditions | 1117 | 66.9 | 480 | 43.8 | 250 | 22.4 | 437 | 39.8 | 363 | 32.6 | 397 | 36.2 |
| ≥1 Medical Conditions | 553 | 33.1 | 304 | 55.3 | 129 | 23.3 | 293 | 53.3 | 250 | 45.4 | 242 | 43.8 |
| Not Self-Isolating as Not Vulnerable | 1951 | 96.1 | 903 | 47.1 | 448 | 23.0 | 839 | 43.7 | 717 | 36.9 | 737 | 38.3 |
| Self-Isolating as Vulnerable | 80 | 3.9 | 59 | 74.7 | 22 | 27.5 | 85 | 54.1 | 58 | 72.5 | 44 | 55.7 |
| Not Live with Vulnerable Person | 1878 | 92.2 | 879 | 47.6 | 444 | 23.7 | 821 | 44.4 | 714 | 38.2 | 713 | 38.5 |
| Live with Vulnerable Person | 159 | 7.8 | 89 | 56.7 | 28 | 17.6 | 59 | 73.4 | 71 | 44.9 | 73 | 46.2 |
| No Suspected COVID-19 (Self-Report) | 1684 | 82.2 | 799 | 48.3 | 366 | 21.8 | 763 | 46.1 | 663 | 39.6 | 645 | 38.9 |
| Suspected COVID-19 (Self-Report) | 364 | 17.8 | 174 | 48.2 | 108 | 29.7 | 146 | 40.4 | 124 | 34.3 | 146 | 40.2 |
| No Suspected COVID-19 (Algorithm) | 1973 | 96.3 | 928 | 47.8 | 455 | 23.1 | 864 | 44.5 | 750 | 38.2 | 754 | 38.8 |
| Suspected COVID-19 (Algorithm) | 76 | 3.7 | 45 | 60.0 | 19 | 25.0 | 45 | 60.0 | 37 | 49.3 | 37 | 48.7 |
| **Pre-pandemic Health** | | | | | | | | | | | | |
| No Anxiety | 1459 | 90.5 | 652 | 45.4 | 330 | 22.7 | 597 | 41.5 | 520 | 35.8 | 529 | 36.7 |
| Anxiety | 154 | 9.6 | 95 | 62.9 | 49 | 31.8 | 90 | 59.6 | 74 | 48.4 | 82 | 54.0 |
| No Depression | 1453 | 89.8 | 651 | 45.7 | 332 | 22.9 | 595 | 41.7 | 513 | 35.5 | 537 | 37.5 |
| Depression | 165 | 10.2 | 97 | 58.8 | 48 | 29.1 | 92 | 55.8 | 81 | 49.1 | 75 | 45.5 |
| No Low Wellbeing | 1412 | 82.5 | 634 | 45.7 | 317 | 22.5 | 586 | 42.2 | 516 | 36.7 | 524 | 37.7 |
| Low Wellbeing | 300 | 17.5 | 172 | 58.1 | 77 | 25.7 | 161 | 54.4 | 141 | 47.2 | 137 | 46.3 |
| **Current Risk Behaviours** | | | | | | | | | | | | |
| Low-Risk Drinking | 1231 | 60.1 | 610 | 50.5 | 275 | 22.4 | 586 | 48.4 | 502 | 41.0 | 493 | 40.6 |
| High-Risk Drinking | 817 | 39.9 | 362 | 44.9 | 198 | 24.2 | 323 | 40.1 | 285 | 35.1 | 297 | 36.8 |
| No Increased Alcohol Use | 987 | 55.8 | 435 | 44.7 | 217 | 22.0 | 402 | 41.3 | 337 | 34.2 | 371 | 38.0 |
| Increased Alcohol Use | 781 | 44.2 | 385 | 50.2 | 196 | 25.1 | 359 | 46.8 | 310 | 40.0 | 305 | 39.6 |
| No Increased Smoking/E-Cigarette Use | 261 | 56.6 | 111 | 43.7 | 47 | 18.0 | 111 | 43.7 | 97 | 37.3 | 99 | 39.0 |
| Increased Smoking/E-Cigarette Use | 200 | 43.4 | 106 | 53.8 | 45 | 22.5 | 98 | 49.8 | 90 | 45.2 | 89 | 45.0 |
| No Self-Isolating Given COVID-19 | 210 | 58.2 | 91 | 43.5 | 51 | 24.3 | 81 | 38.8 | 74 | 35.2 | 74 | 35.4 |
| Self-Isolating Given COVID-19 | 151 | 41.8 | 81 | 54.4 | 57 | 37.8 | 64 | 43.0 | 48 | 32.2 | 71 | 47.0 |
| No Face-To-Face Contact | 874 | 42.8 | 431 | 50.1 | 205 | 23.5 | 414 | 48.0 | 348 | 40.0 | 358 | 41.4 |
| ≥1 Face-To-Face Contact | 1166 | 57.2 | 538 | 47.0 | 266 | 22.9 | 491 | 42.8 | 434 | 37.5 | 432 | 37.6 |
| No Physical Contact | 1552 | 79.0 | 742 | 48.7 | 366 | 23.6 | 689 | 45.2 | 603 | 39.1 | 596 | 39.0 |
| ≥1 Physical Contact | 413 | 21.0 | 185 | 45.2 | 88 | 21.3 | 176 | 43.0 | 149 | 36.3 | 151 | 36.7 |
| **Pre-pandemic Risk Behaviours** | | | | | | | | | | | | |
| Low-Risk Drinking | 730 | 44.6 | 360 | 50.0 | 162 | 22.3 | 346 | 48.0 | 310 | 42.8 | 290 | 40.1 |
| High-Risk Drinking | 906 | 55.4 | 400 | 44.9 | 217 | 24.0 | 368 | 41.3 | 308 | 34.1 | 331 | 37.2 |
| No Smoking | 1046 | 78.6 | 671 | 48.5 | 330 | 23.5 | 615 | 44.3 | 537 | 38.4 | 547 | 39.4 |
| Smoking | 383 | 21.4 | 190 | 50.3 | 89 | 23.2 | 185 | 48.9 | 157 | 41.1 | 160 | 42.3 |
| No E-Cigarette Use | 1725 | 96.4 | 824 | 48.5 | 406 | 23.6 | 763 | 44.8 | 668 | 38.9 | 680 | 39.9 |
| E-Cigarette Use | 64 | 3.6 | 37 | 57.8 | 13 | 20.3 | 37 | 57.8 | 26 | 40.6 | 27 | 42.2 |
| **Early Pandemic Risk Behaviours** | | | | | | | | | | | | |
| No Self-Isolating Given COVID-19 | 113 | 100.0 | 50 | 44.3 | 29 | 25.7 | 43 | 38.1 | 36 | 31.9 | 40 | 35.4 |
| Self-Isolating Given COVID-19 | 0 | 0 | 0 | 0 | 0 | 0 | 0 | 0 | 0 | 0 | 0 | 0 |
| No Face-To-Face Contact | 752 | 45.0 | 367 | 49.3 | 183 | 24.4 | 336 | 45.1 | 293 | 39.0 | 304 | 40.9 |
| ≥1 Face-To-Face Contact | 920 | 55.0 | 419 | 46.4 | 195 | 21.2 | 394 | 43.5 | 323 | 35.3 | 338 | 37.2 |
| No Physical Contact | 1261 | 83.2 | 599 | 48.1 | 302 | 24.0 | 547 | 43.9 | 471 | 37.4 | 502 | 40.2 |
| ≥1 Physical Contact | 155 | 16.8 | 120 | 47.6 | 53 | 20.8 | 117 | 46.4 | 98 | 38.6 | 93 | 36.8 |

*Note.* Complete sample = participants who completed ALSPAC’s second COVID-19 questionnaire. Complete case = participants who had complete data on the covariates included in the partially adjusted regression models (i.e., age, gender, education, and keyworker status).

**Supplementary Table S8**

*Cross-Sectional Associations between COVID-19 Risk Perceptions and Mental Health, Wellbeing, and Risk Behaviours (Mothers)*

|  | **COVID-19 Risk Perceptions (Exposures)** | | | | | | | | | | | | | | |
| --- | --- | --- | --- | --- | --- | --- | --- | --- | --- | --- | --- | --- | --- | --- | --- |
|  | **Holistic** | | | **Cognitive** | | | **Affective** | | | **Self** | | | **Other** | | |
| **Outcome and Model** | **OR**  **(95% CI)** | **P** | **N** | **OR**  **(95% CI)** | **P** | **N** | **OR**  **(95% CI)** | **P** | **N** | **OR**  **(95% CI)** | **P** | **N** | **OR**  **(95% CI)** | **P** | **N** |
| **Generalised Anxiety Disorder** | | | | | | | | | | | | | | | |
| Unadjusted | 3.47  (2.56, 4.71) | <.001 | 2456 | 1.71  (1.32, 2.23) | <.001 | 2500 | 3.86  (2.83, 5.26) | <.001 | 2464 | 3.68  (2.75, 4.92) | <.001 | 2477 | 3.47  (2.56, 4.70) | <.001 | 2479 |
| Partially Adjusted | 3.17  (2.31, 4.34) | <.001 | 2258 | 1.77  (1.34, 2.34) | <.001 | 2299 | 3.51  (2.54, 4.84) | <.001 | 2266 | 3.35  (2.48, 4.52) | <.001 | 2279 | 3.15  (2.31, 4.31) | <.001 | 2278 |
| Fully Adjusted | 3.42  (2.28, 5.14) | <.001 | 1553 | 1.72  (1.21, 2.47) | .003 | 1578 | 3.68  (2.45, 5.55) | <.001 | 1558 | 3.54  (2.42, 5.19) | <.001 | 1567 | 3.08  (2.06, 4.60) | <.001 | 1566 |
| **Depression** | | | | | | | | | | | | | | | |
| Unadjusted | 2.09  (1.44, 3.04) | <.001 | 2401 | 1.25  (0.88, 1.77) | .214 | 2445 | 2.32  (1.59, 3.39) | <.001 | 2408 | 1.97  (1.37, 2.82) | <.001 | 2421 | 2.10  (1.45, 3.05) | <.001 | 2423 |
| Partially Adjusted | 1.86  (1.26, 2.75) | .002 | 2208 | 1.25  (0.87, 1.81) | .232 | 2248 | 2.04  (1.37, 3.03) | <.001 | 2215 | 1.77  (1.21, 2.58) | .003 | 2228 | 1.89  (1.28, 2.78) | .001 | 2227 |
| Fully Adjusted | 1.75  (1.06, 2.89) | .029 | 1523 | 1.14  (0.70, 1.84) | .596 | 1547 | 1.91  (1.15, 3.17) | .012 | 1528 | 1.64  (1.01, 2.66) | .046 | 1536 | 1.52  (0.93, 2.49) | .098 | 1536 |
| **Low Wellbeing** | | | | | | | | | | | | | | | |
| Unadjusted | 2.38  (1.91, 2.96) | <.001 | 2414 | 1.40  (1.14, 1.71) | .001 | 2457 | 2.51  (2.02, 3.13) | <.001 | 2442 | 1.92  (1.56, 2.36) | <.001 | 2434 | 2.15  (1.74, 2.66) | <.001 | 2437 |
| Partially Adjusted | 2.25  (1.79, 2.83) | <.001 | 2222 | 1.45  (1.17, 1.80) | .001 | 2262 | 2.32  (1.84, 2.92) | <.001 | 2230 | 1.78  (1.43, 2.22) | <.001 | 2242 | 1.99  (1.59, 2.50) | <.001 | 2242 |
| Fully Adjusted | 2.06  (1.55, 2.76) | <.001 | 1532 | 1.28  (0.97, 1.69) | .076 | 1556 | 2.31  (1.73, 3.10) | <.001 | 1537 | 1.79  (1.35, 2.37) | <.001 | 1545 | 1.71  (1.28, 2.26) | <.001 | 1545 |
| **High-Risk Drinking** | | | | | | | | | | | | | | | |
| Unadjusted | 0.94  (0.80, 1.11) | .479 | 2469 | 1.13  (0.96, 1.34) | .142 | 2514 | 0.91  (0.77, 1.07) | .247 | 2476 | 0.93  (0.78, 1.10) | .389 | 2489 | 0.95  (0.80, 1.12) | .518 | 2491 |
| Partially Adjusted | 0.92  (0.78, 1.10) | .382 | 2273 | 1.11  (0.93, 1.32) | .247 | 2314 | 0.88  (0.73, 1.04) | .140 | 2280 | 0.92  (0.77, 1.10) | .381 | 2293 | 0.95  (0.80, 1.13) | .552 | 2292 |
| Fully Adjusted | 0.98  (0.77, 1.25) | .877 | 1563 | 1.17  (0.92, 1.48) | .208 | 1587 | 0.92  (0.72, 1.17) | .482 | 1568 | 0.91  (0.71, 1.17) | .473 | 1576 | 0.88  (0.69, 1.13) | .321 | 1576 |
| **Increased Alcohol Use** | | | | | | | | | | | | | | | |
| Unadjusted | 1.47  (1.24, 1.75) | <.001 | 2122 | 1.09  (0.92, 1.30) | .300 | 2158 | 1.43  (1.20, 1.70) | <.001 | 2128 | 1.32  (1.11, 1.57) | .002 | 2139 | 1.36  (1.14, 1.61) | .001 | 2139 |
| Partially Adjusted | 1.51  (1.26, 1.81) | <.001 | 1957 | 1.12  (0.94, 1.34) | .207 | 1990 | 1.43  (1.19, 1.71) | <.001 | 1963 | 1.33  (1.11, 1.60) | .002 | 1974 | 1.39  (1.16, 1.66) | <.001 | 1972 |
| Fully Adjusted | 1.77  (1.43, 2.19) | <.001 | 1449 | 1.15  (0.93, 1.42) | .192 | 1471 | 1.63  (1.32, 2.02) | <.001 | 1452 | 1.48  (1.19, 1.83) | <.001 | 1460 | 1.52  (1.23, 1.88) | <.001 | 1460 |
| **Increased Smoking/E-Cigarette Use** | | | | | | | | | | | | | | | |
| Unadjusted | 1.89  (1.17, 3.06) | .010 | 308 | 1.16  (0.73, 1.84) | .533 | 314 | 1.62  (1.01, 2.60) | .046 | 308 | 1.50  (0.94, 2.40) | .092 | 310 | 1.59  (0.99, 2.53) | .053 | 312 |
| Partially Adjusted | 1.78  (1.06, 3.01) | .030 | 275 | 1.25  (0.76, 2.08) | .380 | 280 | 1.38  (0.82, 2.31) | .220 | 275 | 1.36  (0.82, 2.27) | .234 | 277 | 1.42  (0.86, 2.36) | .171 | 278 |
| Fully Adjusted | 1.88  (0.90, 3.90) | .092 | 177 | 1.09  (0.55, 2.15) | .806 | 182 | 1.98  (0.95, 4.14) | .068 | 177 | 1.37  (0.67, 2.79) | .391 | 179 | 1.41  (0.71, 2.82) | .328 | 180 |
| **Self-Isolating Given Suspected COVID-19 Infection** | | | | | | | | | | | | | | | |
| Unadjusted | 1.37  (0.83, 2.25) | .219 | 301 | 1.51  (0.93, 2.45) | .097 | 315 | 1.35  (0.82, 2.21) | .234 | 303 | 1.03  (0.63, 1.71) | .893 | 305 | 1.24  (0.76, 2.02) | .392 | 310 |
| Partially Adjusted | 1.40  (0.82, 2.37) | .219 | 280 | 1.68  (1.00, 2.83) | .050 | 294 | 1.33  (0.78, 2.26) | .290 | 282 | 1.18  (0.69, 2.01) | .540 | 284 | 1.28  (0.76, 2.15) | .360 | 289 |
| Fully Adjusted | 1.72  (0.88, 3.34) | .111 | 195 | 1.70  (0.90, 3.21) | .105 | 203 | 1.43  (0.74, 2.76) | .284 | 197 | 0.96  (0.49, 1.89) | .912 | 198 | 1.54  (0.80, 2.99) | .197 | 202 |
| **Face-To-Face Contact Outside Household** | | | | | | | | | | | | | | | |
| Unadjusted | 0.77  (0.65, 0.93) | .006 | 2483 | 0.92  (0.77, 1.10) | .375 | 2529 | 0.77  (0.64, 0.92) | .005 | 2491 | 0.75  (0.63, 0.90) | .002 | 2504 | 0.88  (0.73, 1.05) | .154 | 2506 |
| Partially Adjusted | 0.77  (0.63, 0.93) | .006 | 2287 | 0.93  (0.77, 1.13) | .471 | 2329 | 0.75  (0.62, 0.91) | .003 | 2295 | 0.78  (0.64, 0.94) | .010 | 2308 | 0.87  (0.72, 1.05) | .134 | 2307 |
| Fully Adjusted | 0.76  (0.60, 0.96) | .023 | 1574 | 0.93  (0.74, 1.17) | .531 | 1599 | 0.72  (0.57, 0.91) | .006 | 1579 | 0.80  (0.63, 1.01) | .066 | 1588 | 0.84  (0.66, 1.06) | .135 | 1587 |
| **Physical Contact Outside Household** | | | | | | | | | | | | | | | |
| Unadjusted | 0.83  (0.69, 1.01) | .058 | 2286 | 0.83  (0.69, 1.01) | .061 | 2325 | 0.83  (0.69, 1.01) | .062 | 2294 | 0.82  (0.67, 0.99) | .041 | 2304 | 0.83  (0.68, 1.00) | .049 | 2307 |
| Partially Adjusted | 0.84  (0.68, 1.02) | .081 | 2114 | 0.83  (0.68, 1.02) | .071 | 2150 | 0.83  (0.68, 1.01) | .064 | 2122 | 0.82  (0.67, 1.01) | .056 | 2132 | 0.83  (0.68, 1.01) | .070 | 2133 |
| Fully Adjusted | 0.82  (0.64, 1.04) | .100 | 1459 | 0.82  (0.65, 1.04) | .107 | 1481 | 0.77  (0.60, 0.98) | .033 | 1464 | 0.82  (0.64, 1.05) | .115 | 1471 | 0.81  (0.64, 1.03) | .088 | 1471 |

*Note.* Logistic regressions. OR = odds ratio. CI = confidence interval. Partially adjusted = adjusted for sociodemographic variables (age, education, and keyworker status). Fully adjusted = additionally adjusted for prior mental health and risk behaviour variables (anxiety, depression, high-risk drinking, smoking, and suspected COVID-19 infection). All variables in the models are binary. All risk perception variables were dichotomised at the median.

**Supplementary Table S9**

*Cross-Sectional Associations between COVID-19 Risk Perceptions and Mental Health, Wellbeing, and Risk Behaviours (Young People)*

|  | **COVID-19 Risk Perceptions (Exposures)** | | | | | | | | | | | | | | |
| --- | --- | --- | --- | --- | --- | --- | --- | --- | --- | --- | --- | --- | --- | --- | --- |
|  | **Holistic** | | | **Cognitive** | | | **Affective** | | | **Self** | | | **Other** | | |
| **Outcome and Model** | **OR**  **(95% CI)** | **P** | **N** | **OR**  **(95% CI)** | **P** | **N** | **OR**  **(95% CI)** | **P** | **N** | **OR**  **(95% CI)** | **P** | **N** | **OR**  **(95% CI)** | **P** | **N** |
| **Generalised Anxiety Disorder** | | | | | | | | | | | | | | | |
| Unadjusted | 2.90  (2.39, 3.52) | <.001 | 2526 | 1.51  (1.23, 1.86) | <.001 | 2565 | 3.10  (2.55, 3.75) | <.001 | 2530 | 2.65  (2.20, 3.19) | <.001 | 2561 | 2.70  (2.24, 3.26) | <.001 | 2532 |
| Partially Adjusted | 2.88  (2.29, 3.63) | <.001 | 1986 | 1.48  (1.16, 1.89) | .002 | 2015 | 3.06  (2.43, 3.84) | <.001 | 1989 | 2.58  (2.07, 3.22) | <.001 | 2010 | 2.76  (2.21, 3.44) | <.001 | 1992 |
| Fully Adjusted | 2.27  (1.66, 3.12) | <.001 | 1227 | 1.45  (1.03, 2.05) | .034 | 1242 | 2.37  (1.73, 3.24) | <.001 | 1229 | 2.21  (1.62, 3.01) | <.001 | 1239 | 2.28  (1.67, 3.10) | <.001 | 1230 |
| **Depression** | | | | | | | | | | | | | | | |
| Unadjusted | 2.05  (1.67, 2.52) | <.001 | 2521 | 1.40  (1.12, 1.75) | .003 | 2561 | 2.13  (1.74, 2.62) | <.001 | 2525 | 1.90  (1.56, 2.33) | <.001 | 2556 | 2.00  (1.63, 2.44) | <.001 | 2528 |
| Partially Adjusted | 1.95  (1.52, 2.50) | <.001 | 1990 | 1.25  (0.95, 1.65) | .105 | 2020 | 1.97  (1.54, 2.52) | <.001 | 1993 | 1.80  (1.41, 2.29) | <.001 | 2014 | 1.97  (1.55, 2.51) | <.001 | 1997 |
| Fully Adjusted | 1.79  (1.26, 2.55) | .001 | 1230 | 1.13  (0.76, 1.68) | .555 | 1246 | 1.74  (1.23, 2.47) | .002 | 1232 | 1.49  (1.05, 2.12) | .025 | 1242 | 1.65  (1.17, 2.34) | .004 | 1234 |
| **Low Wellbeing** | | | | | | | | | | | | | | | |
| Unadjusted | 1.70  (1.43, 2.01) | <.001 | 2528 | 1.21  (1.00, 1.47) | .049 | 2568 | 1.83  (1.55, 2.17) | <.001 | 2532 | 1.71  (1.45, 2.03) | <.001 | 2563 | 1.47  (1.24, 1.74) | <.001 | 2535 |
| Partially Adjusted | 1.63  (1.34, 1.99) | <.001 | 1991 | 1.19  (0.95, 1.49) | .136 | 2021 | 1.73  (1.42, 2.11) | <.001 | 1994 | 1.63  (1.34, 1.99) | <.001 | 2015 | 1.47  (1.21, 1.79) | <.001 | 1998 |
| Fully Adjusted | 1.60  (1.23, 2.08) | <.001 | 1234 | 1.15  (0.85, 1.55) | .369 | 1250 | 1.54  (1.18, 2.01) | .001 | 1236 | 1.48  (1.13, 1.93) | .004 | 1246 | 1.45  (1.11, 1.89) | .006 | 1238 |
| **High-Risk Drinking** | | | | | | | | | | | | | | | |
| Unadjusted | 0.75  (0.64, 0.88) | <.001 | 2553 | 1.09  (0.90, 1.31) | .371 | 2593 | 0.68  (0.58, 0.80) | <.001 | 2558 | 0.70  (0.60, 0.82) | <.001 | 2588 | 0.82  (0.70, 0.96) | .016 | 2560 |
| Partially Adjusted | 0.88  (0.73, 1.06) | .184 | 2014 | 1.09  (0.88, 1.35) | .423 | 2044 | 0.80  (0.66, 0.96) | .017 | 2017 | 0.87  (0.72, 1.05) | .138 | 2038 | 0.91  (0.75, 1.10) | .325 | 2021 |
| Fully Adjusted | 1.01  (0.78, 1.30) | .969 | 1246 | 1.19  (0.89, 1.59) | .240 | 1262 | 0.82  (0.63, 1.06) | .132 | 1248 | 0.96  (0.74, 1.25) | .745 | 1258 | 0.94  (0.73, 1.22) | .642 | 1250 |
| **Increased Alcohol Use** | | | | | | | | | | | | | | | |
| Unadjusted | 1.11  (0.94, 1.31) | .229 | 2212 | 1.12  (0.92, 1.36) | .259 | 2247 | 1.17  (0.98, 1.38) | .076 | 2215 | 1.16  (0.98, 1.38) | .088 | 2240 | 1.05  (0.86, 1.25) | .572 | 2218 |
| Partially Adjusted | 1.26  (1.04, 1.53) | .019 | 1741 | 1.18  (0.95, 1.48) | .142 | 1768 | 1.27  (1.04, 1.55) | .017 | 1741 | 1.30  (1.07, 1.59) | .009 | 1760 | 1.07  (0.88, 1.30) | .525 | 1747 |
| Fully Adjusted | 1.18  (0.92, 1.51) | .194 | 1092 | 1.13  (0.85, 1.50) | .401 | 1107 | 1.27  (0.99, 1.64) | .060 | 1092 | 1.29  (1.00, 1.67) | .053 | 1101 | 0.96  (0.75, 1.24) | .750 | 1096 |
| **Increased Smoking/E-Cigarette Use** | | | | | | | | | | | | | | | |
| Unadjusted | 1.27  (0.93, 1.74) | .135 | 633 | 1.27  (0.86, 1.85) | .226 | 649 | 1.18  (0.86, 1.62) | .295 | 634 | 1.20  (0.88, 1.65) | .256 | 647 | 1.24  (0.91, 1.71) | .177 | 634 |
| Partially Adjusted | 1.36  (0.92, 2.00) | .125 | 451 | 1.34  (0.84, 2.15) | .220 | 461 | 1.11  (0.75, 1.64) | .608 | 451 | 1.26  (0.85, 1.86) | .245 | 459 | 1.16  (0.79, 1.71) | .457 | 452 |
| Fully Adjusted | 1.08  (0.61, 1.92) | .787 | 243 | 1.06  (0.53, 2.13) | .877 | 244 | 0.62  (0.34, 1.12) | .111 | 243 | 0.76  (0.42, 1.39) | .376 | 243 | 0.97  (0.54, 1.73) | .924 | 243 |
| **Self-Isolating Given Suspected COVID-19 Infection** | | | | | | | | | | | | | | | |
| Unadjusted | 1.45  (1.00, 2.11) | .050 | 457 | 1.68  (1.12, 2.52) | .012 | 462 | 1.14  (0.78, 1.66) | .510 | 458 | 0.90  (0.61, 1.34) | .621 | 460 | 1.43  (0.98, 2.08) | .065 | 459 |
| Partially Adjusted | 1.62  (1.05, 2.49) | .029 | 358 | 1.94  (1.22, 3.07) | .005 | 361 | 1.24  (0.80, 1.91) | .332 | 358 | 0.89  (0.57, 1.41) | .624 | 359 | 1.64  (1.06, 2.53) | .025 | 360 |
| Fully Adjusted | 1.51  (0.86, 2.66) | .148 | 218 | 1.86  (1.00, 3.45) | .049 | 219 | 1.15  (0.65, 2.04) | .635 | 218 | 0.97  (0.53, 1.77) | .912 | 218 | 1.41  (0.80, 2.50) | .232 | 219 |
| **Face-To-Face Contact Outside Household** | | | | | | | | | | | | | | | |
| Unadjusted | 0.88  (0.75, 1.03) | .114 | 2546 | 0.97  (0.81, 1.16) | .734 | 2586 | 0.81  (0.70, 0.95) | .011 | 2551 | 0.89  (0.76, 1.05) | .164 | 2581 | 0.87  (0.74, 1.02) | .087 | 2553 |
| Partially Adjusted | 0.82  (0.68, 0.98) | .032 | 2006 | 0.94  (0.76, 1.16) | .565 | 2036 | 0.74  (0.61, 0.89) | .001 | 2009 | 0.83  (0.69, 1.00) | .047 | 2030 | 0.81  (0.68, 0.98) | .030 | 2013 |
| Fully Adjusted | 0.98  (0.77, 1.25) | .901 | 1242 | 0.89  (0.67, 1.17) | .389 | 1258 | 0.83  (0.66, 1.06) | .141 | 1244 | 0.86  (0.67, 1.10) | .240 | 1254 | 0.93  (0.73, 1.18) | .553 | 1246 |
| **Physical Contact Outside Household** | | | | | | | | | | | | | | | |
| Unadjusted | 0.80  (0.66, 0.97) | .026 | 2447 | 0.88  (0.70, 1.11) | .265 | 2487 | 0.82  (0.68, 1.00) | .048 | 2450 | 0.80  (0.65, 0.97) | .026 | 2481 | 0.82  (0.67, 1.01) | .058 | 2454 |
| Partially Adjusted | 0.81  (0.65, 1.01) | .066 | 1932 | 0.84  (0.64, 1.10) | .198 | 1962 | 0.85  (0.68, 1.07) | .172 | 1934 | 0.84  (0.66, 1.05) | .131 | 1955 | 0.87  (0.69, 1.09) | .231 | 1939 |
| Fully Adjusted | 0.77  (0.57, 1.04) | .092 | 1203 | 0.69  (0.48, 0.99) | .043 | 1219 | 0.83  (0.61, 1.12) | .222 | 1204 | 0.71  (0.51, 0.97) | .031 | 1214 | 0.87  (0.64, 1.18) | .368 | 1207 |

Note. Logistic regressions. OR = odds ratio. CI = confidence interval. Partially adjusted = adjusted for sociodemographic variables (age, gender, education, and keyworker status). Fully adjusted = additionally adjusted for prior mental health and risk behaviour variables (anxiety, depression, high-risk drinking, smoking, and suspected COVID-19 infection). All variables in the models are binary except age, which is continuous for the young person cohort. All risk perception variables were dichotomised at the median.

**Supplementary Table S10**

*Complete Case Cross-Sectional Associations between COVID-19 Risk Perceptions and Mental Health, Wellbeing, and Risk Behaviours (Whole Sample)*

|  | **COVID-19 Risk Perceptions (Exposures)** | | | | | | | | | | | | | | |
| --- | --- | --- | --- | --- | --- | --- | --- | --- | --- | --- | --- | --- | --- | --- | --- |
|  | **Holistic** | | | **Cognitive** | | | **Affective** | | | **Self** | | | **Other** | | |
| **Outcome and Model** | **OR**  **(95% CI)** | **P** | **N** | **OR**  **(95% CI)** | **P** | **N** | **OR**  **(95% CI)** | **P** | **N** | **OR**  **(95% CI)** | **P** | **N** | **OR**  **(95% CI)** | **P** | **N** |
| **Generalised Anxiety Disorder** | | | | | | | | | | | | | | | |
| Unadjusted | 2.76  (2.20, 3.46) | <.001 | 2780 | 1.68  (1.35, 2.09) | <.001 | 2820 | 2.65  (2.10, 3.33) | <.001 | 2787 | 2.19  (1.75, 2.74) | <.001 | 2806 | 3.01  (2.37, 3.81) | <.001 | 2796 |
| Partially Adjusted | 2.89  (2.29, 3.64) | <.001 | 2780 | 1.61  (1.28, 2.01) | <.001 | 2820 | 2.84  (2.24, 3.61) | <.001 | 2787 | 2.65  (2.10, 3.34) | <.001 | 2806 | 2.83  (2.23, 3.61) | <.001 | 2796 |
| Fully Adjusted | 2.78  (2.20, 3.52) | <.001 | 2780 | 1.50  (1.19, 1.89) | .001 | 2820 | 2.70  (2.12, 3.44) | <.001 | 2787 | 2.63  (2.08, 3.32) | <.001 | 2806 | 2.71  (2.12, 3.45) | <.001 | 2796 |
| **Depression** | | | | | | | | | | | | | | | |
| Unadjusted | 1.71  (1.32, 2.23) | <.001 | 2753 | 1.26  (0.97, 1.63) | .084 | 2793 | 1.82  (1.39, 2.38) | <.001 | 2760 | 1.37  (1.06, 1.79) | .018 | 2778 | 2.15  (1.63, 2.84) | <.001 | 2770 |
| Partially Adjusted | 1.75  (1.33, 2.30) | <.001 | 2753 | 1.22  (0.93, 1.60) | .145 | 2793 | 1.90  (1.43, 2.52) | <.001 | 2760 | 1.62  (1.23, 2.15) | .001 | 2778 | 2.02  (1.52, 2.67) | <.001 | 2770 |
| Fully Adjusted | 1.65  (1.24, 2.18) | <.001 | 2753 | 1.12  (0.85, 1.47) | .430 | 2793 | 1.77  (1.33, 2.36) | <.001 | 2760 | 1.57  (1.19, 2.09) | .002 | 2778 | 1.92  (1.44, 2.56) | <.001 | 2770 |
| **Low Wellbeing** | | | | | | | | | | | | | | | |
| Unadjusted | 1.75  (1.46, 2.11) | <.001 | 2766 | 1.26  (1.05, 1.51) | .014 | 2806 | 1.80  (1.49, 2.16) | <.001 | 2773 | 1.57  (1.31, 1.89) | <.001 | 2791 | 1.68  (1.40, 2.02) | <.001 | 2783 |
| Partially Adjusted | 1.83  (1.51, 2.21) | <.001 | 2766 | 1.24  (1.03, 1.49) | .025 | 2806 | 1.91  (1.58, 2.31) | <.001 | 2773 | 1.82  (1.50, 2.20) | <.001 | 2791 | 1.63  (1.35, 1.97) | <.001 | 2783 |
| Fully Adjusted | 1.76  (1.45, 2.13) | <.001 | 2766 | 1.18  (0.97, 1.43) | .090 | 2806 | 1.83  (1.50, 2.22) | <.001 | 2773 | 1.79  (1.48, 2.17) | <.001 | 2791 | 1.56  (1.29, 1.89) | <.001 | 2783 |
| **High-Risk Drinking** | | | | | | | | | | | | | | | |
| Unadjusted | 0.87  (0.74, 1.01) | .071 | 2809 | 1.13  (0.97, 1.32) | .107 | 2849 | 0.81  (0.70, 0.95) | .009 | 2816 | 0.85  (0.73, 0.99) | .034 | 2834 | 0.96  (0.82, 1.11) | .559 | 2826 |
| Partially Adjusted | 0.92  (0.78, 1.07) | .269 | 2809 | 1.13  (0.97, 1.32) | .120 | 2849 | 0.86  (0.74, 1.06) | .063 | 2816 | 0.89  (0.76, 1.05) | .167 | 2834 | 1.00  (0.85, 1.16) | .957 | 2826 |
| Fully Adjusted | 0.95  (0.79, 1.13) | .537 | 2809 | 1.18  (0.99, 1.40) | .059 | 2849 | 0.89  (0.74, 1.06) | .188 | 2816 | 0.98  (0.82, 1.18) | .860 | 2834 | 0.97  (0.82, 1.15) | .726 | 2826 |
| **Increased Alcohol Use** | | | | | | | | | | | | | | | |
| Unadjusted | 1.47  (1.25, 1.72) | <.001 | 2541 | 1.13  (0.97, 1.32) | .130 | 2578 | 1.52  (1.30, 1.78) | <.001 | 2544 | 1.37  (1.17, 1.60) | <.001 | 2561 | 1.33  (1.14, 1.55) | <.001 | 2556 |
| Partially Adjusted | 1.46  (1.24, 1.71) | <.001 | 2541 | 1.14  (0.97, 1.33) | .114 | 2578 | 1.52  (1.29, 1.78) | <.001 | 2544 | 1.37  (1.17, 1.62) | <.001 | 2561 | 1.31  (1.12, 1.53) | .001 | 2556 |
| Fully Adjusted | 1.46  (1.24, 1.72) | <.001 | 2541 | 1.13  (0.96, 1.32) | .140 | 2578 | 1.52  (1.29, 1.79) | <.001 | 2544 | 1.39  (1.18, 1.64) | <.001 | 2561 | 1.29  (1.10, 1.52) | .001 | 2556 |
| **Increased Smoking/E-Cigarette Use** | | | | | | | | | | | | | | | |
| Unadjusted | 1.26  (0.85, 1.89) | .253 | 420 | 1.12  (0.76, 1.66) | .567 | 426 | 1.14  (0.76, 1.69) | .527 | 420 | 1.00  (0.67, 1.49) | .990 | 422 | 1.57  (1.06, 2.34) | .025 | 423 |
| Partially Adjusted | 1.25  (0.81, 1.93) | .309 | 420 | 1.09  (0.72, 1.63) | .685 | 426 | 1.07  (0.70, 1.63) | .756 | 420 | 1.08  (0.71, 1.65) | .721 | 422 | 1.41  (0.93, 2.13) | .105 | 423 |
| Fully Adjusted | 1.14  (0.72, 1.80) | .586 | 420 | 0.97  (0.63, 1.49) | .888 | 426 | 0.98  (0.63, 1.54) | .942 | 420 | 1.00  (0.64, 1.57) | .989 | 422 | 1.31  (0.85, 2.03) | .222 | 423 |
| **Self-Isolating Given Suspected COVID-19 Infection** | | | | | | | | | | | | | | | |
| Unadjusted | 1.57  (1.04, 2.35) | .031 | 413 | 2.27  (1.50, 3.44) | <.001 | 422 | 1.17  (0.78, 1.75) | .451 | 415 | 0.89  (0.59, 1.34) | .564 | 416 | 1.43  (0.95, 2.13) | .085 | 421 |
| Partially Adjusted | 1.76  (1.15, 2.70) | .009 | 413 | 2.19  (1.44, 3.34) | <.001 | 422 | 1.35  (0.88, 2.07) | .164 | 415 | 1.03  (0.66, 1.60) | .899 | 416 | 1.46  (0.97, 2.20) | .069 | 421 |
| Fully Adjusted | 1.74  (1.13, 2.68) | .012 | 413 | 2.27  (1.48, 3.48) | <.001 | 422 | 1.30  (0.85, 2.01) | .231 | 415 | 0.98  (0.63, 1.54) | .943 | 416 | 1.42  (0.94, 2.16) | .096 | 421 |
| **Face-To-Face Contact Outside Household** | | | | | | | | | | | | | | | |
| Unadjusted | 0.94  (0.80, 1.09) | .405 | 2816 | 0.87  (0.74, 1.02) | .086 | 2857 | 0.90  (0.77, 1.06) | .211 | 2823 | 1.01  (0.86, 1.18) | .906 | 2842 | 0.92  (0.78, 1.08) | .291 | 2833 |
| Partially Adjusted | 0.82  (0.69, 0.96) | .016 | 2816 | 0.91  (0.77, 1.07) | .246 | 2857 | 0.77  (0.65, 0.91) | .002 | 2823 | 0.81  (0.69, 0.95) | .012 | 2842 | 0.88  (0.74, 1.03) | .118 | 2833 |
| Fully Adjusted | 0.83  (0.70, 0.98) | .027 | 2816 | 0.91  (0.77, 1.08) | .280 | 2857 | 0.78  (0.66, 0.92) | .004 | 2823 | 0.82  (0.69, 0.97) | .019 | 2842 | 0.88  (0.75, 1.05) | .149 | 2833 |
| **Physical Contact Outside Household** | | | | | | | | | | | | | | | |
| Unadjusted | 0.90  (0.75, 1.08) | .273 | 2662 | 0.79  (0.66, 0.95) | .012 | 2700 | 0.87  (0.73, 1.04) | .137 | 2668 | 0.81  (0.67, 0.97) | .020 | 2685 | 0.89  (0.75, 1.07) | .226 | 2678 |
| Partially Adjusted | 0.83  (0.69, 1.01) | .060 | 2662 | 0.79  (0.66, 0.95) | .012 | 2700 | 0.80  (0.66, 0.96) | .017 | 2668 | 0.72  (0.59, 0.87) | .001 | 2685 | 0.85  (0.71, 1.02) | .082 | 2678 |
| Fully Adjusted | 0.83  (0.68, 1.00) | .049 | 2662 | 0.78  (0.65, 0.94) | .008 | 2700 | 0.79  (0.65, 0.95) | .013 | 2668 | 0.71  (0.59, 0.86) | <.001 | 2685 | 0.84  (0.69, 1.01) | .057 | 2678 |

*Note.* Logistic regressions. OR = odds ratio. CI = confidence interval. Complete case = participants who had complete data on all covariates included in the fully adjusted regression models. Partially adjusted = adjusted for sociodemographic variables (age, gender, education, and keyworker status). Fully adjusted = additionally adjusted for prior mental health and risk behaviour variables (anxiety, depression, high-risk drinking, smoking, and suspected COVID-19 infection). All variables in the models are binary. All risk perception variables were dichotomised at the median.

**Supplementary Table S11**

*Complete Case Cross-Sectional Associations between COVID-19 Risk Perceptions and Mental Health, Wellbeing, and Risk Behaviours (Mothers)*

|  | **COVID-19 Risk Perceptions (Exposures)** | | | | | | | | | | | | | | |
| --- | --- | --- | --- | --- | --- | --- | --- | --- | --- | --- | --- | --- | --- | --- | --- |
|  | **Holistic** | | | **Cognitive** | | | **Affective** | | | **Self** | | | **Other** | | |
| **Outcome and Model** | **OR**  **(95% CI)** | **P** | **N** | **OR**  **(95% CI)** | **P** | **N** | **OR**  **(95% CI)** | **P** | **N** | **OR**  **(95% CI)** | **P** | **N** | **OR**  **(95% CI)** | **P** | **N** |
| **Generalised Anxiety Disorder** | | | | | | | | | | | | | | | |
| Unadjusted | 3.63  (2.44, 5.41) | <.001 | 1553 | 1.75  (1.23, 2.48) | .002 | 1578 | 3.91  (2.62, 5.84) | <.001 | 1558 | 3.71  (2.56, 5.39) | <.001 | 1567 | 3.37  (2.28, 5.00) | <.001 | 1566 |
| Partially Adjusted | 3.52  (2.36, 5.25) | <.001 | 1553 | 1.78  (1.25, 2.53) | .001 | 1578 | 3.79  (2.53, 5.68) | <.001 | 1558 | 3.62  (2.49, 5.27) | <.001 | 1567 | 3.27  (2.20, 4.86) | <.001 | 1566 |
| Fully Adjusted | 3.42  (2.28, 5.14) | <.001 | 1553 | 1.72  (1.21, 2.47) | .003 | 1578 | 3.68  (2.45, 5.55) | <.001 | 1558 | 3.54  (2.42, 5.19) | <.001 | 1567 | 3.08  (2.06, 4.60) | <.001 | 1566 |
| **Depression** | | | | | | | | | | | | | | | |
| Unadjusted | 2.04  (1.25, 3.33) | .004 | 1523 | 1.15  (0.72, 1.83) | .572 | 1547 | 2.25  (1.37, 3.69) | .001 | 1528 | 1.88  (1.17, 3.01) | .009 | 1536 | 1.77  (1.09, 2.87) | .020 | 1536 |
| Partially Adjusted | 1.86  (1.13, 3.04) | .014 | 1523 | 1.18  (0.73, 1.89) | .498 | 1547 | 2.03  (1.23, 3.33) | .005 | 1528 | 1.70  (1.05, 2.73) | .030 | 1536 | 1.64  (1.01, 2.67) | .046 | 1536 |
| Fully Adjusted | 1.75  (1.06, 2.89) | .029 | 1523 | 1.14  (0.70, 1.84) | .596 | 1547 | 1.91  (1.15, 3.17) | .012 | 1528 | 1.64  (1.01, 2.66) | .046 | 1536 | 1.52  (0.93, 2.49) | .098 | 1536 |
| **Low Wellbeing** | | | | | | | | | | | | | | | |
| Unadjusted | 2.24  (1.69, 2.97) | <.001 | 1532 | 1.30  (0.99, 1.70) | .056 | 1556 | 2.50  (1.88, 3.33) | <.001 | 1537 | 1.95  (1.48, 2.56) | <.001 | 1545 | 1.88  (1.42, 2.47) | <.001 | 1545 |
| Partially Adjusted | 2.14  (1.61, 2.84) | <.001 | 1532 | 1.33  (1.01, 1.74) | .041 | 1556 | 2.39  (1.79, 3.18) | <.001 | 1537 | 1.86  (1.41, 2.45) | <.001 | 1545 | 1.80  (1.36, 2.38) | <.001 | 1545 |
| Fully Adjusted | 2.06  (1.55, 2.76) | <.001 | 1532 | 1.28  (0.97, 1.69) | .076 | 1556 | 2.31  (1.73, 3.10) | <.001 | 1537 | 1.79  (1.35, 2.37) | <.001 | 1545 | 1.71  (1.28, 2.26) | <.001 | 1545 |
| **High-Risk Drinking** | | | | | | | | | | | | | | | |
| Unadjusted | 0.95  (0.78, 1.17) | .654 | 1563 | 1.13  (0.92, 1.39) | .247 | 1587 | 0.89  (0.73, 1.10) | .285 | 1568 | 0.98  (0.79, 1.21) | .843 | 1576 | 0.91  (0.74, 1.11) | .350 | 1576 |
| Partially Adjusted | 0.96  (0.78, 1.18) | .689 | 1563 | 1.13  (0.92, 1.39) | .233 | 1587 | 0.89  (0.73, 1.10) | .293 | 1568 | 0.98  (0.79, 1.21) | .828 | 1576 | 0.91  (0.74, 1.12) | .367 | 1576 |
| Fully Adjusted | 0.98  (0.77, 1.25) | .877 | 1563 | 1.17  (0.92, 1.48) | .208 | 1587 | 0.92  (0.72, 1.17) | .482 | 1568 | 0.91  (0.71, 1.17) | .473 | 1576 | 0.88  (0.69, 1.13) | .321 | 1576 |
| **Increased Alcohol Use** | | | | | | | | | | | | | | | |
| Unadjusted | 1.77  (1.43, 2.18) | <.001 | 1449 | 1.15  (0.93, 1.41) | .192 | 1471 | 1.63  (1.32, 2.01) | <.001 | 1452 | 1.51  (1.22, 1.87) | <.001 | 1460 | 1.53  (1.25, 1.89) | <.001 | 1460 |
| Partially Adjusted | 1.75  (1.42, 2.16) | <.001 | 1449 | 1.16  (0.94, 1.43) | .162 | 1471 | 1.61  (1.30, 1.99) | <.001 | 1452 | 1.49  (1.20, 1.84) | <.001 | 1460 | 1.52  (1.23, 1.87) | <.001 | 1460 |
| Fully Adjusted | 1.77  (1.43, 2.19) | <.001 | 1449 | 1.15  (0.93, 1.42) | .192 | 1471 | 1.63  (1.32, 2.02) | <.001 | 1452 | 1.48  (1.19, 1.83) | <.001 | 1460 | 1.52  (1.23, 1.88) | <.001 | 1460 |
| **Increased Smoking/E-Cigarette Use** | | | | | | | | | | | | | | | |
| Unadjusted | 2.07  (1.08, 3.96) | .028 | 177 | 1.13  (0.60, 2.11) | .704 | 182 | 2.07  (1.08, 3.96) | .028 | 177 | 1.60  (0.84, 3.02) | .151 | 179 | 1.66  (0.88, 3.11) | .116 | 180 |
| Partially Adjusted | 2.14  (1.08, 4.26) | .030 | 177 | 1.17  (0.61, 2.23) | .636 | 182 | 2.09  (1.05, 4.16) | .036 | 177 | 1.53  (0.79, 2.98) | .209 | 179 | 1.59  (0.83, 3.05) | .161 | 180 |
| Fully Adjusted | 1.88  (0.90, 3.90) | .092 | 177 | 1.09  (0.55, 2.15) | .806 | 182 | 1.98  (0.95, 4.14) | .068 | 177 | 1.37  (0.67, 2.79) | .391 | 179 | 1.41  (0.71, 2.82) | .328 | 180 |
| **Self-Isolating Given Suspected COVID-19 Infection** | | | | | | | | | | | | | | | |
| Unadjusted | 1.68  (0.89, 3.17) | .108 | 195 | 1.59  (0.86, 2.92) | .138 | 203 | 1.41  (0.76, 2.63) | .274 | 197 | 0.98  (0.52, 1.84) | .951 | 198 | 1.50  (0.81, 2.79) | .200 | 202 |
| Partially Adjusted | 1.76  (0.92, 3.38) | .089 | 195 | 1.68  (0.90, 3.14) | .103 | 203 | 1.52  (0.80, 2.91) | .202 | 197 | 1.08  (0.56, 2.05) | .825 | 198 | 1.65  (0.87, 3.14) | .125 | 202 |
| Fully Adjusted | 1.72  (0.88, 3.34) | .111 | 195 | 1.70  (0.90, 3.21) | .105 | 203 | 1.43  (0.74, 2.76) | .284 | 197 | 0.96  (0.49, 1.89) | .912 | 198 | 1.54  (0.80, 2.99) | .197 | 202 |
| **Face-To-Face Contact Outside Household** | | | | | | | | | | | | | | | |
| Unadjusted | 0.78  (0.62, 0.98) | .032 | 1574 | 0.93  (0.74, 1.17) | .521 | 1599 | 0.74  (0.59, 0.93) | .010 | 1579 | 0.81  (0.64, 1.02) | .079 | 1588 | 0.85  (0.68, 1.07) | .162 | 1587 |
| Partially Adjusted | 0.76  (0.60, 0.96) | .019 | 1574 | 0.93  (0.74, 1.17) | .515 | 1599 | 0.71  (0.57, 0.90) | .005 | 1579 | 0.80  (0.63, 1.01) | .062 | 1588 | 0.83  (0.66, 1.04) | .110 | 1587 |
| Fully Adjusted | 0.76  (0.60, 0.96) | .023 | 1574 | 0.93  (0.74, 1.17) | .531 | 1599 | 0.72  (0.57, 0.91) | .006 | 1579 | 0.80  (0.63, 1.01) | .066 | 1588 | 0.84  (0.66, 1.06) | .135 | 1587 |
| **Physical Contact Outside Household** | | | | | | | | | | | | | | | |
| Unadjusted | 0.85  (0.67, 1.08) | .175 | 1459 | 0.84  (0.66, 1.06) | .139 | 1481 | 0.81  (0.64, 1.02) | .074 | 1464 | 0.84  (0.66, 1.07) | .165 | 1471 | 0.85  (0.67, 1.08) | .186 | 1471 |
| Partially Adjusted | 0.83  (0.65, 1.06) | .131 | 1459 | 0.84  (0.66, 1.06) | .140 | 1481 | 0.79  (0.62, 1.00) | .051 | 1464 | 0.83  (0.65, 1.07) | .146 | 1471 | 0.83  (0.66, 1.06) | .132 | 1471 |
| Fully Adjusted | 0.82  (0.64, 1.04) | .100 | 1459 | 0.82  (0.66, 1.04) | .107 | 1481 | 0.77  (0.60, 0.98) | .033 | 1464 | 0.82  (0.64, 1.05) | .115 | 1471 | 0.81  (0.64, 1.03) | .088 | 1471 |

*Note.* Logistic regressions. OR = odds ratio. CI = confidence interval. Complete case = participants who had complete data on all covariates included in the fully adjusted regression models. Partially adjusted = adjusted for sociodemographic variables (age, education, and keyworker status). Fully adjusted = additionally adjusted for prior mental health and risk behaviour variables (anxiety, depression, high-risk drinking, smoking, and suspected COVID-19 infection). All variables in the models are binary. All risk perception variables were dichotomised at the median.

**Supplementary Table S12**

*Complete Case Cross-Sectional Associations between COVID-19 Risk Perceptions and Mental Health, Wellbeing, and Risk Behaviours (Young People)*

|  | **COVID-19 Risk Perceptions (Exposures)** | | | | | | | | | | | | | | |
| --- | --- | --- | --- | --- | --- | --- | --- | --- | --- | --- | --- | --- | --- | --- | --- |
|  | **Holistic** | | | **Cognitive** | | | **Affective** | | | **Self** | | | **Other** | | |
| **Outcome and Model** | **OR**  **(95% CI)** | **P** | **N** | **OR**  **(95% CI)** | **P** | **N** | **OR**  **(95% CI)** | **P** | **N** | **OR**  **(95% CI)** | **P** | **N** | **OR**  **(95% CI)** | **P** | **N** |
| **Generalised Anxiety Disorder** | | | | | | | | | | | | | | | |
| Unadjusted | 2.73  (2.04, 3.67) | <.001 | 1227 | 1.60  (1.17, 2.18) | .003 | 1242 | 2.77  (2.07, 3.70) | <.001 | 1229 | 2.53  (1.91, 3.37) | <.001 | 1239 | 2.51  (1.88, 3.34) | <.001 | 1230 |
| Partially Adjusted | 2.47  (1.83, 3.34) | <.001 | 1227 | 1.62  (1.18, 2.24) | .003 | 1242 | 2.50  (1.86, 3.36) | <.001 | 1229 | 2.35  (1.76, 3.15) | <.001 | 1239 | 2.33  (1.74, 3.11) | <.001 | 1230 |
| Fully Adjusted | 2.27  (1.66, 3.12) | <.001 | 1227 | 1.45  (1.03, 2.05) | .034 | 1242 | 2.37  (1.73, 3.24) | <.001 | 1229 | 2.21  (1.62, 3.01) | <.001 | 1239 | 2.28  (1.67, 3.10) | <.001 | 1230 |
| **Depression** | | | | | | | | | | | | | | | |
| Unadjusted | 2.19  (1.57, 3.05) | <.001 | 1230 | 1.26  (0.87, 1.81) | .220 | 1246 | 2.08  (1.50, 2.88) | <.001 | 1232 | 1.79  (1.30, 2.47) | <.001 | 1242 | 1.85  (1.34, 2.55) | <.001 | 1234 |
| Partially Adjusted | 1.96  (1.40, 2.75) | <.001 | 1230 | 1.31  (0.90, 1.90) | .154 | 1246 | 1.84  (1.32, 2.57) | <.001 | 1232 | 1.60  (1.15, 2.23) | .005 | 1242 | 1.72  (1.24, 2.39) | .001 | 1234 |
| Fully Adjusted | 1.79  (1.26, 2.55) | .001 | 1230 | 1.13  (0.76, 1.68) | .555 | 1246 | 1.74  (1.23, 2.47) | .002 | 1232 | 1.49  (1.05, 2.12) | .025 | 1242 | 1.65  (1.17, 2.34) | .004 | 1234 |
| **Low Wellbeing** | | | | | | | | | | | | | | | |
| Unadjusted | 1.78  (1.39, 2.29) | <.001 | 1234 | 1.18  (0.89, 1.57) | .257 | 1250 | 1.70  (1.32, 2.18) | <.001 | 1236 | 1.66  (1.29, 2.14) | <.001 | 1246 | 1.52  (1.18, 1.96) | .001 | 1238 |
| Partially Adjusted | 1.72  (1.33, 2.22) | <.001 | 1234 | 1.23  (0.92, 1.64) | .171 | 1250 | 1.62  (1.26, 2.10) | <.001 | 1236 | 1.59  (1.23, 2.05) | <.001 | 1246 | 1.49  (1.16, 1.93) | .002 | 1238 |
| Fully Adjusted | 1.60  (1.23, 2.08) | <.001 | 1234 | 1.15  (0.85, 1.55) | .369 | 1250 | 1.54  (1.18, 2.01) | .001 | 1236 | 1.48  (1.13, 1.93) | .004 | 1246 | 1.45  (1.11, 1.89) | .006 | 1238 |
| **High-Risk Drinking** | | | | | | | | | | | | | | | |
| Unadjusted | 0.87  (0.69, 1.09) | .224 | 1246 | 1.20  (0.91, 1.55) | .199 | 1262 | 0.72  (0.57, 0.91) | .006 | 1248 | 0.78  (0.61, 0.99) | .038 | 1258 | 0.89  (0.71, 1.13) | .343 | 1250 |
| Partially Adjusted | 0.96  (0.75, 1.21) | .711 | 1246 | 1.20  (0.92, 1.58) | .180 | 1262 | 0.79  (0.63, 1.01) | .058 | 1248 | 0.85  (0.67, 1.09) | .197 | 1258 | 0.95  (0.75, 1.21) | .678 | 1250 |
| Fully Adjusted | 1.01  (0.78, 1.30) | .969 | 1246 | 1.20  (0.89, 1.59) | .240 | 1262 | 0.82  (0.63, 1.06) | .132 | 1248 | 0.96  (0.74, 1.25) | .745 | 1258 | 0.94  (0.73, 1.22) | .642 | 1250 |
| **Increased Alcohol Use** | | | | | | | | | | | | | | | |
| Unadjusted | 1.20  (0.95, 1.53) | .128 | 1092 | 1.15  (0.87, 1.52) | .316 | 1107 | 1.28  (1.00, 1.62) | .048 | 1092 | 1.29  (1.00, 1.65) | .047 | 1101 | 0.99  (0.78, 1.27) | .957 | 1096 |
| Partially Adjusted | 1.18  (0.92, 1.50) | .200 | 1092 | 1.15  (0.87, 1.52) | .336 | 1107 | 1.25  (0.98, 1.60) | .077 | 1092 | 1.26  (0.98, 1.63) | .072 | 1101 | 0.97  (0.75, 1.24) | .783 | 1096 |
| Fully Adjusted | 1.18  (0.92, 1.51) | .194 | 1092 | 1.13  (0.85, 1.50) | .401 | 1107 | 1.27  (0.99, 1.64) | .060 | 1092 | 1.29  (1.00, 1.67) | .053 | 1101 | 0.96  (0.75, 1.24) | .750 | 1096 |
| **Increased Smoking/E-Cigarette Use** | | | | | | | | | | | | | | | |
| Unadjusted | 1.33  (0.80, 2.22) | .273 | 243 | 1.18  (0.62, 2.24) | .605 | 244 | 0.91  (0.54, 1.53) | .729 | 243 | 1.02  (0.60, 1.74) | .950 | 243 | 1.14  (0.68, 1.92) | .618 | 243 |
| Partially Adjusted | 1.11  (0.64, 1.92) | .706 | 243 | 1.13  (0.58, 2.19) | .715 | 244 | 0.72  (0.41, 1.26) | .250 | 243 | 0.85  (0.48, 1.49) | .571 | 243 | 0.99  (0.57, 1.72) | .982 | 243 |
| Fully Adjusted | 1.08  (0.61, 1.92) | .787 | 243 | 1.06  (0.53, 2.13) | .877 | 244 | 0.62  (0.34, 1.12) | .111 | 243 | 0.76  (0.42, 1.39) | .376 | 243 | 0.97  (0.54, 1.73) | .924 | 243 |
| **Self-Isolating Given Suspected COVID-19 Infection** | | | | | | | | | | | | | | | |
| Unadjusted | 1.58  (0.92, 2.71) | .097 | 218 | 1.79  (0.99, 3.24) | .054 | 219 | 1.28  (0.74, 2.23) | .374 | 218 | 1.10  (0.62, 1.96) | .739 | 218 | 1.51  (0.88, 2.60) | .137 | 219 |
| Partially Adjusted | 1.58  (0.91, 2.74) | .101 | 218 | 1.83  (1.00, 3.34) | .049 | 219 | 1.26  (0.72, 2.20) | .416 | 218 | 1.04  (0.58, 1.88) | .884 | 218 | 1.50  (0.86, 2.62) | .153 | 219 |
| Fully Adjusted | 1.51  (0.86, 2.66) | .148 | 218 | 1.86  (1.00, 3.45) | .049 | 219 | 1.15  (0.65, 2.04) | .635 | 218 | 0.97  (0.53, 1.77) | .912 | 218 | 1.41  (0.80, 2.50) | .232 | 219 |
| **Face-To-Face Contact Outside Household** | | | | | | | | | | | | | | | |
| Unadjusted | 1.06  (0.84, 1.33) | .630 | 1242 | 0.94  (0.72, 1.22) | .621 | 1258 | 0.91  (0.72, 1.14) | .412 | 1244 | 0.93  (0.74, 1.18) | .569 | 1254 | 0.99  (0.78, 1.25) | .923 | 1246 |
| Partially Adjusted | 0.96  (0.76, 1.22) | .733 | 1242 | 0.88  (0.67, 1.15) | .349 | 1258 | 0.81  (0.64, 1.03) | .088 | 1244 | 0.83  (0.65, 1.06) | .143 | 1254 | 0.91  (0.72, 1.16) | .469 | 1246 |
| Fully Adjusted | 0.98  (0.77, 1.25) | .901 | 1242 | 0.89  (0.67, 1.17) | .389 | 1258 | 0.83  (0.66, 1.06) | .141 | 1244 | 0.86  (0.67, 1.10) | .240 | 1254 | 0.93  (0.73, 1.18) | .553 | 1246 |
| **Physical Contact Outside Household** | | | | | | | | | | | | | | | |
| Unadjusted | 0.85  (0.64, 1.14) | .273 | 1203 | 0.77  (0.54, 1.10) | .151 | 1219 | 0.89  (0.67, 1.20) | .453 | 1204 | 0.75  (0.56, 1.02) | .070 | 1214 | 0.94  (0.70, 1.27) | .693 | 1207 |
| Partially Adjusted | 0.77  (0.57, 1.03) | .078 | 1203 | 0.69  (0.48, 0.99) | .045 | 1219 | 0.82  (0.60, 1.10) | .185 | 1204 | 0.69  (0.51, 0.95) | .022 | 1214 | 0.86  (0.63, 1.16) | .325 | 1207 |
| Fully Adjusted | 0.77  (0.57, 1.4) | .092 | 1203 | 0.69  (0.48, 0.99) | .043 | 1219 | 0.83  (0.61, 1.12) | .222 | 1204 | 0.71  (0.51, 0.97) | .031 | 1214 | 0.87  (0.64, 1.18) | .368 | 1207 |

*Note.* Logistic regressions. OR = odds ratio. CI = confidence interval. Complete case = participants who had complete data on all covariates included in the fully adjusted regression models. Partially adjusted = adjusted for sociodemographic variables (age, gender, education, and keyworker status). Fully adjusted = additionally adjusted for prior mental health and risk behaviour variables (anxiety, depression, high-risk drinking, smoking, and suspected COVID-19 infection). All variables in the models are binary except age, which is continuous for the young person cohort. All risk perception variables were dichotomised at the median.

**Supplementary Table S13**

*Prospective Longitudinal Associations between Pre-pandemic Mental Health, Wellbeing, and Risk Behaviours and COVID-19 Risk Perceptions (Young People)*

|  | **COVID-19 Risk Perceptions (Outcomes)** | | | | | | | | | | | | | | |
| --- | --- | --- | --- | --- | --- | --- | --- | --- | --- | --- | --- | --- | --- | --- | --- |
|  | **Holistic** | | | **Cognitive** | | | **Affective** | | | **Self** | | | **Other** | | |
| **Exposure and Model** | **OR**  **(95% CI)** | **P** | **N** | **OR**  **(95% CI)** | **P** | **N** | **OR**  **(95% CI)** | **P** | **N** | **OR**  **(95% CI)** | **P** | **N** | **OR**  **(95% CI)** | **P** | **N** |
| **Pre-pandemic Anxiety** | | | | | | | | | | | | | | | |
| Unadjusted | 1.83  (1.34, 2.49) | <.001 | 1911 | 1.50  (1.09, 2.08) | .014 | 1942 | 1.86  (1.37, 2.52) | <.001 | 1914 | 1.57  (1.16, 2.12) | .003 | 1935 | 1.88  (1.39, 2.55) | <.001 | 1918 |
| Partially Adjusted | 1.94  (1.36, 2.76) | <.001 | 1586 | 1.55  (1.08, 2.24) | .018 | 1611 | 1.99  (1.40, 2.82) | <.001 | 1588 | 1.61  (1.15, 2.27) | .006 | 1604 | 1.92  (1.37, 2.70) | <.001 | 1593 |
| Fully Adjusted | 1.89  (1.19, 3.00) | .007 | 1123 | 1.63  (1.01, 2.65) | .047 | 1138 | 1.82  (1.15, 2.87) | .010 | 1124 | 1.48  (0.94, 2.33) | .087 | 1134 | 2.02  (1.28, 3.17) | .002 | 1126 |
| **Pre-pandemic Depression** | | | | | | | | | | | | | | | |
| Unadjusted | 1.57  (1.18, 2.08) | .002 | 1917 | 1.28  (0.94, 1.75) | .121 | 1948 | 1.62  (1.22, 2.15) | .001 | 1920 | 1.50  (1.13, 1.99) | .005 | 1941 | 1.49  (1.13, 1.98) | .005 | 1924 |
| Partially Adjusted | 1.54  (1.10, 2.14) | .012 | 1591 | 1.41  (0.98, 2.02) | .065 | 1616 | 1.58  (1.14, 2.21) | .007 | 1593 | 1.60  (1.15, 2.22) | .005 | 1609 | 1.29  (0.93, 1.79) | .132 | 1598 |
| Fully Adjusted | 1.19  (0.75, 1.90) | .462 | 1123 | 1.09  (0.65, 1.84) | .737 | 1138 | 1.07  (0.68, 1.71) | .763 | 1124 | 1.40  (0.88, 2.21) | .156 | 1134 | 0.78  (0.49, 1.26) | .310 | 1126 |
| **Pre-pandemic Low Wellbeing** | | | | | | | | | | | | | | | |
| Unadjusted | 1.53  (1.22, 1.92) | <.001 | 1986 | 1.29  (1.00, 1.66) | .051 | 2019 | 1.52  (1.21, 1.90) | <.001 | 1991 | 1.48  (1.18, 1.86) | .001 | 2015 | 1.36  (1.08, 1.71) | .008 | 1991 |
| Partially Adjusted | 1.56  (1.20, 2.02) | .001 | 1682 | 1.22  (0.91, 1.64) | .178 | 1708 | 1.52  (1.17, 1.97) | .002 | 1685 | 1.41  (1.09, 1.83) | .008 | 1704 | 1.38  (1.07, 1.79) | .014 | 1687 |
| Fully Adjusted | 1.56  (1.10, 2.22) | .013 | 1065 | 0.92  (0.61, 1.39) | .684 | 1080 | 1.61  (1.13, 2.29) | .008 | 1066 | 1.48  (1.04, 2.11) | .029 | 1076 | 1.34  (0.94, 1.90) | .106 | 1068 |
| **Pre-pandemic High-Risk Drinking** | | | | | | | | | | | | | | | |
| Unadjusted | 0.75  (0.63, 0.90) | .002 | 1884 | 1.11  (0.90, 1.38) | .326 | 1912 | 0.70  (0.58, 0.83) | <.001 | 1888 | 0.65  (0.54, 0.78) | <.001 | 1908 | 0.84  (0.70, 1.02) | .075 | 1889 |
| Partially Adjusted | 0.89  (0.73, 1.09) | .262 | 1610 | 1.08  (0.85, 1.37) | .522 | 1632 | 0.84  (0.69, 1.03) | .098 | 1613 | 0.75  (0.61, 0.92) | .006 | 1628 | 0.94  (0.77, 1.16) | .560 | 1615 |
| Fully Adjusted | 0.92  (0.72, 1.19) | .535 | 1123 | 1.17  (0.87, 1.57) | .294 | 1138 | 0.89  (0.69, 1.15) | .366 | 1124 | 0.75  (0.58, 0.97) | .030 | 1134 | 1.04  (0.80, 1.34) | .779 | 1126 |
| **Pre-pandemic Smoking** | | | | | | | | | | | | | | | |
| Unadjusted | 1.06  (0.86, 1.30) | .580 | 2074 | 0.96  (0.76, 1.22) | .741 | 2101 | 1.15  (0.94, 1.42) | .169 | 2078 | 1.04  (0.84, 1.27) | .745 | 2097 | 1.09  (0.88, 1.34) | .431 | 2079 |
| Partially Adjusted | 0.99  (0.78, 1.25) | .921 | 1763 | 1.05  (0.80, 1.38) | .711 | 1785 | 1.08  (0.86, 1.37) | .501 | 1766 | 1.01  (0.80, 1.28) | .935 | 1781 | 1.08  (0.85, 1.37) | .517 | 1768 |
| Fully Adjusted | 0.79  (0.57, 1.10) | .162 | 1123 | 0.88  (0.60, 1.30) | .522 | 1138 | 0.83  (0.59, 1.15) | .266 | 1124 | 0.89  (0.63, 1.25) | .492 | 1134 | 0.87  (0.62, 1.22) | .417 | 1126 |
| **Pre-pandemic E-Cigarette Use** | | | | | | | | | | | | | | | |
| Unadjusted | 1.18  (0.77, 1.82) | .448 | 2074 | 0.88  (0.52, 1.48) | .627 | 2101 | 1.46  (0.95, 2.25) | .082 | 2078 | 1.03  (0.66, 1.59) | .910 | 2097 | 1.07  (0.69, 1.65) | .770 | 2079 |
| Partially Adjusted | 1.29  (0.77, 2.16) | .335 | 1763 | 0.89  (0.48, 1.66) | .714 | 1785 | 1.45  (0.86, 2.44) | .161 | 1766 | 0.91  (0.54, 1.53) | .713 | 1781 | 1.01  (0.61, 1.69) | .956 | 1768 |
| Fully Adjusted | 1.49  (0.72, 3.09) | .286 | 1123 | 0.90  (0.37, 2.17) | .810 | 1138 | 1.47  (0.71, 3.04) | .298 | 1124 | 0.70  (0.33, 1.49) | .351 | 1134 | 1.23  (0.61, 2.50) | .565 | 1126 |

Note. Logistic regressions. OR = odds ratio. CI = confidence interval. Partially adjusted = adjusted for sociodemographic variables (age, gender, education, and keyworker status). Fully adjusted = additionally adjusted for prior mental health and risk behaviour variables (pre-pandemic anxiety, depression, high-risk drinking, smoking, and early pandemic suspected COVID-19 infection). The same sociodemographic variables are included in all partially adjusted models. However, the variables in the fully adjusted models differ based on the exposure in each model (e.g., pre-pandemic anxiety is removed as a covariate when pre-pandemic anxiety is the exposure). All variables in the models are binary except age, which is continuous for the young person cohort. All risk perception variables were dichotomised at the median.

**Supplementary Table S14**

*Prospective Longitudinal Associations between Pre-pandemic Mental Health, Wellbeing, and Risk Behaviours and COVID-19 Risk Perceptions (Mothers)*

|  | **COVID-19 Risk Perceptions (Outcomes)** | | | | | | | | | | | | | | |
| --- | --- | --- | --- | --- | --- | --- | --- | --- | --- | --- | --- | --- | --- | --- | --- |
|  | **Holistic** | | | **Cognitive** | | | **Affective** | | | **Self** | | | **Other** | | |
| **Exposure and Model** | **OR**  **(95% CI)** | **P** | **N** | **OR**  **(95% CI)** | **P** | **N** | **OR**  **(95% CI)** | **P** | **N** | **OR**  **(95% CI)** | **P** | **N** | **OR**  **(95% CI)** | **P** | **N** |
| **Pre-pandemic Anxiety** | | | | | | | | | | | | | | | |
| Unadjusted | 1.26  (1.04, 1.52) | .017 | 2254 | 1.09  (0.90, 1.31) | .392 | 2293 | 1.32  (1.09, 1.60) | .004 | 2261 | 1.26  (1.04, 1.53) | .016 | 2272 | 1.37  (1.14, 1.66) | .001 | 2274 |
| Partially Adjusted | 1.30  (1.06, 1.59) | .010 | 2087 | 1.15  (0.94, 1.40) | .180 | 2122 | 1.35  (1.10, 1.64) | .004 | 2094 | 1.28  (1.05, 1.57) | .015 | 2105 | 1.43  (1.18, 1.75) | <.001 | 2104 |
| Fully Adjusted | 1.46  (1.10, 1.95) | .010 | 1410 | 1.29  (0.97, 1.72) | .076 | 1432 | 1.59  (1.19, 2.12) | .002 | 1415 | 1.56  (1.16, 2.09) | .003 | 1423 | 1.66  (1.25, 2.22) | .001 | 1421 |
| **Pre-pandemic Depression** | | | | | | | | | | | | | | | |
| Unadjusted | 1.09  (0.89, 1.33) | .413 | 2257 | 0.99  (0.80, 1.21) | .894 | 2295 | 1.06  (0.86, 1.29) | .529 | 2264 | 1.00  (0.81, 1.22) | .979 | 2275 | 1.15  (0.94, 1.41) | .171 | 2277 |
| Partially Adjusted | 1.06  (0.85, 1.31) | .605 | 2089 | 0.98  (0.79, 1.21) | .862 | 2124 | 1.04  (0.84, 1.28) | .729 | 2096 | 0.95  (0.77, 1.18) | .671 | 2107 | 1.16  (0.94, 1.43) | .169 | 2107 |
| Fully Adjusted | 0.92  (0.68, 1.26) | .610 | 1410 | 0.90  (0.67, 1.22) | .507 | 1432 | 0.87  (0.64, 1.18) | .363 | 1415 | 0.75  (0.55, 1.03) | .075 | 1423 | 0.91  (0.67, 1.24) | .545 | 1421 |
| **Pre-pandemic Low Wellbeing** | | | | | | | | | | | | | | | |
| Unadjusted | 1.43  (1.17, 1.74) | <.001 | 2070 | 1.29  (1.06, 1.57) | .012 | 2106 | 1.57  (1.28, 1.91) | <.001 | 2076 | 1.42  (1.17, 1.74) | <.001 | 2087 | 1.53  (1.25, 1.86) | <.001 | 2089 |
| Partially Adjusted | 1.41  (1.15, 1.74) | .001 | 1915 | 1.31  (1.06, 1.61) | .011 | 1949 | 1.52  (1.24, 1.88) | <.001 | 1921 | 1.35  (1.09, 1.66) | .005 | 1932 | 1.52  (1.23, 1.87) | <.001 | 1932 |
| Fully Adjusted | 1.31  (1.01, 1.70) | .038 | 1400 | 1.28  (0.99, 1.65) | .055 | 1422 | 1.39  (1.07, 1.80) | .013 | 1405 | 1.28  (0.98, 1.66) | .067 | 1413 | 1.32  (1.02, 1.71) | .033 | 1411 |
| **Pre-pandemic High-Risk Drinking** | | | | | | | | | | | | | | | |
| Unadjusted | 0.98  (0.80, 1.20) | .857 | 1854 | 1.02  (0.84, 1.24) | .849 | 1884 | 0.96  (0.78, 1.17) | .655 | 1860 | 1.10  (0.90, 1.35) | .343 | 1869 | 0.97  (0.79, 1.18) | .743 | 1871 |
| Partially Adjusted | 0.95  (0.77, 1.18) | .661 | 1722 | 1.00  (0.81, 1.23) | .985 | 1750 | 0.93  (0.76, 1.15) | .512 | 1728 | 1.12  (0.91, 1.38) | .295 | 1737 | 0.97  (0.79, 1.20) | .790 | 1737 |
| Fully Adjusted | 0.92  (0.73, 1.17) | .498 | 1410 | 1.02  (0.81, 1.29) | .869 | 1432 | 0.90  (0.71, 1.15) | .404 | 1415 | 1.09  (0.86, 1.39) | .477 | 1423 | 0.99  (0.78, 1.25) | .918 | 1421 |
| **Pre-pandemic Smoking** | | | | | | | | | | | | | | | |
| Unadjusted | 1.46  (1.04, 2.04) | .027 | 2061 | 1.31  (0.94, 1.82) | .107 | 2097 | 1.37  (0.98, 1.90) | .066 | 2067 | 1.70  (1.22, 2.37) | .002 | 2078 | 1.28  (0.92, 1.78) | .144 | 2080 |
| Partially Adjusted | 1.43  (1.00, 2.04) | .053 | 1907 | 1.29  (0.91, 1.84) | .151 | 1941 | 1.34  (0.94, 1.91) | .110 | 1913 | 1.72  (1.21, 2.46) | .003 | 1924 | 1.27  (0.89, 1.81) | .187 | 1924 |
| Fully Adjusted | 1.40  (0.91, 2.15) | .123 | 1410 | 1.21  (0.80, 1.84) | .373 | 1432 | 1.37  (0.89, 2.10) | .147 | 1415 | 1.91  (1.25, 2.93) | .003 | 1423 | 1.29  (0.85, 1.98) | .234 | 1421 |

Note. Logistic regressions. OR = odds ratio. CI = confidence interval. Partially adjusted = adjusted for sociodemographic variables (age, education, and keyworker status). Fully adjusted = additionally adjusted for prior mental health and risk behaviour variables (pre-pandemic anxiety, depression, high-risk drinking, smoking, and early pandemic suspected COVID-19 infection). The same sociodemographic variables are included in all partially adjusted models. However, the variables in the fully adjusted models differ based on the exposure in each model (e.g., pre-pandemic anxiety is removed as a covariate when pre-pandemic anxiety is the exposure). All variables in the models are binary. All risk perception variables were dichotomised at the median.

**Supplementary Table S15**

*Prospective Longitudinal Associations between Early Pandemic Risk Behaviours and COVID-19 Risk Perceptions (Mothers)*

|  | **COVID-19 Risk Perceptions (Outcomes)** | | | | | | | | | | | | | | |
| --- | --- | --- | --- | --- | --- | --- | --- | --- | --- | --- | --- | --- | --- | --- | --- |
|  | **Holistic** | | | **Cognitive** | | | **Affective** | | | **Self** | | | **Other** | | |
| **Exposure and Model** | **OR**  **(95% CI)** | **P** | **N** | **OR**  **(95% CI)** | **P** | **N** | **OR**  **(95% CI)** | **P** | **N** | **OR**  **(95% CI)** | **P** | **N** | **OR**  **(95% CI)** | **P** | **N** |
| **Early Pandemic Self-Isolating Given Suspected COVID-19 Infection** | | | | | | | | | | | | | | | |
| Unadjusted | 1.11  (0.65, 1.88) | .711 | 256 | 1.44  (0.86, 2.41) | .164 | 271 | 1.27  (0.75, 2.16) | .370 | 257 | 1.09  (0.65, 1.86) | .737 | 261 | 1.13  (0.67, 1.91) | .637 | 263 |
| Partially Adjusted | 1.12  (0.64, 1.98) | .694 | 239 | 1.44  (0.83, 2.49) | .196 | 253 | 1.35  (0.76, 2.40) | .308 | 240 | 1.26  (0.71, 2.23) | .438 | 244 | 1.12  (0.64, 1.95) | .688 | 246 |
| Fully Adjusted | 1.25  (0.63, 2.49) | .520 | 174 | 1.55  (0.81, 2.98) | .189 | 182 | 1.46  (0.73, 2.91) | .285 | 175 | 1.22  (0.60, 2.48) | .589 | 177 | 1.40  (0.71, 2.78) | .335 | 180 |
| **Early Pandemic Face-To-Face Contact Outside Household** | | | | | | | | | | | | | | | |
| Unadjusted | 0.94  (0.78, 1.13) | .491 | 2062 | 0.93  (0.78, 1.12) | .455 | 2104 | 0.97  (0.81, 1.17) | .755 | 2068 | 0.93  (0.78, 1.13) | .477 | 2080 | 0.96  (0.80, 1.15) | .639 | 2083 |
| Partially Adjusted | 0.92  (0.76, 1.12) | .389 | 1897 | 0.92  (0.76, 1.12) | .413 | 1935 | 0.95  (0.78, 1.15) | .596 | 1903 | 0.93  (0.76, 1.13) | .480 | 1915 | 0.95  (0.78, 1.15) | .574 | 1915 |
| Fully Adjusted | 0.96  (0.76, 1.22) | .740 | 1331 | 0.94  (0.75, 1.19) | .629 | 1353 | 0.96  (0.75, 1.21) | .709 | 1334 | 0.95  (0.74, 1.20) | .649 | 1342 | 1.07  (0.85, 1.36) | .552 | 1342 |
| **Early Pandemic Physical Contact Outside Household** | | | | | | | | | | | | | | | |
| Unadjusted | 0.84  (0.65, 1.09) | .192 | 1810 | 0.86  (0.67, 1.12) | .263 | 1844 | 0.91  (0.70, 1.18) | .464 | 1816 | 0.83  (0.64, 1.07) | .155 | 1824 | 0.90  (0.70, 1.17) | .440 | 1828 |
| Partially Adjusted | 0.85  (0.65, 1.11) | .239 | 1669 | 0.84  (0.64, 1.10) | .211 | 1699 | 0.91  (0.70, 1.20) | .515 | 1675 | 0.87  (0.66, 1.14) | .314 | 1683 | 0.91  (0.70, 1.19) | .498 | 1684 |
| Fully Adjusted | 0.91  (0.66, 1.25) | .551 | 1174 | 0.94  (0.68, 1.29) | .690 | 1194 | 0.88  (0.64, 1.22) | .440 | 1177 | 0.90  (0.65, 1.25) | .527 | 1184 | 1.04  (0.76, 1.44) | .790 | 1184 |

Note. Logistic regressions. OR = odds ratio. CI = confidence interval. Partially adjusted = adjusted for sociodemographic variables (age, education, and keyworker status). Fully adjusted = additionally adjusted for prior mental health and risk behaviour variables (pre-pandemic anxiety, depression, high-risk drinking, smoking, and early pandemic suspected COVID-19 infection). All variables in the models are binary. All risk perception variables were dichotomised at the median.

**Supplementary Table S16**

*Prospective Longitudinal Associations between Early Pandemic Risk Behaviours and COVID-19 Risk Perceptions (Young People)*

|  | **COVID-19 Risk Perceptions (Outcomes)** | | | | | | | | | | | | | | |
| --- | --- | --- | --- | --- | --- | --- | --- | --- | --- | --- | --- | --- | --- | --- | --- |
|  | **Holistic** | | | **Cognitive** | | | **Affective** | | | **Self** | | | **Other** | | |
| **Exposure and Model** | **OR**  **(95% CI)** | **P** | **N** | **OR**  **(95% CI)** | **P** | **N** | **OR**  **(95% CI)** | **P** | **N** | **OR**  **(95% CI)** | **P** | **N** | **OR**  **(95% CI)** | **P** | **N** |
| **Early Pandemic Face-To-Face Contact Outside Household** | | | | | | | | | | | | | | | |
| Unadjusted | 0.89  (0.74, 1.06) | .179 | 1994 | 0.87  (0.71, 1.07) | .178 | 2019 | 0.90  (0.76, 1.08) | .261 | 1999 | 0.82  (0.69, 0.99) | .035 | 2017 | 0.84  (0.70, 1.01) | .060 | 1998 |
| Partially Adjusted | 0.86  (0.70, 1.05) | .137 | 1648 | 0.81  (0.64, 1.03) | .087 | 1669 | 0.91  (0.74, 1.11) | .341 | 1650 | 0.82  (0.66, 1.00) | .055 | 1666 | 0.85  (0.70, 1.05) | .126 | 1652 |
| Fully Adjusted | 0.93  (0.72, 1.20) | .574 | 1080 | 0.89  (0.67, 1.19) | .446 | 1093 | 0.97  (0.75, 1.24) | .788 | 1081 | 0.85  (0.65, 1.10) | .208 | 1090 | 1.00  (0.78, 1.29) | .995 | 1083 |
| **Early Pandemic Physical Contact Outside Household** | | | | | | | | | | | | | | | |
| Unadjusted | 0.93  (0.73, 1.19) | .567 | 1809 | 0.86  (0.64, 1.15) | .303 | 1830 | 1.00  (0.78, 1.28) | .992 | 1812 | 0.97  (0.76, 1.25) | .830 | 1829 | 0.80  (0.62, 1.04) | .093 | 1813 |
| Partially Adjusted | 0.95  (0.72, 1.25) | .692 | 1497 | 0.82  (0.59, 1.14) | .242 | 1514 | 1.07  (0.81, 1.42) | .636 | 1499 | 1.00  (0.76, 1.33) | .977 | 1512 | 0.85  (0.64, 1.14) | .278 | 1501 |
| Fully Adjusted | 0.95  (0.66, 1.36) | .768 | 985 | 1.03  (0.69, 1.55) | .874 | 995 | 1.02  (0.71, 1.47) | .920 | 986 | 1.00  (0.69, 1.44) | .985 | 993 | 0.97  (0.67, 1.40) | .859 | 988 |

*Note*. Logistic regressions. OR = odds ratio. CI = confidence interval. Partially adjusted = adjusted for sociodemographic variables (age, gender, education, and keyworker status). Fully adjusted = additionally adjusted for prior mental health and risk behaviour variables (pre-pandemic anxiety, depression, high-risk drinking, smoking, and early pandemic suspected COVID-19 infection). All variables in the models are binary except age, which is continuous for the young person cohort. All risk perception variables were dichotomised at the median. Sample sizes were too small for statistical models on early pandemic self-isolating given suspected COVID-19 infection, so these results were removed.

**Supplementary Table S17**

*Complete Case Prospective Longitudinal Associations between Pre-pandemic Mental Health, Wellbeing, and Risk Behaviours and COVID-19 Risk Perceptions (Whole Sample)*

|  | **COVID-19 Risk Perceptions (Outcomes)** | | | | | | | | | | | | | | |
| --- | --- | --- | --- | --- | --- | --- | --- | --- | --- | --- | --- | --- | --- | --- | --- |
|  | **Holistic** | | | **Cognitive** | | | **Affective** | | | **Self** | | | **Other** | | |
| **Exposure and Model** | **OR**  **(95% CI)** | **P** | **N** | **OR**  **(95% CI)** | **P** | **N** | **OR**  **(95% CI)** | **P** | **N** | **OR**  **(95% CI)** | **P** | **N** | **OR**  **(95% CI)** | **P** | **N** |
| **Pre-pandemic Anxiety** | | | | | | | | | | | | | | | |
| Unadjusted | 1.71  (1.39, 2.10) | <.001 | 2533 | 1.25  (1.01, 1.53) | .036 | 2570 | 1.85  (1.50, 2.28) | <.001 | 2539 | 1.60  (1.30, 1.97) | <.001 | 2557 | 1.65  (1.34, 2.03) | <.001 | 2547 |
| Partially Adjusted | 1.59  (1.28, 1.97) | <.001 | 2533 | 1.34  (1.09, 1.66) | .006 | 2570 | 1.69  (1.36, 2.10) | <.001 | 2539 | 1.34  (1.08, 1.67) | .008 | 2557 | 1.67  (1.35, 2.07) | <.001 | 2547 |
| Fully Adjusted | 1.64  (1.29, 2.09) | <.001 | 2533 | 1.34  (1.06, 1.71) | .016 | 2570 | 1.74  (1.37, 2.22) | <.001 | 2539 | 1.44  (1.13, 1.84) | .003 | 2557 | 1.75  (1.37, 2.22) | <.001 | 2547 |
| **Pre-pandemic Depression** | | | | | | | | | | | | | | | |
| Unadjusted | 1.32  (1.06, 1.65) | .013 | 2533 | 1.07  (0.86, 1.33) | .543 | 2570 | 1.40  (1.12, 1.74) | .003 | 2539 | 1.25  (1.01, 1.56) | .043 | 2557 | 1.19  (0.95, 1.48) | .122 | 2547 |
| Partially Adjusted | 1.21  (0.96, 1.52) | .108 | 2533 | 1.14  (0.92, 1.42) | .230 | 2570 | 1.25  (1.00, 1.57) | .054 | 2539 | 1.05  (0.83, 1.32) | .706 | 2557 | 1.17  (0.94, 1.47) | .162 | 2547 |
| Fully Adjusted | 0.94  (0.73, 1.22) | .648 | 2533 | 0.98  (0.77, 1.26) | .903 | 2570 | 0.95  (0.74, 1.23) | .716 | 2539 | 0.86  (0.66, 1.12) | .263 | 2557 | 0.88  (0.68, 1.14) | .341 | 2547 |
| **Pre-pandemic Low Wellbeing** | | | | | | | | | | | | | | | |
| Unadjusted | 1.60  (1.31, 1.94) | <.001 | 2465 | 1.20  (0.99, 1.45) | .068 | 2502 | 1.74  (1.43, 2.12) | <.001 | 2471 | 1.69  (1.39, 2.06) | <.001 | 2489 | 1.46  (1.20, 1.78) | <.001 | 2479 |
| Partially Adjusted | 1.50  (1.23, 1.83) | <.001 | 2465 | 1.24  (1.02, 1.51) | .031 | 2502 | 1.62  (1.32, 1.98) | <.001 | 2471 | 1.55  (1.26, 1.89) | <.001 | 2489 | 1.43  (1.17, 1.74) | <.001 | 2479 |
| Fully Adjusted | 1.41  (1.15, 1.74) | .001 | 2465 | 1.19  (0.97, 1.46) | .101 | 2502 | 1.52  (1.24, 1.87) | <.001 | 2471 | 1.53  (1.24, 1.89) | <.001 | 2489 | 1.34  (1.09, 1.65) | .005 | 2479 |
| **Pre-pandemic High-Risk Drinking** | | | | | | | | | | | | | | | |
| Unadjusted | 0.78  (0.66, 0.92) | .002 | 2533 | 1.06  (0.91, 1.24) | .446 | 2570 | 0.75  (0.64, 0.88) | <.001 | 2539 | 0.62  (0.53, 0.73) | <.001 | 2557 | 1.00  (0.86, 1.18) | .962 | 2547 |
| Partially Adjusted | 0.89  (0.75, 1.05) | .167 | 2533 | 0.97  (0.82, 1.14) | .699 | 2570 | 0.88  (0.74, 1.04) | .137 | 2539 | 0.79  (0.66, 0.93) | .005 | 2557 | 1.03  (0.88, 1.22) | .695 | 2547 |
| Fully Adjusted | 0.89  (0.75, 1.06) | .192 | 2533 | 0.97  (0.82, 1.14) | .702 | 2570 | 0.89  (0.74, 1.05) | .169 | 2539 | 0.78  (0.65, 0.92) | .004 | 2557 | 1.02  (0.86, 1.21) | .807 | 2547 |
| **Pre-pandemic Smoking** | | | | | | | | | | | | | | | |
| Unadjusted | 0.94  (0.74, 1.21) | .648 | 2533 | 1.09  (0.86, 1.39) | .469 | 2570 | 0.92  (0.72, 1.17) | .509 | 2539 | 0.88  (0.69, 1.12) | .302 | 2557 | 1.24  (0.97, 1.58) | .083 | 2547 |
| Partially Adjusted | 0.97  (0.75, 1.25) | .817 | 2533 | 1.02  (0.80, 1.31) | .862 | 2570 | 0.96  (0.75, 1.24) | .754 | 2539 | 1.04  (0.81, 1.33) | .760 | 2557 | 1.17  (0.91, 1.50) | .226 | 2547 |
| Fully Adjusted | 0.96  (0.74, 1.24) | .755 | 2533 | 1.01  (0.78, 1.29) | .966 | 2570 | 0.94  (0.73, 1.22) | .667 | 2539 | 1.08  (0.84, 1.40) | .529 | 2557 | 1.12  (0.86, 1.45) | .394 | 2547 |

*Note.* Logistic regressions. OR = odds ratio. CI = confidence interval. Complete case = participants who had complete data on all covariates included in the fully adjusted regression models. Partially adjusted = adjusted for sociodemographic variables (age, gender, education, and keyworker status). Fully adjusted = additionally adjusted for prior mental health and risk behaviour variables (pre-pandemic anxiety, depression, high-risk drinking, smoking, and early pandemic suspected COVID-19 infection). The same sociodemographic variables are included in all partially adjusted models. However, the variables in the fully adjusted models differ based on the exposure in each model (e.g., pre-pandemic anxiety is removed as a covariate when pre-pandemic anxiety is the exposure). All variables in the models are binary. All risk perception variables were dichotomised at the median.

**Supplementary Table S18**

*Complete Case Prospective Longitudinal Associations between Pre-pandemic Mental Health, Wellbeing, and Risk Behaviours and COVID-19 Risk Perceptions (Mothers)*

|  | **COVID-19 Risk Perceptions (Outcomes)** | | | | | | | | | | | | | | |
| --- | --- | --- | --- | --- | --- | --- | --- | --- | --- | --- | --- | --- | --- | --- | --- |
|  | **Holistic** | | | **Cognitive** | | | **Affective** | | | **Self** | | | **Other** | | |
| **Exposure and Model** | **OR**  **(95% CI)** | **P** | **N** | **OR**  **(95% CI)** | **P** | **N** | **OR**  **(95% CI)** | **P** | **N** | **OR**  **(9% CI)** | **P** | **N** | **OR**  **(9% CI)** | **P** | **N** |
| **Pre-pandemic Anxiety** | | | | | | | | | | | | | | | |
| Unadjusted | 1.42  (1.10, 1.81) | .006 | 1410 | 1.24  (0.97, 1.58) | .090 | 1432 | 1.50  (1.17, 1.92) | .001 | 1415 | 1.40  (1.09, 1.80) | .008 | 1423 | 1.61  (1.25, 2.06) | <.001 | 1421 |
| Partially Adjusted | 1.43  (1.11, 1.84) | .005 | 1410 | 1.24  (0.97, 1.59) | .086 | 1432 | 1.52  (1.18, 1.95) | .001 | 1415 | 1.41  (1.09, 1.81) | .008 | 1423 | 1.63  (1.27, 2.09) | <.001 | 1421 |
| Fully Adjusted | 1.46  (1.10, 1.95) | .010 | 1410 | 1.29  (0.97, 1.72) | .076 | 1432 | 1.60  (1.19, 2.12) | .002 | 1415 | 1.56  (1.16, 2.09) | .003 | 1423 | 1.66  (1.25, 2.22) | .001 | 1421 |
| **Pre-pandemic Depression** | | | | | | | | | | | | | | | |
| Unadjusted | 1.15  (0.88, 1.50) | .295 | 1410 | 1.04  (0.80, 1.35) | .790 | 1432 | 1.13  (0.87, 1.47) | .353 | 1415 | 1.00  (0.76, 1.30) | .972 | 1423 | 1.21  (0.93, 1.57) | .157 | 1421 |
| Partially Adjusted | 1.15  (0.88, 1.50) | .300 | 1410 | 1.04  (0.80, 1.36) | .763 | 1432 | 1.13  (0.87, 1.48) | .354 | 1415 | 0.99  (0.75, 1.30) | .924 | 1423 | 1.21  (0.93, 1.58) | .150 | 1421 |
| Fully Adjusted | 0.92  (0.68, 1.26) | .610 | 1410 | 0.90  (0.67, 1.22) | .507 | 1432 | 0.87  (0.64, 1.18) | .363 | 1415 | 0.75  (0.55, 1.03) | .075 | 1423 | 0.91  (0.67, 1.24) | .545 | 1421 |
| **Pre-pandemic Low Wellbeing** | | | | | | | | | | | | | | | |
| Unadjusted | 1.38  (1.08, 1.76) | .011 | 1400 | 1.31  (1.02, 1.67) | .033 | 1422 | 1.44  (1.13, 1.84) | .004 | 1405 | 1.32  (1.03, 1.69) | .029 | 1413 | 1.42  (1.11, 1.82) | .005 | 1411 |
| Partially Adjusted | 1.37  (1.07, 1.76) | .012 | 1400 | 1.31  (1.02, 1.67) | .032 | 1422 | 1.45  (1.13, 1.86) | .004 | 1405 | 1.32  (1.02, 1.69) | .032 | 1413 | 1.42  (1.11, 1.82) | .006 | 1411 |
| Fully Adjusted | 1.31  (1.01, 1.70) | .038 | 1400 | 1.28  (0.99, 1.65) | .055 | 1422 | 1.39  (1.07, 1.80) | .013 | 1405 | 1.28  (0.98, 1.66) | .067 | 1413 | 1.32  (1.02, 1.71) | .033 | 1411 |
| **Pre-pandemic High-Risk Drinking** | | | | | | | | | | | | | | | |
| Unadjusted | 0.95  (0.75, 1.19) | .652 | 1410 | 1.03  (0.82, 1.30) | .793 | 1432 | 0.93  (0.74, 1.17) | .525 | 1415 | 1.15  (0.91, 1.45) | .235 | 1423 | 1.00  (0.80, 1.26) | .966 | 1421 |
| Partially Adjusted | 0.94  (0.75, 1.19) | .631 | 1410 | 1.04  (0.82, 1.31) | .750 | 1432 | 0.92  (0.73, 1.17) | .512 | 1415 | 1.14  (0.90, 1.45) | .270 | 1423 | 1.00  (0.80, 1.26) | .979 | 1421 |
| Fully Adjusted | 0.92  (0.73, 1.17) | .498 | 1410 | 1.02  (0.81, 1.29) | .869 | 1432 | 0.90  (0.71, 1.15) | .404 | 1415 | 1.09  (0.86, 1.39) | .477 | 1423 | 0.99  (0.78, 1.25) | .918 | 1421 |
| **Pre-pandemic Smoking** | | | | | | | | | | | | | | | |
| Unadjusted | 1.55  (1.02, 2.35) | .041 | 1410 | 1.22  (0.81, 1.84) | .347 | 1432 | 1.50  (0.99, 2.28) | .054 | 1415 | 2.07  (1.36, 3.13) | .001 | 1423 | 1.46  (0.97, 2.21) | .072 | 1421 |
| Partially Adjusted | 1.42  (0.93, 2.18) | .101 | 1410 | 1.23  (0.81, 1.86) | .323 | 1432 | 1.39  (0.91, 2.12) | .124 | 1415 | 1.95  (1.28, 2.97) | .002 | 1423 | 1.35  (0.89, 2.05) | .164 | 1421 |
| Fully Adjusted | 1.40  (0.91, 2.15) | .123 | 1410 | 1.21  (0.80, 1.84) | .374 | 1432 | 1.37  (0.89, 2.10) | .147 | 1415 | 1.91  (1.25, 2.93) | .003 | 1423 | 1.29  (0.85, 1.98) | .234 | 1421 |

*Note.* Logistic regressions. OR = odds ratio. CI = confidence interval. Complete case = participants who had complete data on all covariates included in the fully adjusted regression models. Partially adjusted = adjusted for sociodemographic variables (age, education, and keyworker status). Fully adjusted = additionally adjusted for prior mental health and risk behaviour variables (pre-pandemic anxiety, depression, high-risk drinking, smoking, and early pandemic suspected COVID-19 infection). The same sociodemographic variables are included in all partially adjusted models. However, the variables in the fully adjusted models differ based on the exposure in each model (e.g., pre-pandemic anxiety is removed as a covariate when pre-pandemic anxiety is the exposure). All variables in the models are binary. All risk perception variables were dichotomised at the median.

**Supplementary Table S19**

*Complete Case Prospective Longitudinal Associations Between Pre-pandemic Mental Health, Wellbeing, and Risk Behaviours and COVID-19 Risk Perceptions (Young People)*

|  | **COVID-19 Risk Perceptions (Outcomes)** | | | | | | | | | | | | | | |
| --- | --- | --- | --- | --- | --- | --- | --- | --- | --- | --- | --- | --- | --- | --- | --- |
|  | **Holistic** | | | **Cognitive** | | | **Affective** | | | **Self** | | | **Other** | | |
| **Exposure and Model** | **OR**  **(95% CI)** | **P** | **N** | **OR**  **(95% CI)** | **P** | **N** | **OR**  **(95% CI)** | **P** | **N** | **OR**  **(95% CI)** | **P** | **N** | **OR**  **(95% CI)** | **P** | **N** |
| **Pre-pandemic Anxiety** | | | | | | | | | | | | | | | |
| Unadjusted | 2.15  (1.43, 3.22) | <.001 | 1123 | 1.75  (1.15, 2.66) | .009 | 1138 | 1.97  (1.32, 2.93) | .001 | 1124 | 1.72  (1.16, 2.54) | .007 | 1134 | 1.94  (1.31, 2.88) | .001 | 1126 |
| Partially Adjusted | 2.01  (1.33, 3.05) | .001 | 1223 | 1.66  (1.08, 2.54) | .020 | 1138 | 1.85  (1.23, 2.79) | .003 | 1124 | 1.64  (1.10, 2.45) | .015 | 1134 | 1.80  (1.21, 2.69) | .004 | 1126 |
| Fully Adjusted | 1.89  (1.19, 3.00) | .007 | 1223 | 1.63  (1.01, 2.65) | .049 | 1138 | 1.82  (1.15, 2.87) | .010 | 1124 | 1.48  (0.94, 2.33) | .087 | 1134 | 2.02  (1.28, 3.17) | .002 | 1126 |
| **Pre-pandemic Depression** | | | | | | | | | | | | | | | |
| Unadjusted | 1.73  (1.15, 2.60) | .008 | 1123 | 1.32  (0.84, 2.08) | .223 | 1138 | 1.58  (1.05, 2.36) | .027 | 1124 | 1.81  (1.21, 2.71) | .004 | 1134 | 0.78  (0.49, 1.26) | .310 | 1126 |
| Partially Adjusted | 1.57  (1.04, 2.38) | .034 | 1123 | 1.32  (0.83, 2.09) | .237 | 1138 | 1.42  (0.94, 2.14) | .097 | 1124 | 1.68  (1.12, 2.54) | .013 | 1134 | 1.07  (0.71, 1.62) | .737 | 1126 |
| Fully Adjusted | 1.19  (0.75, 1.90) | .462 | 1123 | 1.09  (0.65, 1.84) | .737 | 1138 | 1.07  (0.68, 1.71) | .763 | 1124 | 1.40  (0.88, 2.21) | .156 | 1134 | 0.78  (0.49, 1.26) | .310 | 1126 |
| **Pre-pandemic Low Wellbeing** | | | | | | | | | | | | | | | |
| Unadjusted | 1.92  (1.39, 2.67) | <.001 | 1065 | 1.04  (0.72, 1.53) | .820 | 1080 | 1.93  (1.39, 2.67) | <.001 | 1066 | 1.81  (1.31, 2.51) | <.001 | 1076 | 1.51  (1.09, 2.08) | .013 | 1068 |
| Partially Adjusted | 1.81  (1.30, 2.53) | <.001 | 1065 | 1.03  (0.70, 1.52) | .876 | 1080 | 1.80  (1.29, 2.51) | .001 | 1066 | 1.68  (1.20, 2.34) | .002 | 1076 | 1.45  (1.04, 2.01) | .029 | 1068 |
| Fully Adjusted | 1.56  (1.10, 2.22) | .013 | 1065 | 0.92  (0.61, 1.39) | .684 | 1080 | 1.61  (1.13, 2.29) | .008 | 1066 | 1.48  (1.04, 2.11) | .029 | 1076 | 1.34  (0.94, 1.90) | .106 | 1068 |
| **Pre-pandemic High-Risk Drinking** | | | | | | | | | | | | | | | |
| Unadjusted | 0.78  (0.62, 0.99) | .040 | 1123 | 1.16  (0.87, 1.53) | .310 | 1138 | 0.75  (0.59, .095) | .017 | 1124 | 0.65  (0.51, 0.83) | .001 | 1134 | 0.94  (0.73, 1.19) | .591 | 1126 |
| Partially Adjusted | 0.87  (0.68, 1.10) | .244 | 1123 | 1.14  (0.86, 1.51) | .374 | 1138 | 0.84  (0.65, 1.07) | .158 | 1124 | 0.72  (0.56, 0.92) | .009 | 1134 | 1.00  (0.78, 1.28) | .993 | 1126 |
| Fully Adjusted | 0.92  (0.72, 1.19) | .535 | 1123 | 1.17  (0.87, 1.57) | .294 | 1138 | 0.89  (0.69, 1.15) | .366 | 1124 | 0.75  (0.58, 0.97) | .030 | 1134 | 1.04  (0.80, 1.34) | .779 | 1126 |
| **Pre-pandemic Smoking** | | | | | | | | | | | | | | | |
| Unadjusted | 0.89  (0.66, 1.21) | .464 | 1123 | 0.92  (0.64, 1.32) | .645 | 1138 | 0.92  (0.68, 1.25) | .608 | 1124 | 0.88  (0.64, 1.22) | .446 | 1134 | 0.95  (0.70, 1.30) | .757 | 1126 |
| Partially Adjusted | 0.84  (0.62, 1.15) | .277 | 1123 | 0.95  (0.66, 1.38) | .799 | 1138 | 0.86  (0.63, 1.18) | .352 | 1124 | 0.83  (0.60, 1.15) | .269 | 1134 | 0.93  (0.68, 1.27) | .648 | 1126 |
| Fully Adjusted | 0.79  (0.57, 1.10) | .162 | 1123 | 0.88  (0.60, 1.30) | .522 | 1138 | 0.83  (0.59, 1.15) | .266 | 1124 | 0.89  (0.63, 1.25) | .492 | 1134 | 0.87  (0.62, 1.22) | .417 | 1126 |
| **Pre-pandemic E-Cigarette Use** | | | | | | | | | | | | | | | |
| Unadjusted | 1.81  (0.91, 3.60) | .090 | 1123 | 0.86  (0.36, 1.95) | .684 | 1138 | 1.86  (0.94, 3.68) | .073 | 1124 | 0.94  (0.46, 1.91) | .866 | 1134 | 1.41  (0.72, 2.77) | .320 | 1126 |
| Partially Adjusted | 1.52  (0.75, 3.07) | .247 | 1123 | 0.96  (0.41, 2.27) | .930 | 1138 | 1.50  (0.75, 3.04) | .254 | 1124 | 0.74  (0.36, 1.54) | .423 | 1134 | 1.31  (0.66, 2.60) | .443 | 1126 |
| Fully Adjusted | 1.49  (0.72, 3.09) | .286 | 1123 | 0.90  (0.37, 2.17) | .810 | 1138 | 1.47  (0.71, 3.04) | .298 | 1124 | 0.70  (0.33, 1.49) | .351 | 1134 | 1.23  (0.61, 2.50) | .565 | 1126 |

*Note.* Logistic regressions. OR = odds ratio. CI = confidence interval. Complete case = participants who had complete data on all covariates included in the fully adjusted regression models. Partially adjusted = adjusted for sociodemographic variables (age, gender, education, and keyworker status). Fully adjusted = additionally adjusted for prior mental health and risk behaviour variables (pre-pandemic anxiety, depression, high-risk drinking, smoking, and early pandemic suspected COVID-19 infection). The same sociodemographic variables are included in all partially adjusted models. However, the variables in the fully adjusted models differ based on the exposure in each model (e.g., pre-pandemic anxiety is removed as a covariate when pre-pandemic anxiety is the exposure). All variables in the models are binary except age, which is continuous for the young person cohort. All risk perception variables were dichotomised at the median.

**Supplementary Table S20**

*Complete Case Prospective Longitudinal Associations between Early Pandemic Risk Behaviours and COVID-19 Risk Perceptions (Whole Sample)*

|  | **COVID-19 Risk Perceptions (Outcomes)** | | | | | | | | | | | | | | | | | | | |
| --- | --- | --- | --- | --- | --- | --- | --- | --- | --- | --- | --- | --- | --- | --- | --- | --- | --- | --- | --- | --- |
|  | **Holistic** | | | **Cognitive** | | | **Affective** | | | **Self** | | | | | **Other** | | | | | |
| **Exposure and Model** | **OR**  **(95% CI)** | **P** | **N** | **OR**  **(95% CI)** | **P** | **N** | **OR**  **(95% CI)** | **P** | **N** | **OR**  **(95% CI)** | **P** | | **N** | | **OR**  **(95% CI)** | | **P** | | **N** | |
| **Early Pandemic Self-Isolating Given Suspected COVID-19 Infection** | | | | | | | | | | | | | | | | | | | | |
| Unadjusted | 1.50  (0.81, 2.78) | .194 | 245 | 1.08  (0.60, 1.95) | .802 | 253 | 1.86  (1.00, 3.45) | .050 | 246 | 1.15  (0.62, 2.10) | .661 | 248 | | 1.38  (0.76, 2.49) | | .295 | | 251 | |  |
| Partially Adjusted | 1.21  (0.62, 2.35) | .576 | 245 | 1.49  (0.79, 2.81) | .216 | 253 | 1.39  (0.71, 2.72) | .338 | 233 | 0.81  (0.42, 1.58) | .537 | | 248 | | 1.26  (0.67, 2.38) | | .478 | | 251 | |
| Fully Adjusted | 1.26  (0.64, 2.48) | .500 | 245 | 1.53  (0.81, 2.87) | .188 | 253 | 1.44  (0.73, 2.86) | .295 | 233 | 0.83  (0.42, 1.64) | .587 | | 248 | | 1.31  (0.69, 2.47) | | .413 | | 251 | |
| **Early Pandemic Face-To-Face Contact Outside Household** | | | | | | | | | | | | | | | | | | | | |
| Unadjusted | 0.98  (0.83, 1.16) | .815 | 2411 | 0.86  (0.73, 1.01) | .068 | 2446 | 1.03  (0.88, 1.21) | .711 | 2415 | 1.03  (0.88, 1.22) | .704 | | 2432 | | 1.04  (0.89, 1.23) | | .616 | | 2425 | |
| Partially Adjusted | 0.92  (0.77, 1.09) | .343 | 2411 | 0.88  (0.75, 1.04) | .146 | 2446 | 0.96  (0.81, 1.14) | .661 | 2415 | 0.91  (0.76, 1.08) | .271 | | 2432 | | 1.04  (0.88, 1.23) | | .636 | | 2425 | |
| Fully Adjusted | 0.93  (0.78, 1.11) | .434 | 2411 | 0.88  (0.75, 1.05) | .155 | 2446 | 0.98  (0.82, 1.16) | .805 | 2415 | 0.92  (0.77, 1.10) | .355 | | 2432 | | 1.05  (0.88, 1.24) | | .609 | | 2425 | |
| **Early Pandemic Physical Contact** **Outside Household** | | | | | | | | | | | | | | | | | | | | |
| Unadjusted | 0.95  (0.75, 1.20) | .680 | 2159 | 0.94  (0.74, 1.18) | .582 | 2189 | 0.95  (0.76, 1.20) | .693 | 2163 | 1.02  (0.81, 1.29) | .857 | | 2177 | | 1.11  (0.88, 1.40) | | .364 | | 2172 | |
| Partially Adjusted | 0.92  (0.73, 1.18) | .522 | 2159 | 0.91  (0.72, 1.16) | .459 | 2189 | 0.93  (0.73, 1.18) | .542 | 2163 | 1.00  (0.78, 1.26) | .969 | | 2177 | | 1.08  (0.85, 1.37) | | .516 | | 2172 | |
| Fully Adjusted | 0.93  (0.73, 1.19) | .563 | 2159 | 0.91  (0.71, 1.15) | .418 | 2189 | 0.94  (0.74, 1.19) | .596 | 2163 | 1.01  (0.79, 1.28) | .965 | | 2177 | | 1.08  (0.85, 1.37) | | .539 | | 2172 | |

*Note.* Logistic regressions. OR = odds ratio. CI = confidence interval. Complete case = participants who had complete data on all covariates included in the fully adjusted regression models. Partially adjusted = adjusted for sociodemographic variables (age, gender, education, and keyworker status). Fully adjusted = additionally adjusted for prior mental health and risk behaviour variables (pre-pandemic anxiety, depression, high-risk drinking, smoking, and early pandemic suspected COVID-19 infection). All variables in the models are binary. All risk perception variables were dichotomised at the median.

**Supplementary Table S21**

*Complete Case Prospective Longitudinal Associations between Early Pandemic Risk Behaviours and COVID-19 Risk Perceptions (Mothers)*

|  | **COVID-19 Risk Perceptions (Outcomes)** | | | | | | | | | | | | | | |
| --- | --- | --- | --- | --- | --- | --- | --- | --- | --- | --- | --- | --- | --- | --- | --- |
|  | **Holistic** | | | **Cognitive** | | | **Affective** | | | **Self** | | | **Other** | | |
| **Exposure and Model** | **OR**  **(95% CI)** | **P** | **N** | **OR**  **(95% CI)** | **P** | **N** | **OR**  **(95% CI)** | **P** | **N** | **OR**  **(95% CI)** | **P** | **N** | **OR**  **(95% CI)** | **P** | **N** |
| **Early Pandemic Self-Isolating Given Suspected COVID-19 Infection** | | | | | | | | | | | | | | | |
| Unadjusted | 1.14  (0.60, 2.17) | .694 | 174 | 1.41  (0.75, 2.64) | .287 | 182 | 1.27  (0.67, 2.42) | .466 | 175 | 1.01  (0.53, 1.94) | .969 | 177 | 1.19  (0.63, 2.24) | .595 | 180 |
| Partially Adjusted | 1.20  (0.62, 2.31) | .596 | 174 | 1.50  (0.80, 2.86) | .212 | 182 | 1.41  (0.73, 2.75) | .307 | 175 | 1.15  (0.59, 2.24) | .686 | 177 | 1.31  (0.68, 2.51) | .418 | 180 |
| Fully Adjusted | 1.25  (0.63, 2.49) | .520 | 174 | 1.55  (0.81, 2.98) | .189 | 182 | 1.46  (0.73, 2.91) | .285 | 175 | 1.22  (0.60, 2.48) | .589 | 177 | 1.40  (0.71, 2.78) | .335 | 180 |
| **Early Pandemic Face-To-Face Contact Outside Household** | | | | | | | | | | | | | | | |
| Unadjusted | 0.95  (0.76, 1.20) | .691 | 1331 | 0.96  (0.76, 1.20) | .712 | 1353 | 0.96  (0.75, 1.20) | .666 | 1334 | 0.94  (0.74, 1.18) | .585 | 1342 | 1.08  (0.86, 1.35) | .531 | 1342 |
| Partially Adjusted | 0.96  (0.76, 1.21) | .707 | 1331 | 0.94  (0.75, 1.19) | .616 | 1353 | 0.95  (0.75, 1.20) | .673 | 1334 | 0.95  (0.75, 1.21) | .695 | 1342 | 1.07  (0.85, 1.35) | .559 | 1342 |
| Fully Adjusted | 0.96  (0.76, 1.22) | .740 | 1331 | 0.94  (0.75, 1.19) | .629 | 1353 | 0.96  (0.75, 1.21) | .709 | 1334 | 0.95  (0.74, 1.20) | .649 | 1342 | 1.07  (0.85, 1.36) | .552 | 1342 |
| **Early Pandemic Physical Contact** **Outside Household** | | | | | | | | | | | | | | | |
| Unadjusted | 0.89  (0.65, 1.22) | .455 | 1174 | 0.95  (0.70, 1.31) | .773 | 1194 | 0.86  (0.63, 1.19) | .367 | 1177 | 0.86  (0.63, 1.19) | .376 | 1184 | 1.04  (0.76, 1.42) | .817 | 1184 |
| Partially Adjusted | 0.89  (0.65, 1.23) | .487 | 1174 | 0.93  (0.66, 1.27) | .637 | 1194 | 0.87  (0.63, 1.20) | .395 | 1177 | 0.90  (0.65, 1.25) | .516 | 1184 | 1.03  (0.75, 1.41) | .861 | 1184 |
| Fully Adjusted | 0.91  (0.66, 1.25) | .551 | 1174 | 0.94  (0.68, 1.29) | .690 | 1194 | 0.88  (0.64, 1.22) | .440 | 1177 | 0.90  (0.65, 1.25) | .527 | 1184 | 1.04  (0.76, 1.44) | .790 | 1184 |

*Note.* Logistic regressions. OR = odds ratio. CI = confidence interval. Complete case = participants who had complete data on all covariates included in the fully adjusted regression models. Partially adjusted = adjusted for sociodemographic variables (age, education, and keyworker status). Fully adjusted = additionally adjusted for prior mental health and risk behaviour variables (pre-pandemic anxiety, depression, high-risk drinking, smoking, and early pandemic suspected COVID-19 infection). All variables in the models are binary. All risk perception variables were dichotomised at the median.

**Supplementary Table S22**

*Complete Case Prospective Longitudinal Associations between Early Pandemic Risk Behaviours and COVID-19 Risk Perceptions (Young People)*

|  | **COVID-19 Risk Perceptions (Outcomes)** | | | | | | | | | | | | | | |
| --- | --- | --- | --- | --- | --- | --- | --- | --- | --- | --- | --- | --- | --- | --- | --- |
|  | **Holistic** | | | **Cognitive** | | | **Affective** | | | **Self** | | | **Other** | | |
| **Exposure and Model** | **OR**  **(95% CI)** | **P** | **N** | **OR**  **(95% CI)** | **P** | **N** | **OR**  **(95% CI)** | **P** | **N** | **OR**  **(95% CI)** | **P** | **N** | **OR**  **(95% CI)** | **P** | **N** |
| **Early Pandemic Face-To-Face Contact Outside Household** | | | | | | | | | | | | | | | |
| Unadjusted | 0.93  (0.73, 1.18) | .560 | 1080 | 0.92  (0.70, 1.23) | .584 | 1093 | 0.96  (0.75, 1.22) | .721 | 1081 | 0.86  (0.67, 1.10) | .224 | 1090 | 1.00  (0.78, 1.27) | .953 | 1083 |
| Partially Adjusted | 0.91  (0.71, 1.17) | .483 | 1080 | 0.89  (0.67, 1.19) | .428 | 1093 | 0.95  (0.74, 1.21) | .659 | 1081 | 0.83  (0.64, 1.07) | .157 | 1090 | 0.98  (0.76, 1.27) | .896 | 1083 |
| Fully Adjusted | 0.93  (0.72, 1.20) | .474 | 1080 | 0.89  (0.67, 1.19) | .446 | 1093 | 0.97  (0.75, 1.24) | .788 | 1081 | 0.85  (0.65, 1.10) | .208 | 1090 | 1.00  (0.78, 1.29) | .995 | 1083 |
| **Early Pandemic Physical Contact** **Outside Household** | | | | | | | | | | | | | | | |
| Unadjusted | 1.02  (0.72, 1.45) | .890 | 985 | 1.11  (0.75, 1.65) | .608 | 995 | 1.07  (0.76, 1.53) | .687 | 986 | 1.04  (0.72, 1.48) | .851 | 993 | 1.02  (0.71, 1.45) | .922 | 988 |
| Partially Adjusted | 0.98  (0.68, 1.39) | .891 | 985 | 1.08  (0.72, 1.61) | .715 | 995 | 1.03  (0.72, 1.47) | .889 | 986 | 0.98  (0.68, 1.42) | .928 | 993 | 0.99  (0.69, 1.42) | .949 | 988 |
| Fully Adjusted | 0.95  (0.66, 1.36) | .768 | 985 | 1.03  (0.69, 1.55) | .874 | 995 | 1.02  (0.71, 1.47) | .920 | 986 | 1.00  (0.69, 1.44) | .985 | 993 | 0.97  (0.67, 1.40) | .859 | 988 |

*Note.* Logistic regressions. OR = odds ratio. CI = confidence interval. Complete case = participants who had complete data on all covariates included in the fully adjusted regression models. Partially adjusted = adjusted for sociodemographic variables (age, gender, education, and keyworker status). Fully adjusted = additionally adjusted for prior mental health and risk behaviour variables (pre-pandemic anxiety, depression, high-risk drinking, smoking, and early pandemic suspected COVID-19 infection). All variables in the models are binary except age, which is continuous for the young person cohort. All risk perception variables were dichotomised at the median. Sample sizes were too small for statistical models on early pandemic self-isolating given suspected COVID-19 infection, so these results were removed.
